# Optimal Aspirin Dosage for the Prevention of Preeclampsia and Other Adverse Pregnancy Outcomes: A Systematic Review and Meta-Analysis of Randomized Controlled Trials

**DOI:** 10.3390/jcm14072134

**Published:** 2025-03-21

**Authors:** Balázs Komoróczy, Szilárd Váncsa, Alex Váradi, Péter Hegyi, Veronika Vágási, István Baradács, Anett Szabó, Péter Nyirády, Zsófia Benkő, Nándor Ács

**Affiliations:** 1 Department of Obstetrics and Gynecology, Semmelweis University, 1085 Budapest, Hungary; 2 Centre for Translational Medicine, Semmelweis University, 1085 Budapest, Hungary; 3 Institute for Translational Medicine, Medical School, University of Pécs, 7622 Pécs, Hungary; 4 Institute of Pancreatic Diseases, Semmelweis University, 1085 Budapest, Hungary; 5 Department of Metagenomics, University of Debrecen, 4032 Debrecen, Hungary; 6 Department of Laboratory Medicine, University of Pécs, 7622 Pécs, Hungary; 7 Department of Urology, Semmelweis University, 1085 Budapest, Hungary

**Keywords:** aspirin, pregnancy, meta-analysis, prophylaxis, preeclampsia

## Abstract

**Background/Objectives**: This systematic review and meta-analysis aimed to determine the effectiveness of different aspirin dosages in preventing preeclampsia and its effect on other pregnancy-associated conditions. **Methods**: A comprehensive search of three databases (Pubmed, Embase, and Cochrane Library) was conducted for randomized controlled trials without time interval criteria, comparing aspirin at various doses with placebo or no specific preeclampsia prophylaxis. Eligible randomized controlled trials (RCTs) examined pregnant women receiving aspirin at any dose and time during their pregnancy, while the control group received a placebo, or placebo and a different dose of aspirin, or no specific preeclampsia prevention. No exclusion criteria were established regarding the population, study size, study site, or length of aspirin prophylaxis. Studies examining additional preventive medication (such as low-molecular-weight heparin) compared to aspirin without a placebo group were excluded. For all outcomes, the risk ratios (RRs) and mean differences (MDs) with 95% confidence intervals (CIs) were calculated. Meta-regression was performed to examine the relation between aspirin dosage and preeclampsia. **Results**: Based on the analysis of 31 studies involving 28,318 pregnancies and 20 studies involving 26,551 pregnancies, the early initiation of aspirin significantly reduced the overall incidence of preeclampsia (RR = 0.63, CI: 0.47–0.84) and perinatal death risk (RR = 0.82, CI: 0.72–0.93), respectively. Based on our meta-regression model, we could not establish a dose-dependent correlation between aspirin dosage and the risk of preeclampsia. **Conclusions**: Early-initiated aspirin prophylaxis is effective in preventing preeclampsia, without raising the incidence of placental abruption or increasing the amount of peripartum bleeding. No specific dose was superior to others; thus, further research should explore higher doses and focus on preterm preeclampsia, maternal–fetal complications, and bleeding.

## 1. Introduction

Preeclampsia is a pregnancy-related condition defined by new onset hypertension and significant proteinuria or end-organ dysfunction without proteinuria, after 20 weeks of gestation or postpartum, and is associated with different fetal and maternal complications [1].

Preeclampsia is the leading cause of maternal morbidity and mortality among all pregnancy-associated hypertensive disorders [1,2]. By affecting 5% to 7% of all pregnancies, it is responsible for over 70,000 maternal and 500,000 fetal deaths annually. Furthermore, preeclampsia is also the leading cause of cesarean section and prematurity in the United States [3,4,5].

Aspirin is a widely available and affordable medication with the potential to safely help pregnant women and neonates during pregnancy by preventing preeclampsia and its complications [6]. The first study examining aspirin as a possible preeclampsia prevention remedy was carried out in 1985 by Beaufils et al. [7], and since then, several randomized trials have investigated the prophylactic use of aspirin in preventing preeclampsia. However, over the years, different doses of aspirin from 25 mg to 150 mg were assessed in numerous well-designed trials with different baseline characteristics, examining different safety and efficacy outcomes, but the optimal dose still remains unclear. Based on the data provided, the timing of initiation for preeclampsia prevention is suggested at ≥12 weeks of gestation and ideally before 16 weeks [8]. Although the United States Preventive Services Taskforce (USPSTF) and the American College of Obstetricians and Gynecologists (ACOG) also recommend 81 mg of aspirin daily based on statistical analyses, recent meta-analyses [9,10] suggest that higher doses may be more efficient without elevating the risk of maternal and fetal adverse outcomes [11]. Likewise, for women identified as high risk, the recommendation delivered by The International Federation of Gynecology and Obstetrics (FIGO) in 2020 also suggests aspirin prophylaxis commencing at 11–14 weeks plus 6 days of gestation at a dose of ~150 mg until 36 weeks of gestation, when delivery occurs, or when preeclampsia is diagnosed [12].

In light of the controversies, our primary objective was to determine the effect of different aspirin doses on the incidence of preeclampsia. Additionally, we aimed to assess its impact on several secondary outcomes, including intrauterine growth restriction, preterm birth, gestational age at delivery, actual birth weight (neonate’s recorded weight at delivery explicitly excluding common alternative weight estimates, such as ultrasound-based fetal weight estimations, modeled projections, or retrospective estimations), placental abruption, peripartum bleeding, neonatal intensive care admission, and perinatal death, in which we did not separate intrauterine demise from early neonatal death. We hypothesized that higher doses of aspirin are more effective in preventing preeclampsia without raising the risk of fetal–maternal complications.

## 2. Materials and Methods

We reported our meta-analysis based on the recommendation of the PRISMA 2020 guideline [13] (see Supplementary Table S1), while we followed the Cochrane Handbook [14]. The study protocol was registered on PROSPERO (registration number CRD42021287369). In comparison to the original protocol, instead of a network meta-analysis, we performed a meta-analysis with a dose–response analysis to characterize the dose-dependent effect of aspirin on preeclampsia incidence.

### 2.1. Search Strategy

We conducted our systematic search in three databases (Pubmed, Embase, and Cochrane Library) on 3 November 2021 and updated on 29 January 2023, with the following search key: (aspirin OR “acetylsalicylic acid” OR ASA) AND (pregnancy OR pregnant OR gravidity OR preeclampsia OR eclampsia OR “preterm delivery” OR “preterm birth” OR “premature delivery” OR “premature birth” OR “fetal growth restriction” OR FGR OR “intrauterine growth restriction” OR IUGR OR “small for gestational age” OR SGA) AND random*. We did not use language, study type, or other filters during the search.

### 2.2. Selection Process

For the selection process, we used the Endnote v20 reference manager software (Clarivate Analytics, Philadelphia, PA, USA). After automatic and manual duplicate removal, we screened each entry based on title and abstract and then based it on the full text. The selection was made on each level by two independent review authors, while a third independent review author resolved disagreements.

### 2.3. Eligibility Criteria

Eligible randomized controlled trials (RCTs) examined pregnant women receiving aspirin at any dose and time during their pregnancy, while the control group received a placebo, a different dose of aspirin, or no specific preeclampsia prevention. Our primary outcome was preeclampsia, while we analyzed numerous secondary outcomes (see details below). No exclusion criteria were established regarding the population, study size, study site, or length of aspirin prophylaxis. We did not exclude studies due to initiation time differences. In addition, studies examining more doses of aspirin compared to placebo were also included. Studies examining additional preventive medication (such as low-molecular-weight heparin) compared to aspirin without a placebo group were excluded.

### 2.4. Data Collection Process and Data Items

Using a predefined datasheet, the following data were extracted: first author, year of publication, study population, gestational age at randomization, eligibility criteria, time and dosage of aspirin, type of control, and the total number of patients and events in the intervention and control groups.

### 2.5. Outcome Definitions

We checked each study’s outcome definitions and categorized them accordingly. Studies were included only if they explicitly reported outcome definitions, ensuring consistency in data extraction and analysis. For preeclampsia, we accepted definitions that included significant proteinuria or end-organ dysfunction without proteinuria [15]. For intrauterine growth restriction (IUGR), outcomes were classified based on different birth weight percentile thresholds, specifically evaluating below the 10th, 5th, and 3rd percentiles separately. Studies that did not specify a precise IUGR definition were included in the overall analysis. For preterm births, we applied a cut-off of delivery before 37 weeks. Birth weights were reported in grams, and gestational age was recorded in weeks. When studies originally reported gestational age in days, values were converted into weeks with two-decimal precision for consistency. Placental abruption was defined based on clinical symptoms or the pathological examination of the placenta. For perinatal death, we included both intrauterine fetal demise and neonatal mortality, defined as death within the first 28 days after delivery. Additionally, postpartum hemorrhage was analyzed as a separate safety outcome. These outcome definitions were applied consistently across all included studies to maintain methodological rigor and comparability.

### 2.6. Risk of Bias Assessment

Two authors independently assessed the risk of bias by utilizing the Cochrane risk-of-bias tool for randomized trials (RoB 2) tool [16]. A third review author resolved disagreements.

### 2.7. Synthesis Methods

All statistical analyses were made with R (R Core Team 2020, v4.0.3) using the meta (v5.2.0) package [17]. We calculated risk ratios (RRs) with 95% confidence intervals (CIs) for categorical variables, while for continuous variables, we calculated mean differences (MDs) with 95% CIs. The restricted maximum likelihood (REML) method was applied with a random-effects model [18]. We used forest plots to represent pooled and individual study results. I^2^ and χ^2^ tests assessed the statistical heterogeneity with a *p*-value < 0.1 as a threshold for a statistically significant difference. If at least ten studies were involved in the analysis, Egger’s test was used to assess publication bias. In addition to heterogeneity, a *p*-value < 0.05 was considered statistically significant in our analysis. We performed a meta-regression using a random-effects model to test the effects of different aspirin doses on the effect size. We used scatter plots to present individual study estimates and calculated the residual heterogeneity (I^2^ and χ^2^) and regression coefficients with a 95% CI. Subgroup analyses were carried out for all the different doses of daily aspirin. Furthermore, we divided the included studies based on the treatment’s start.

### 2.8. Evidence Synthesis

We followed the recommendation of the Grades of Recommendation, Assessment, Development, and Evaluation (GRADE) workgroup to evaluate the quality of evidence [19].

## 3. Results

### 3.1. Search and Selection

**Figure 1:**
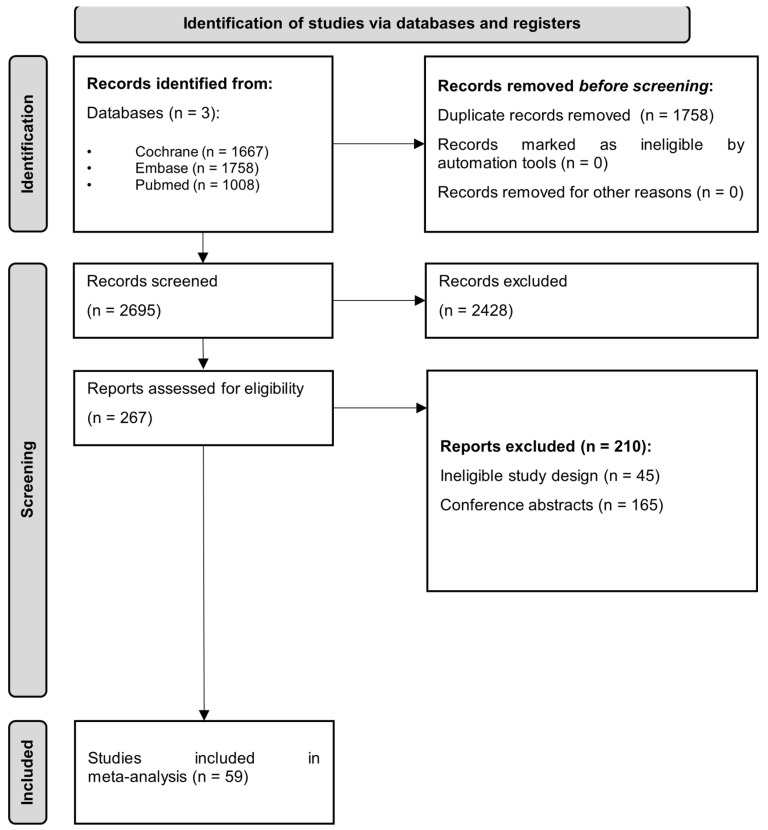
PRISMA 2020 flowchart representing the study selection process.

Using our search key, we identified 4433 studies. After duplicate removal, we screened 2675 articles based on title and abstract. Finally, we found 59 full-text articles eligible out of the 267 full-text studies assessed (Figure 1).

### 3.2. Basic Characteristics of Included Studies

The baseline characteristics of the enrolled studies are detailed in Table 1. All the RCTs identified by our systematic search were involved, in which aspirin doses between 25 and 150 mg were compared to a placebo or the absence of any prophylaxis, and the initiation of aspirin varied from preconception to the third trimester. Among all involved articles, fifty-four examined moderate-to-high-risk preeclampsia pregnancies; three examined patients with unexplained recurring pregnancy loss; one examined patients with Alpha Fetoprotein levels below 2.5 without fetal anomalies; and one did not set up additional inclusion criteria for pregnancy for the examined population. Supplementary Tables S1 and S2 summarize the inclusion and exclusion criteria for the eligible studies.

### 3.3. Primary Outcomes

We summarized our results in Table 2. The detailed analysis for each analysis can be found in Supplementary Figures S2–S26. For the outcome of preeclampsia, early-initiated aspirin showed the most convincing results. As a result of the analysis of 31 studies involving 28,318 pregnancies, early-initiated aspirin significantly lowered the risk of preeclampsia (RR = 0.63, CI: 0.47–0.84) (Figure 2). As a result of the meta-regression analysis between the different aspirin doses and diagnosed preeclampsia (Figure 3), the correlation coefficient was −0.0006 (CI: −0.0012, 0, *p* = 0.049), with a high residual heterogeneity (I^2^ = 99.53%, *p* < 0.001), which means that the effect of the dose is insignificant. Aspirin initiated after the 20th week of gestation did not have a significant effect on preeclampsia prevention (RR = 0.67, CI: 0.35–1.28).

### 3.4. Secondary Outcomes

**Figure 4:**
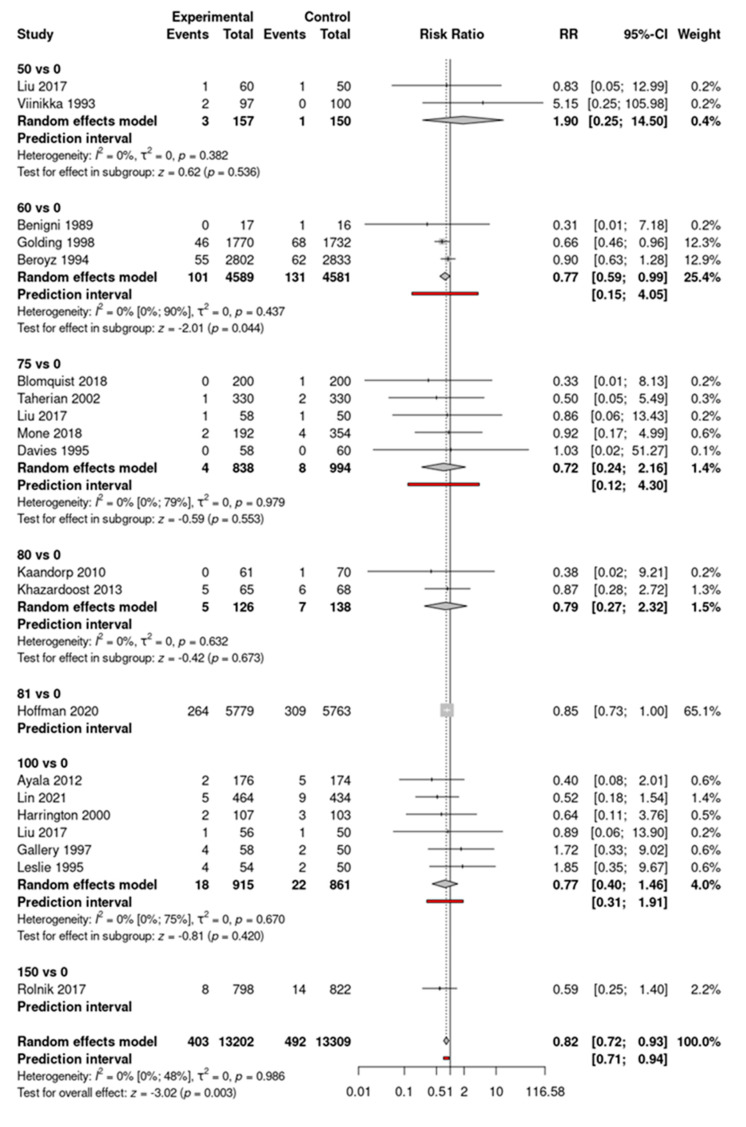
Forest plot of the perinatal death outcome with early-initiated (<week 20) aspirin [20,21,24,25,30,31,34,36,37,38,39,40,44,45,46,48,49,50,52,53,54,56,60,62,63,65,66,74].

**Figure 5:**
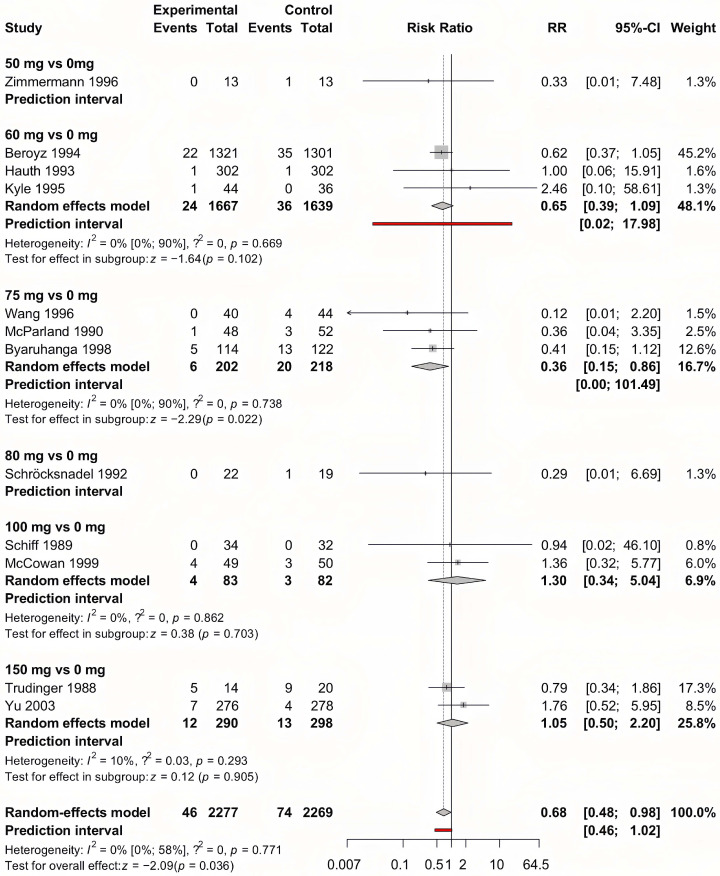
Forest plot of the perinatal death outcome with late-initiated (>week 20) aspirin [24,59,64,67,68,69,70,71,73,76,77,78].

In the overall analysis, no significant risk reduction for IUGR was found in the aspirin group compared to the control group. In the case of early-initiated aspirin, lower rates were found (RR = 0.91, CI: 0.083–1.00, *p* = 0.055), but the significance level was not reached. Based on subgroup analyses, only 150 mg of aspirin was associated with significant risk reduction (RR = 0.72, CI 0.55–0.96 and RR = 0.83, CI: 0.70–0.99) in cases of fetal weight below the 10th and 5th percentile, respectively. No significant effect was described for the outcome of more severe IUGR (<3rd percentile). Although for the outcome of preterm birth <37 weeks, there were no significant differences in the risks between the two groups (RR = 0.82, CI: 0.65–1.03), and patients receiving aspirin carried their pregnancies significantly longer by an average of 0.26 weeks and gave birth to a significantly—averagely 27.56 g—heavier child than patients receiving no prophylaxis (MD = 0.26, CI: 0.05–0.46, and MD = 27.56, CI: 5.09–50.04, respectively). On the other hand, in the set up of late aspirin initiation (>week 20), a significant reduction was described in the rates of preterm birth (RR = 0.79, CI: 0.70–0.91), without a significant effect on birth weight (MD = 0.25, CI: −0.38–0.87) or gestational age (MD = 42.37, CI: −15.96–100.70). No significant difference in the incidence of placental abruption or postpartum hemorrhage was described between the two groups (RR = 1.13, CI: 0.92–1.39) and (RR = 1.13, CI: 0.95–1.34), respectively. Although the necessity of the neonatal intensive care unit admission of newborns was not influenced by prophylactic aspirin usage (RR = 0.96, CI: 0.86–1.06), newborns of those who were allocated to receive aspirin had a significantly reduced risk for perinatal death (RR = 0.86, CI: 0.77–0.96). For the outcome of perinatal death, significantly reduced risks can also be noted in patients who started aspirin before or after the 20th week (RR = 0.82, CI: 0.72–0.93 and RR = 0.68, CI: 0.48–0.98, respectively) (Figure 4 and Figure 5).

### 3.5. Publication Bias

Egger’s test showed a significant publication bias for overall birth weight (*p* = 0.043), overall IUGR (*p* = 0.023), and gestational age for aspirin started before 20 weeks (*p* = 0.019).

### 3.6. Risk of Bias Assessment and Evidence Synthesis

Overall, no studies were deemed to have a high risk for bias, six studies were rated to raise some concerns, and the remaining fifty-three studies were evaluated to have a low risk of bias. The results of the risk of bias assessment are presented in Supplementary Table S3. Evidence certainty of all our examined outcomes was graded as high evidence (Table 2 and Supplementary Table S4).

## 4. Discussion

### 4.1. Main Findings

In correlation with prior meta-analyses [10,11,79], we found that aspirin started before the 20th week significantly reduced the risk of preeclampsia and perinatal death [79] in both low- and high-risk pregnancies. This effect was consistent across various study populations and remained robust even in sensitivity analyses. Based on the meta-regression analyses, the dose of aspirin was not significantly associated with the risk of preeclampia. The observed reduction in preeclampsia risk aligns with the hypothesis that early aspirin initiation counteracts placental dysfunction, a key pathophysiological mechanism underlying the disease. As placentation is largely completed by the end of the first trimester, initiating aspirin prophylaxis before the 20th week may enhance trophoblastic invasion and improve uteroplacental perfusion. This mechanistic basis is supported by studies demonstrating that aspirin modulates thromboxane–prostacyclin balance, mitigates oxidative stress, and restores angiogenic homeostasis in high-risk pregnancies.

Despite the clear benefits of early initiation, our findings indicate no significant dose–response relationship, reinforcing the idea that lower doses may be equally effective in preeclampsia prevention when administered at the optimal time. This is consistent with prior clinical recommendations that emphasize initiation timing over dose escalation. However, residual heterogeneity across studies highlights the need for further research to identify potential subgroups that might benefit from higher doses or alternative risk stratification methods.

### 4.2. Additional Findings

Regarding secondary outcomes, we found different effects with a wide variety of significant and non-significant results (see Supplementary Figures S2–S24). For the outcome of IUGR, a significant risk reduction was only described in the overall group if IUGR was defined as below the 5th percentile. We found that aspirin prophylaxis does not affect the incidence rate of severe IUGR (IUGR below the 3rd percentile) and the intensive care admission of newborns. These data are not supported by previous meta-analyses, which suggest aspirin as a potential prophylaxis for IUGR [6,79]. However, the ASPIRIN trial [38] linked early-initiated aspirin prophylaxis with significant preterm birth reduction, and it has been strengthened by the meta-analysis of Yeo et al. 2021 [79]; we did not find a significant correlation between aspirin prophylaxis and preterm birth incidence only in the subgroup of late-initiated aspirin. These beneficial effects of late initiation should be evaluated carefully by considering that spontaneous and iatrogenic preterm births were not distinguished in the analysis of the involved trials. Prophylaxis significantly prolonged pregnancies by 0.26 weeks, and the actual birth weight of newborns was significantly higher, with 27.56 g on average. This correlates with the findings of an earlier-mentioned meta-analysis [80], with the difference being that the effect on gestational age did not reach the significance level earlier.

During the peer review process, a new randomized controlled trial by Mirzamoradi et al. was published, investigating low-dose aspirin (80 mg) for preterm delivery (PTD) prevention in women with a history of spontaneous PTD. While aspirin did not significantly reduce overall PTD rates, it showed a protective effect in a high-risk subgroup experiencing spontaneous labor (*p* = 0.022). These findings further highlight the heterogeneous effects of aspirin on PTD, suggesting a potential role in targeted prophylaxis.

### 4.3. Safety

With all these beneficial effects, we have to take safety into account. At doses more than 100 mg/day, aspirin irreversibly inhibits COX-1 and COX-2. Although blocking prostaglandin production, suppressing the immune system, and diminishing oxidative stress might restore the angiogenic imbalance associated with preeclampsia [80,81], safety considerations must always be considered. In the case of placental abruption, which has been attributed to ischemia–reperfusion injury in maternal uteroplacental vessels as a possible complication of aspirin intake, increased risk ratios were found in the aspirin group, but it did not reach the level of significance. Referring to a prior meta-analysis—examining low-risk nulliparous women with singleton pregnancies—conducted by Man et al. in 2021 [11], our study showed similar results for postpartum bleeding, which did not reach the level of significance. We did not examine the amount of blood loss during delivery. Due to a lack of quantified information, we did not perform a statistical analysis of antepartum bleeding.

### 4.4. Strengths and Limitations

Regarding the strengths of our study, this assessment was the first meta-regression analysis of aspirin for preeclampsia prevention, evaluating a large sample size. Subgroup analyses were also conducted to determine the optimal dose of prophylactic aspirin. The limitations of this work include heterogeneity in outcome definitions, which may introduce some inconsistency in the findings. Additionally, some studies did not provide clearly defined outcomes, further contributing to variability. We did not separately evaluate preterm preeclampsia (preeclampsia before the 34th week of pregnancy) or preterm birth before the 34th week of gestation. Due to limited data and inconsistent reporting, antepartum bleeding was not assessed. In our study, iatrogenic and spontaneous preterm births were not distinguished, and the risk factors for preterm birth were not evaluated, which likely varied across study sites. In studies where multiple aspirin doses were compared to placebo, to prevent the distortion of outcomes, we excluded additional dose groups beyond the first comparison to avoid counting the same placebo group multiple times. The selection of which dose to include in such cases was randomized. Additionally, the moderate risk of bias in some domains represents another limitation. Despite these constraints, aspirin prophylaxis was effective in preventing preeclampsia and significantly lowered the incidence of perinatal death, especially when initiated before 20 weeks of pregnancy. Different doses showed effectiveness for various outcomes, but no clear dose dependency was observed across the examined endpoints.

### 4.5. Implications for Practice and Research

In agreement with the leading professional societies [82,83,84], we suggest aspirin prophylaxis at a dose of 81 g to 150 mg for high-risk pregnancies [85] with the initiation before 16 but not earlier than 11 weeks of gestation, until 36 weeks of gestation, when delivery occurs, or when preeclampsia is diagnosed. We encourage clinicians to screen for high-risk pregnancies available to start aspirin prophylaxis with a dose preferably between 81 mg and 150 mg. Based on these findings, preeclampsia and perinatal mortality can be successfully reduced. By taking into account the international guidelines [82,83,84], and the examined dose–response correlation between aspirin dose and preeclampsia incidence, we suggest further RCTs with higher doses of aspirin compared to the widely accepted 81 mg, focusing on preterm preeclampsia, fetal–maternal complications, and bleeding. We suggest involving pediatricians to evaluate fetal hemorrhagic conditions, and for the clinical obstetricians to set up a reliable measurement method for evaluating peripartum bleeding in both vaginal and abdominal delivery cases.

## Figures and Tables

**Figure 2:**
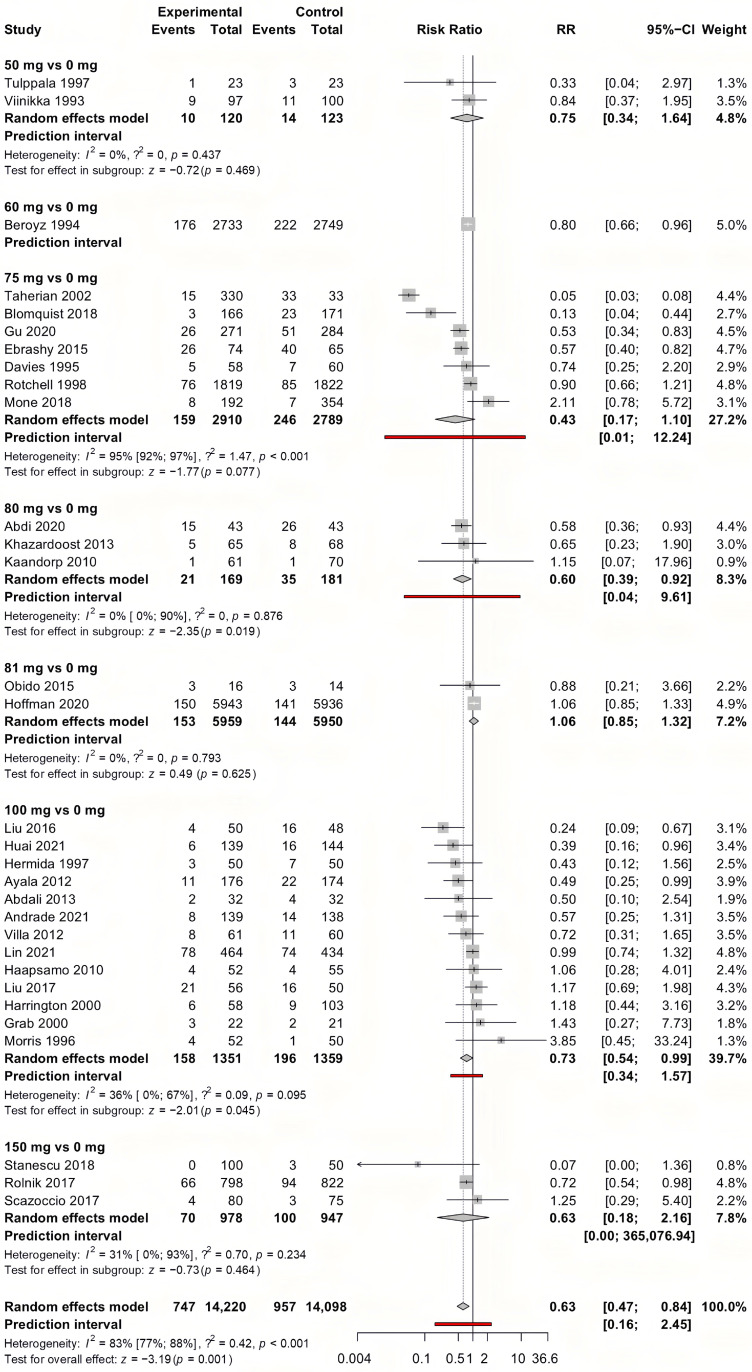
Forest plot of the outcome preeclampsia with early-initiated (<week 20) aspirin. The experimental group received the indicated aspirin dose (e.g., “100 vs. 0” refers to 100 mg of aspirin vs. placebo or no aspirin). The control group consists of participants who received either a placebo or no specific preeclampsia prevention. Each row represents an individual study, with the number of events and total participants in both groups. The risk ratio (RR) and 95% confidence interval (CI) for each study are displayed, with the summary effect estimated using a random-effects model. Prediction intervals indicate the expected range of effects in future studies. Statistical heterogeneity is quantified using I^2^ and τ^2^ values [20,21,24,25,30,31,34,36,37,38,39,40,44,45,46,48,49,50,52,53,54,56,60,62,63,65,66,74].

**Figure 3:**
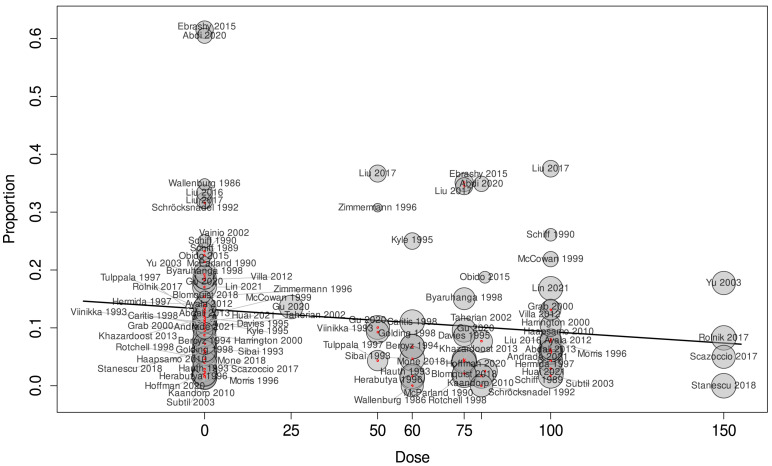
Meta-regression between the administered dosage and the effect size of each individual study for the outcome of preeclampsia started at any time during pregnancy [20,21,22,24,25,26,30,31,33,34,35,36,37,38,39,40,44,45,46,48,49,50,52,53,54,55,56,57,58,60,61,62,63,64,65,66,67,68,69,70,71,72,73,74,75,77,78].

**Table 1:** Basic characteristics of included studies.

Author (Year)	Study Site	Population	No. of Analyzed Patients (I/C), Completion %	Aspirin Dosage and Administration Period	Control	Outcomes
Aspirin Initiation Before 20 Weeks						
Abdali 2013 [20]	Iran	high risk for PE or for gestational hypertension	32/32 100%	100 mg/day (12–16 w)–32 w	Placebo	PE, IUGR, PTD
Abdi 2020 [21]	Iran	high risk for PE	43/43 96%	80 mg/day (12–15 w)–36 w	Placebo	PE, IUGR, PTD, GA, BW
Andrade 2021 [22]	Portugal	moderate to high risk for PE	139/138 90%	100 mg/day (11–14 w)–34 w	Placebo	PE
Benigni 1989 [23]	Italy	high risk for PE	17/16 100%	60 mg/day 12 w–until delivery	Placebo	PD, GA, BW, PND
Beroyz 1994 [24]	Multicentric	high risk for PE	4659/4650 99%	60 mg/day 12–32 w–until delivery	Placebo	PE, PTD, IUGR, PND, BW, GA, PA, NICA
Blomqvist 2018 [25]	Sweden	habitual abortion in history	200/200 100%	75 mg/day (5–6 w)–36 w	Placebo	PE, IUGR, PTD, PND, NICA
Caritis 1998 [26]	Multicentric	high risk for PE	1254/1249 99%	60 mg/day 13–26 w–until delivery	Placebo	PE
Caspi 1994 [27]	Israel	high risk for PE	24/23 100%	100 mg/day 15–23 w–until delivery	Placebo	IUGR, PTD, BW, GA, PND, PA
Chiaffarino 2004 [28]	Italy	high risk for PE	16/19 88%	100 mg/day <14 w–until delivery	No treatment	GA, BW
Dasari 1998 [29]	India	moderate to high risk for PE	25/25 100%	100 mg/day 12 w–36 w	Placebo	GA, BW
Davies 1995 [30]	United Kingdom	moderate to high risk for PE	58/60 97%	75 mg/day 18 w–until delivery	Placebo	PE, IUGR, BW, GA, PA, PND, NICA
Ebrashy 2015 [31]	Egypt	high risk for PE or IUGR and abnormal uterine flow	74/65 98%	75 mg/day 14–16 w–37 w	No treatment	PE, IUGR, PTD, BW
Gallery 1997 [32]	Australia	high risk for PE	58/50 100%	100 mg/day 17–19 w–until 2 weeks prior to planned delivery	Placebo	PTD, PND
Golding 1998 [33]	Jamaica	moderate to high risk for PE	3023/3026 97%	60 mg/day 12–32 w–until delivery	Placebo	PE, PTD, BW, PND, NICA
Gu 2020 [retracted] ^†^	China	high risk for PE	272/278/ 271/284 95%	25 mg/50 mg/75 mg/day 12 w–until delivery	Placebo	PE, IUGR, PTD, PA
Haapsamo 2010 [34]	Finland	high risk for PE	52/55 22%	100 mg/day from the day of gonadotropin stimulation–until delivery	Placebo	PE, IUGR, BW, GA
Herabutya 1996 [35]	Thailand	moderate to high risk for PE	651/697 90%	60 mg/day 18–24 w–no data	Placebo	PE
Hermida 1997 [36]	Spain	high risk for PE	50/50 100%	100 mg/day 12–16 w–until delivery	Placebo	PE, IUGR, PTD, BW, GA
Hoffman 2020 [37]	Multicentric	moderate to high risk for PE	5787/5771 97%	81 mg/day 6–13 w–37 w	Placebo	PE, PTD, BW, GA
Huai 2021 [38]	China	low to high risk for PE	139/144 100%	100 mg/day 12–20 w–until delivery	Placebo	PE, PTD, PA, IUGR
Kaandorp 2010 [39]	Netherlands	low to high risk for PE	99/103 84%	80 mg/day 0–6 w–36 w	Placebo	PE, IUGR, PTD, GA, PA, PND
Khazardoost 2013 [40]	Iran	low to high risk for PE	65/68 95%	80 mg/day 15–18 w–32 w	No treatment	PE, IUGR, PTD, PND, NICA
Lambers 2009 [41]	Netherlands	high risk for PE	28/26 100%	100 mg/day preconceptional–until 12 w	Placebo	PE, PTD, IUGR
Landman 2022 [42]	Netherlands	high risk for PE	194/193 95%	80 mg/day 8–16 w–until delivery	Placebo	PTD, GA, BW, IUGR
Leslie 1995 [43]	Australia	high risk for PE	54/50 90%	100 mg/day 17–19 w–until 2 weeks prior to planned delivery	Placebo	BW, GA, PND, NICA
Lin 2021 [44]	China	high risk for PE	464/434 91%	100 mg/day 12–20 w–until 32 w	No treatment	PE, IUGR, PTD, BW, GA, PA, PND, NICA
Liu 2016 [45]	China	high risk for PE	50/48 92%	100 mg/day 12 w–until delivery	Placebo	PE, GA
Liu 2017 [46]	China	high risk for PE	60/58/56/50 100%	50 mg, 75 mg and100 mg/day 16 w–until delivery	Placebo	PE, GA, PA, PND
Louden 1992 [47]	United Kingdom	moderate to high risk for PE	10/8 100%	60 mg/day 16 w–until delivery	Placebo	IUGR, BW, GA
Mone 2018 [48]	Ireland	moderate risk for PE	179/183 100%	75 mg/day 11–14 w–until delivery	No treatment	PE, PTD, PND, NICA
Morris 1996 [49]	Australia	high risk for PE	52/50 98%	100 mg/day 17–19 w–until delivery	Placebo	PE, IUGR, PTD, BW
Odibo 2015 [50]	USA	high risk for PE	16/14 57%	81 mg/day 11–14 w–until 37 w	Placebo	PE, IUGR
Pattison 2000 [51]	New Zealand	high risk for PE	20/20 100%	75 mg/day 4–13 w–until delivery	Placebo	IUGR, PTD, BW, NICA
Rolnik 2017 [52]	Multicentric	high risk for PE	798/822 (91%)	150 mg/day 11–14 w–36 w	Placebo	PE, IUGR, PTD, PA, PND, NICA
Rotchell 1998 [53]	Barbados	low to high risk for PE	1834/1841 (99,8%)	75 mg/day 12–32 w–until delivery	Placebo	PE, BW, GA, PA, PND, NICA
Scazzoccio 2017 [54]	Spain	high risk for PE	80/75 (83%)	150 mg/day 11–14 w–28 w	Placebo	PE, IUGR, BW, GA
Sibai 1993 [55]	USA	moderate to high risk for PE	1485/1500 (95%)	60 mg/day 13–25 w–until delivery	Placebo	PE, IUGR, BW, GA, PND, NICA
Stanescu 2018 [56]	Romania	high risk for PE and fetal growth restriction	100/50 (100%)	150 mg/day at average of 12.4 w–until 32 w and 36 w	Placebo	PE, IUGR
Subtil 2003 [57,58]	France	moderate to high risk for PE	1634/1640 (99%)	100 mg/day 14–21 w–34 w	Placebo	PE, IUGR, BW, GA, PA, PND, NICA
Trudinger 1988 [59]	Australia	high risk for PE	14/20 (100%)	150 mg/day 18–36 w–until delivery	Placebo	BW, GA, PND, NICA
Tulppala 1997 [60]	Finland	low to high risk for PE	23/23 (53%)	50 mg/day 5–22 w–w34	Placebo	PE, IUGR, BW, GA
Vainio 2002 [61]	Finland	high risk for PE	43/43 (96%)	0,5 mg/kg/day 12–14 w–until delivery	Placebo	PE, IUGR, BW, GA
Viinikka 1993 [62]	Finland	high risk for PE	97/100 (95%)	50 mg/day 12–18 w–until delivery	Placebo	PE, IUGR, BW, GA, PND, NICA
Villa 2012 [63]	Finland	moderate to high risk for PE	61/60 (80%)	100 mg/day 12–14 w–until 35 w	Placebo	PE, IUGR
Aspirin Initiation After 20 Weeks						
Byaruhanga 1998 [64]	Zimbabwe	high risk for PE	113/117 92%	75 mg/day 20–28 w–38 w	Placebo	PE, IUGR, PTD, GA, BW, PA, PND, NICA
Grab 2000 [65]	Germany	high risk for PE	22/21 100%	100 mg/day 20 w–until delivery	Placebo	PE, BW, GA
Harrington 2000 [66]	United Kingdom	high risk for PE	107/103 100%	100 mg/day 24–26 w–37 w	No treatment	PE, IUGR, BW, GA, PA, NICA, PND
Hauth 1993 [67]	United States	moderate to high risk for PE	302/302 99%	60 mg/day 24 w–until delivery	Placebo	PE, IUGR, PTD, PND, BW
Kyle 1995 [68]	United Kingdom	moderate to high risk for PE	44/36 100%	60 mg/day 27–29 w–32 w	Placebo	PE, BW, GA, PND
McCowan 1999 [69]	New Zealand	high risk for IUGR	32/33 100%	100 mg/day 24–36 w–until delivery	Placebo	PE, IUGR, BW, GA, PND, NICA
McParland 1990 [70]	United Kingdom	high risk for PE	52/48 94%	75 mg/day 24 w–until delivery	Placebo	PE, BW, GA, PND
Schiff 1989 [71]	Israel	high risk for PE	34/31 (100%)	100 mg/day 28–29 w–until 10 days prior to estimated delivery	Placebo	PE, IUGR, PTD, BW, GA, PND, NICA
Schiff 1990 [72]	Israel	high risk for PE	23/24 (100%)	100 mg/day 30–36 w–until 5 days prior to estimated delivery	Placebo	PE, BW, GA
Schröcksnadel 1992 [73]	Germany	moderate to high risk for PE	22/19 (100%)	80 mg/day 28–32 w–until 37 w	Placebo	PE, IUGR, PTD, BW, GA, PND, NICA
Taherian 2002 [74]	Iran	moderate to high risk for PE	330/330 (100%)	75 mg/day 20 w–until delivery	No treatment	PE, IUGR, PTD, BW, PND
Wallenburg 1986 [75]	Netherlands	high risk for PE	21/23 (96%)	60 mg/day 28 w–until delivery	Placebo	PE, IUGR, BW
Wang 1996 [76]	China	high risk for IUGR	40/44 (100%)	100 mg/day 28–30 w–until 34 w	Placebo	IUGR, PTD, BW, GA, PND
Yu 2003 [77]	Multicentric	high risk for PE	276/278 (99%)	150 mg/day 22–24 w–until 36 w	Placebo	PE, IUGR, PTD, PA, PND, NICA
Zimmermann 1996 [78]	Finland	high risk for hypertensive disorders of pregnancy or IUGR	13/13 (100%)	50 mg/day 22–24 w–until 38 w	No treatment	PE, IUGR, PTD, BW, GA, PA, PND

Abbreviations: PE—preeclampsia; IUGR—intrauterine growth restriction; PTD—preterm delivery; BW—birth weight; GA—gestational age; PND—perinatal death; NICA—neonatal intensive care admission; PA—placental abruption. Retracted articles are marked with ^†^ symbol, as they existed at the time of the systematic search.

**Table 2:** Summary of findings of aspirin prophylaxis at any dose.

Outcome	No. of Studies/Participants	RR or MD (95% CI)	Certainty of Evidence
Overall			
Preeclampsia	47/48,080	0.67 (0.53 to 0.85)	High
IUGR (10%)	26/12,172	0.90 (0.83 to 1.00)	High
IUGR (5%)	7/2870	0.84 (0.70 to 1.00)	High
IUGR (3%)	5/14,031	0.99 (0.66 to 1.47)	High
Preterm birth	29/33,933	0.82 (0.65 to 1.03)	High
Gestational age at delivery (weeks)	31/23,095	0.26 (0.05 to 0.46)	High
Actual birth weight (grams)	34/27,178	27.56 (5.09 to 50.04)	High
Placental abruption	14/20,698	1.13 (0.91 to 1.40)	High
NIC admission	23/31,009	0.96 (0.86 to 1.06)	High
Perinatal death	36/42,778	0.86 (0.77 to 0.96)	High
Postpartum hemorrhage	15/38,311	1.13 (0.95 to 1.34)	High
Aspirin started <20 weeks			
Preeclampsia	31/28,318	0.63 (0.47 to 0.84)	High
IUGR (10%)	19/8250	0.92 (0.82 to 1.04)	High
IUGR (5%)	5/2161	0.88 (0.68 to 1.15)	High
IUGR (3%)	3/9099	1.00 (0.56 to 1.77)	High
Preterm birth	20/26,190	0.83 (0.60 to 1.14)	High
Gestational age at delivery (weeks)	16/2800	0.19 (0.04 to 0.35)	High
Actual birth weight (grams)	16/2786	62.88 (3.75 to 122.00)	High
Perinatal death	20/26,511	0.82 (0.72 to 0.93)	High
Aspirin started >20 weeks			
Preeclampsia	12/4348	0.67 (0.35 to 1.28)	High
IUGR (10%)	5/804	0.95 (0.65 to 1.37)	High
Preterm birth	8/4097	0.79 (0.70 to 0.91)	High
Gestational age at delivery (weeks)	9/772	0.25 (−0.38 to 0.87)	High
Actual birth weight (grams)	9/1317	42.37 (−15.96 to 100.70)	High
Perinatal death	12/4548	0.68 (0.48 to 0.98)	High

Abbreviations: CI: confidence interval, IUGR: intrauterine growth restriction, MD: mean difference, and RR: risk ratio. Bold results represent significant effects.

## Data Availability

No new data were created or analyzed in this study. Data sharing is not applicable to this article, as it is based on a systematic review and meta-analysis of previously published studies. For further details, refer to the MDPI Research Data Policies at https://www.mdpi.com/ethics, accessed on 3 December 2024.

## Supplementary Materials

The following supporting information can be downloaded at: https://www.mdpi.com/article/10.3390/jcm14072134/s1, Figure S1: PRISMA Flowchart of article selection; Figure S2: Forest plot of the outcome preeclampsia regardless of aspirin initiation time; Figure S3: Forest plot of the outcome intrauterine growth restriction below the 10 percentile regardless of aspirin initiation time; Figure S4: Forest plot of the outcome intrauterine growth restriction below the 5 percentile regardless of aspirin initiation time; Figure S5: Forest plot of the outcome intrauterine growth restriction below the 3 percentile regardless of aspirin initiation time; Figure S6: Forest plot of the outcome preterm birth before week 37 regardless of aspirin initiation time; Figure S7: Forest plot of the outcome gestational age at delivery (in weeks) regardless of aspirin initiation time; Figure S8: Forest plot of the outcome actual birth weight (in grams) regardless of aspirin initiation time; Figure S9: Forest plot of the outcome placental abruption regardless of aspirin initiation time; Figure S10: Forest plot of the outcome neonatal intensive care unit admission regardless of aspirin initiation time; Figure S11: Forest plot of the outcome perinatal death regardless of aspirin initiation time; Figure S12: Forest plot of the outcome postpartum hemorrhage regardless of aspirin initiation time; Figure S13: Forest plot of the outcome preeclampsia with early initiated (<week 20) aspirin; Figure S14: Forest plot of the outcome intrauterine growth restriction below the 10 percentile with early initiated (<week 20) aspirin; Figure S15: Forest plot of the outcome intrauterine growth restriction below the 5 percentile with early initiated (<week 20) aspirin; Figure S16: Forest plot of the outcome intrauterine growth restriction below the 3 percentile regardless of aspirin initiation time; Figure S17: Forest plot of the outcome preterm birth before week 37 with early initiated (<week 20) aspirin; Figure S18: Forest plot of the outcome gestational age at delivery (in weeks) with early initiated (<week 20) aspirin; Figure S19: Forest plot of the outcome actual birth weight (in grams) with early initiated (<week 20) aspirin; Figure S20: Forest plot of the outcome preeclampsia with late initiated (>week 20) aspirin; Figure S21: Forest plot of the outcome intrauterine growth restriction below the 10 percentile with late initiated (>week 20) aspirin; Figure S22: Forest plot of the outcome preterm birth before week 37 with late initiated (>week 20) aspirin; Figure S23: Forest plot of the outcome gestational age at delivery (in weeks) with late initiated (>week 20) aspirin; Figure S24: Forest plot of the outcome actual birth weight (in grams) with late initiated (>week 20) aspirin; Figure S25: Forest plot of the outcome perinatal death with late initiated (>week 20) aspirin; Table S1: PRISMA 2020 checklist; Table S2: Detailed search strategy; Table S3: Eligibility criteria in each included article; Table S4: Outcomes and outcome definitions in the included studies; Table S5: Risk of bias assessment of each individual study and outcome; Table S6: Certainty of evidence using the GRADEPro tool.
