# Supplementary material for: Optimal Aspirin Dosage for the Prevention of Preeclampsia and Other Adverse Pregnancy Outcomes: A Systematic Review and Meta-Analysis of Randomized Controlled Trials
Source: J Clin Med. 2025 Mar 21;14(7):2134. doi: 10.3390/jcm14072134 (PMC11989913; doi:10.3390/jcm14072134)

Supplementary Material

TABLE OF CONTENT

Supplementary Table S1. PRISMA 2020 checklist

Supplementary Table S2. Detailed search strategy

Supplementary Table S3. Eligibility criteria in each included article

Supplementary Table S4. Outcomes and outcome definitions in the included studies

Supplementary Table S5. Risk of bias assessment of each individual study and outcome

Supplementary Table S6. Certainty of evidence using the GRADEPro tool

Supplementary Figure S1. PRISMA Flowchart of article selection

Supplementary Figure S2. Forest plot of the outcome **preeclampsia** regardless of aspirin initiation time

Supplementary Figure S3. Forest plot of the outcome **intrauterine growth restriction below the 10 percentile** regardless of aspirin initiation time

Supplementary Figure S4. Forest plot of the outcome **intrauterine growth restriction below the 5 percentile** regardless of aspirin initiation time

Supplementary Figure S5. Forest plot of the outcome **intrauterine growth restriction below the 3 percentile** regardless of aspirin initiation time

Supplementary Figure S6. Forest plot of the outcome **preterm birth before week 37** regardless of aspirin initiation time

Supplementary Figure S7. Forest plot of the outcome **gestational age at delivery (in weeks)** regardless of aspirin initiation time

Supplementary Figure S8. Forest plot of the outcome **actual birth weight (in grams)** regardless of aspirin initiation time

Supplementary Figure S9. Forest plot of the outcome **placental abruption** regardless of aspirin initiation time

Supplementary Figure S10. Forest plot of the outcome **neonatal intensive care unit admission** regardless of aspirin initiation time

Supplementary Figure S11. Forest plot of the outcome **perinatal death** regardless of aspirin initiation time

Supplementary Figure S12. Forest plot of the outcome **postpartum hemorrhage** regardless of aspirin initiation time

Supplementary Figure S13. Forest plot of the outcome **preeclampsia** with early initiated (<week 20) aspirin

Supplementary Figure S14. Forest plot of the outcome **intrauterine growth restriction below the 10 percentile** with early initiated (<week 20) aspirin

Supplementary Figure S15. Forest plot of the outcome **intrauterine growth restriction below the 5 percentile** with early initiated (<week 20) aspirin

Supplementary Figure S16. Forest plot of the outcome **intrauterine growth restriction below the 3 percentile** regardless of aspirin initiation time

Supplementary Figure S17. Forest plot of the outcome **preterm birth before week 37** with early initiated (<week 20) aspirin

Supplementary Figure S18. Forest plot of the outcome **gestational age at delivery (in weeks)** with early initiated (<week 20) aspirin

Supplementary Figure S19. Forest plot of the outcome **actual birth weight (in grams)** with early initiated (<week 20) aspirin

Supplementary Figure S20. Forest plot of the outcome **preeclampsia** with late initiated (>week 20) aspirin

Supplementary Figure S21. Forest plot of the outcome **intrauterine growth restriction below the 10 percentile** with late initiated (>week 20) aspirin

Supplementary Figure S22. Forest plot of the outcome **preterm birth before week 37** with late initiated (>week 20) aspirin

Supplementary Figure S23. Forest plot of the outcome **gestational age at delivery (in weeks)** with late initiated (>week 20) aspirin

Supplementary Figure S24. Forest plot of the outcome **actual birth weight (in grams)** with late initiated (>week 20) aspirin

Supplementary Figure S25. Forest plot of the outcome **perinatal death** with late initiated (>week

20) aspirin

Supplementary Table S1. PRISMA 2020 checklist

Section and topic	Item #	Checklist item	Location (line in text) where item is reported
**Title**			
Title	1	Identify the report as a systematic review.	1-4
**Abstract**			
Abstract	2	See the PRISMA 2020 for Abstracts checklist (table 2).	15-37
**Introduction**			
Rationale	3	Describe the rationale for the review in the context of existing knowledge.	41-76
Objectives	4	Provide an explicit statement of the objective(s) or question(s) the review addresses.	69-76
**Methods**			
Eligibility criteria	5	Specify the inclusion and exclusion criteria for the review and how studies were grouped for the syntheses.	99-108
Information sources	6	Specify all databases, registers, websites, organisations, reference lists and other sources searched or consulted to identify studies. Specify the date when each source was last searched or consulted.	85-86

Search strategy	7	Present the full search strategies for all databases, registers and websites, including any filters and limits used.	85-91
Selection process	8	Specify the methods used to decide whether a study met the inclusion criteria of the review, including how many reviewers screened each record and each report retrieved, whether they worked independently, and if applicable, details of automation tools used in the process.	93-97
Data collection process	9	Specify the methods used to collect data from reports, including how many reviewers collected data from each report, whether they worked independently, any processes for obtaining or confirming data from study investigators, and if applicable, details of automation tools used in the process.	110-113
Data items	10a	List and define all outcomes for which data were sought. Specify whether all results that were compatible with each outcome domain in each study were sought (e.g. for all measures, time points, analyses), and if not, the methods used to decide which results to collect.	115-128
	10b	List and define all other variables for which data were sought (e.g. participant and intervention characteristics, funding sources). Describe any assumptions made about any missing or unclear information.	115-128
Study risk of bias assessment	11	Specify the methods used to assess risk of bias in the included studies, including details of the tool(s) used, how many reviewers assessed each study and whether they worked independently, and if applicable, details of automation tools used in the process.	130-132
Effect measures	12	Specify for each outcome the effect measure(s) (e.g. risk ratio, mean difference) used in the synthesis or presentation of results.	133-146

Synthesis methods	13a	Describe the processes used to decide which studies were eligible for each synthesis (e.g. tabulating the study intervention characteristics and comparing against the planned groups for each synthesis (item #5)).	133-146
	13b	Describe any methods required to prepare the data for presentation or synthesis, such as handling of missing summary statistics, or data conversions.	Not applicable
	13c	Describe any methods used to tabulate or visually display results of individual studies and syntheses.	137-138 and 143
	13d	Describe any methods used to synthesise results and provide a rationale for the choice(s). If meta-analysis was performed, describe the model(s), method(s) to identify the presence and extent of statistical heterogeneity, and software package(s) used.	133-146
	13e	Describe any methods used to explore possible causes of heterogeneity among study results (e.g. subgroup analysis, meta-regression).	143-144
	13f	Describe any sensitivity analyses conducted to assess robustness of the synthesised results.	145-146
Reporting bias assessment	14	Describe any methods used to assess risk of bias due to missing results in a synthesis (arising from reporting biases).	130-132
Certainty assessment	15	Describe any methods used to assess certainty (or confidence) in the body of evidence for an outcome.	148-150
**Results**			

Study selection	16a	Describe the results of the search and selection process, from the number of records identified in the search to the number of studies included in the review, ideally using a flow diagram (see fig 1).	153-155
	16b	Cite studies that might appear to meet the inclusion criteria, but which were excluded, and explain why they were excluded.	166-167
Study characteristics	17	Cite each included study and present its characteristics.	168-172
Risk of bias in studies	18	Present assessments of risk of bias for each included study.	220-224
Results of individual studies	19	For all outcomes, present, for each study: (a) summary statistics for each group (where appropriate) and (b) an effect estimate and its precision (e.g. confidence/credible interval), ideally using structured tables or plots.	174-215
Results of syntheses	20a	For each synthesis, briefly summarise the characteristics and risk of bias among contributing studies.	220-224
	20b	Present results of all statistical syntheses conducted. If meta-analysis was done, present for each the summary estimate and its precision (e.g. confidence/credible interval) and measures of statistical heterogeneity. If comparing groups, describe the direction of the effect.	168-224
	20c	Present results of all investigations of possible causes of heterogeneity among study results.	168-224
	20d	Present results of all sensitivity analyses conducted to assess the robustness of the synthesised results.	217-218
Reporting biases	21	Present assessments of risk of bias due to missing results (arising from reporting biases) for each synthesis assessed.	217-218

Certainty of evidence	22	Present assessments of certainty (or confidence) in the body of evidence for each outcome assessed.	220-224
**Discussion**			
Discussion	23a	Provide a general interpretation of the results in the context of other evidence.	227-260
	23b	Discuss any limitations of the evidence included in the review.	227-260
	23c	Discuss any limitations of the review processes used.	262-281
	23d	Discuss implications of the results for practice, policy, and future research.	282-295
**Other information**			
Registration and protocol	24a	Provide registration information for the review, including register name and registration number, or state that the review was not registered.	80-81
	24b	Indicate where the review protocol can be accessed, or state that a protocol was not prepared.	80-81
	24c	Describe and explain any amendments to information provided at registration or in the protocol.	81-83
Support	25	Describe sources of financial or non-financial support for the review, and the role of the funders or sponsors in the review.	Patents
Competing interests	26	Declare any competing interests of review authors.	Patents

Availability of data, code, and other materials	27	Report which of the following are publicly available and where they can be found: template data collection forms; data extracted from included studies; data used for all analyses; analytic code; any other materials used in the review.	Patents

Supplementary Table S2. Detailed search strategy

Name of Database	Detailed search
PubMed	("Aspirin"[MeSH] OR "Acetylsalicylic Acid"[MeSH] OR ASA) AND ("Pregnancy"[MeSH] OR "Pregnant Women"[MeSH] OR "Gravidity"[MeSH] OR "Preeclampsia"[MeSH] OR "Eclampsia"[MeSH] OR "Premature Birth"[MeSH] OR "Infant, Premature"[MeSH] OR "Preterm Birth"[MeSH] OR "Preterm Labor"[MeSH] OR "Fetal Growth Retardation"[MeSH] OR "Intrauterine Growth Restriction"[MeSH] OR "Small for Gestational Age"[MeSH]) AND (randomized controlled trial[Publication Type] OR random* OR "clinical trial"[MeSH])
Embase	('acetylsalicylic acid'/exp OR 'aspirin'/exp OR ASA) AND ('pregnancy'/exp OR 'pregnant woman'/exp OR 'gravidity'/exp OR 'preeclampsia'/exp OR 'eclampsia'/exp OR 'premature birth'/exp OR 'preterm birth'/exp OR 'preterm labor'/exp OR 'fetal growth retardation'/exp OR 'intrauterine growth restriction'/exp OR 'small for gestational age'/exp) AND ('randomized controlled trial'/exp OR random* OR 'clinical trial'/exp)
Cochrane Library	(aspirin OR "acetylsalicylic acid" OR ASA) AND (pregnancy OR pregnant OR gravidity OR preeclampsia OR eclampsia OR "preterm delivery" OR "preterm birth" OR "premature delivery" OR "premature birth" OR "fetal growth restriction" OR FGR OR "intrauterine growth restriction" OR IUGR OR "small for gestational age" OR SGA) AND random*

Supplementary Table S3. Eligibility criteria in each included article

Author (year)	Inclusion criteria ("verbatim")	Exclusion criteria ("verbatim")
Abdali_2013	These risks include personal or family history of hypertension, gestational hypertension and preeclampsia, personal history of spontaneous abortion, obesity, middle age and young age (fewer than 18 and above 35 years)	Exclusion criteria in this study included any need to steroidal, non-steroidal and anti-hypertensive drugs, multiple pregnancy, cardiovascular disease, chronic gastrointestinal disease, asthma, bleeding disease, diabetes or any endocrine disorder such as hyperthyroidism or intolerance to the circadian blood pressure monitoring device.
Abdi_2020	Pregnant women with gestational age of 12 to 15 weeks and a history of PE in previous pregnancies (at least one previous pregnancy)	Multiple gestations, gestational diabetes mellitus, chronic medical diseases (eg, hypertension or diabetes mellitus), smoking, coagulation disorders, abnormal uterine artery Doppler at ultrasound screening,19 abnormal serum level of pregnancy-associated plasma protein A (PAPP-A) at the first-trimester screening,20 allergy to aspirin, and

		unwillingness to participate in the research
Andrade_2021	Singleton pregnancy, nulli- parity, gestational age between 11 and 13weeks and 6days	Hyper- tensive disorders with vasculopathies, thrombophilia, smokers, allergic to ASA, and those who did not participate in the study
Ayala_2012	Pregnant patients receiving medical care and follow-up at a high-risk unit due to familial or personal history of either gestational hypertension or preeclampsia; chronic hypertension; cardiovascular, endocrine, bleeding, or metabolic disease; personal history of spontaneous abortion; multiple pregnancy; obesity; and adolescent or middle- aged nulliparous pregnancy (<18 or >35 yrs); in addition: gestational age ≤16 wks at randomization and maternal age ≥18 yrs	Multiple pregnancy, chronic hyperten- sion or any other condition requiring the use of BP-lowering medication, cardiovascular disorders (unstable angina pectoris, heart failure, life-threatening arrhythmia, atrial fibrillation, kidney failure, and grade III–IV retinopathy), chronic liver disease, any disease requiring the use of anti-inflammatory medication, diabetes or any other endocrine disease such as hyperthyroidism, history of drug/alcohol abuse, night/ shiftwork employment, acquired immunodeficiency syn- drome (AIDS), intolerance to ABPM, and inability to

		communicate and comply with all of the study requirements
Benigni_1989	High risk for preeclampsia: chronic hypertension, previous pregnancy was complicated with fetal demise due to placental insufficiency, severe intrauterine growth retardation or early onset of preeclampsia (<32th week)	Patients with antiphospholipid antibodies.
Beroyz_1994	Women in the 12 and 32 weeks of gestation at risk for preeclampsia or IUGR for the use of aspirin to be contemplated, but without clear indications for or against its use. Prophylactic entry: Women with a history of pre-eclampsia or IUGR in a previous pregnancy, chronic hypertension, renal disease, or other risk factors, such as maternal age, family history, or multiplepregnancy. Therapeutic entry: Women with signs or symptoms of pre- eclampsia or IUGR in the current pregnancy.	Women with increased risk of bleeding, asthma, allergy to aspirin, or high likelihood to immediate delivery.

Blomqvist_2018	Three or more consecutive miscarriages in the first trimester (including gestational length of 12 weeks and 6 days) in the same relationship, were eligible. Further inclusion criteria were a negative workup, a body mass index < 35 kg/m2, age < 40 years at the start of the investigation, and no allergy against ASA.	Women who had already taken part in the study, had ongoing conflicting medical treatment, or had an in vitro fertilization (IVF) pregnancy for which the reason for IVF was recurrent miscarriage, were excluded.
Byaruhanga_1998	Previous history of pregnancy-induced hyper- tension, preeclampsia, especially that occurring before 32 weeks of gestation, or eclamp- sia; and pre-existing chronic hypertension.	History of hypersensitivity to aspirin; use of non-steroidal anti-inflammatory drugs; history of peptic ulcer disease; bleeding disorders;<br>history of chronic pulmonary disease; or development of pre-eclampsia prior to entry of trial.
Caritis_1998	One of four high-risk groups: women with pregestational, insulin-treated diabetes mellitus, women with chronic hypertension, women with multifetal gestations, and women who had had preeclampsia in a previous pregnancy.	Women with multifetal gestations were ineligible for the study if they also had diabetes mellitus, chronic hypertension, or proteinuria as defined above, as were women with a history of preeclampsia and current proteinuria.

Caspi_1994	All pregnant women with uncomplicated twin pregnancies	Chronic renal, cardio- vascular, pulmonary or hepatic disorders, past or present coagulopathy or peptic ulcer, gestational diabetes mellitus and known hypersensitivity to aspirin
Chiaffarino_2004	Pregnant women with gestational age at randomisation <14 weeks, who satisfied one or more of the following criteria: Chronic hypertension (diastolic pressure between 90 and 110 mmHg) with or without nephropathy, history of severe pre-eclampsia or eclampsia, history of IUGR (baby below 10th centile), and history of intrauterine foetal death.	Women with a history of chronic disease (except hypertension, renal disease, and chronic diabetes without Hypertension and nephropathy), allergy to aspirin or documented foetal malformations and current twin pregnancy.
Dasari_1998	Primiparous women at 12 weeks gestation were randomly enrolled	Women with one or more complications were excluded from the study, chronic hypertension, any obstructive pulmonary disease, hypersensitivity to aspirin, history of peptic ulcer or hepatic disease, history of long term treatment with nonsteroidal anti-inflammatory drugs (NSAIDS), diabetes mellitus,

		midtrimester abortion and antepartum hemorrhage
Davies_1995	Women with no previous pregnancy proceeding beyond 12 weeks gestation were considered eligible for inclusion in the study if the hemoglobin concentration was greater than 13.3g/dL between 12 and 19 weeks gestation.	Women with multiple pregnancy, diabetes mellitus, recurrent spontaneous abortions, or any contraindication to aspirin therapy were excluded from the study.
Ebrashy_2015	Inclusion were gestational age between 14 and 16 weeks, and a high risk factor for preeclampsia or IUGR, such as previous history of the disease, essential hypertension, positive family history or underlying vascular disorder, maternal age <20 years or >40 years, and gestational diabetes mellitus.	Cases with a known history of salicylate allergy, present or past peptic ul- cer, past use of prostaglandin inhibitors within 10 days before the beginning of the study, as well as cases with other medical disorders such as chronic renal disorders, thyroid diseases, and hepatic and cardiac disorders were excluded from the study.
Gallery_1997	Patients enrolled in this study were women who were considered to be at high risk of developing preeclampsia because of<br>1. Preexisting chronic essential hypertension (BP 2 140/90 mmHg on at	Women with a history of aspirin allergy, aspirin- sensitive asthma, or preexisting bleeding diathesis were excluded from the study, as were women with multiple pregnancy.

	least two occasions in the 12 months prior to pregnancy, or on antihypertensive medication prior to pregnancy, with no history or findings suggestive of renal disease).<br>2. Preexisting renal disease (know nchronic renal disease prior to pregnancy).<br>3. Previous early severe preeclampsia [BP 2 160/110 mmHg + proteinuria (2500/mg/day) + hyperuricaemia (s. uric acid > 0.35 mmol/L), all occurring prior to 34-weeks gestation in a previous pregnancy].	
Golding_1998	All primiparae resident in the parishes of Kingston and St Andrew in Jamaica were eligible.	The key exclusion criterion was that women were not permitted to enter the study after 32 weeks of gestation. However, a small number of women (144 aspirin, 161 placebo) subsequently were found to have an estimated gestation in excess of 32 weeks (after clinical assessment). Their exclusion did

		not alter the findings and they have been retained in the analysis presented here.
Grab_2000	singleton pregnancies of less than 20 gestational weeks with early intrauterine growth restriction, impaired uteroplacental blood, chronic hypertension or history of stillbirth, growth restriction or pre-eclampsia	Patients with diabetes mellitus, pre-existing proteinuric hypertension or fetal malformations or chromosome abnormalities were excluded.
Gu_2020	The inclusion criteria were pregnant women at high-risk for preeclampsia, with one or more high-risk factors or with 2 or more medium-risk factors.<br><br>High-risk factors included history of preeclampsia or gestational hypertension, chronic hypertension, multiple pregnancies, kidney disease, type 1 or type 2 diabetes and autoimmune diseases such as systemic lupus erythematosus and antiphospholipid syndrome [10]. Medium risk factors included primipara, age over 35 years, BMI over	Women who met the following criteria were excluded: not allowed to use aspirin; history of chronic illness (excluding hypertension, kidney disease, chronic diabetes mellitus without hypertension and nephropathy); planned delivery at another hospital; severe fetal malformations; anticoagulation therapy during pregnancy or participant in another study on preeclampsia prevention.

	30 kg/m2, family history of preeclampsia, poor social and economic status, personal history (polycystic ovary syndrome, low birth weight infant or infant younger than gestational age, > 10 years since the previous pregnancy and history of an adverse pregnancy outcome).	
Haapsamo_2010	(i) age <40 years, (ii) <4 previous ovarian stimulations and (iii) no contraindications for aspirin. All the participants were healthy with no pre-existing medical condition.	
Harrington_2000	The included women were otherwise healthy mothers with a normal mix of primigravidae, multigravidae, and different ethnic groups.	Exclusion criteria were: multiple pregnancy, a history of bleeding in the current pregnancy, asthma requiring treatment, known diabetes, chronic hypertension, and known allergy to aspirin.
Hauth_1993	The study population consisted of nulliparous women. Age below 28 years was chosen as an inclusion criterion because women below this age account	Patients with illnesses or conditions known to increase the incidence of preeclampsia or pregnancy-induced hypertension, such as renal disease,

	for 58% of all deliveries in our population and have an incidence of pregnancy-induced hypertension or preeclampsia of 9%, with <1% having chronic hypertensive disease. Older women have more chronic hypertension and less preeclampsia and pregnancy-induced hypertension.	collagen vascular disease, diabetes mellitus, multifetal gestation, and chronic hypertension, were excluded.
Herabutya_1996	All normal nulliparous pregnant women conlined at Ramathibodi Hospital under the age of 30 yearswereselectedafter undergoing routine ultra- sound at 18-22 weeks of gestation to confirm gestational age and to exclude anomalies.	
Hermida_1997	Inclusion criteria for this trial were absence of any condition requiring the use of antihypertensive medication, maternal age (18 to 40 years), and gestational age (<16 weeks).	Exclusion criteria were, among others, multiple pregnancy, chronic hypertension, chronic liver disease, any disease requiring the use of anti-inflammatory medication, diabetes or any other endocrine disease such as hyperthyroidism, as well as intolerance to the use of an ambulatory BP monitor.

Hoffman_2020	We identified nulliparous pregnant women aged between 18–40 years (minors aged ≥14 years were enrolled when permitted by individual ethics boards in the Democratic Republic of the Congo, Kenya, and Zambia), and written informed consent for study participation was obtained from the women, or the parents or guardians of minors, by trained staff. Nulliparous women were selected as they are unlikely to undergo treatment to prevent prematurity because of a lack of obstetrical history and have a tendency to have higher frequencies of preterm birth than multiparous women.17–19 Women were required to have been pregnant for at least 6 weeks and 0 days, and no longer than 13 weeks and 6 days, as confirmed by a study ultrasound.	We excluded women who presented with or had a medical history of: allergy or contra indication to aspirin; previous prescription of aspirin for more than 7 days during the pregnancy; multiple gestations; more than two firsttrimester losses; any other medical condition that might be considered a contraindication to inclusion in the study (eg, diabetes and hypertension).11
Kaandorp_2010	Women between the ages of 18 and 42 years were eligible if they had had	Women who had previous venous or arterial thromboembolism, an indication

	unexplained recurrent miscarriage and were attempting to conceive or were pregnant, with a gestational age of less than 6 weeks.	for anticoagulant treatment during pregnancy, or endocrine disorders (e.g., diabetes mellitus or untreated thyroid dysfunction) were excluded from the study.
Khazardoost_2013	All pregnant women receiving prenatal care at these antenatal clinics were screened for Down syndrome in 15–16 weeks using quadruple test; for patients who did not attend at this period of time, the test was done until 18 weeks of gestation.	Women with alive fetus, AFP ≥2.5 and low risk Down syndrome test result underwent an anomaly scan. Structural or chromosomal abnormality, multiple pregnancy, abortion under 20 weeks of gestation, heparin or other anticoagulant treatment, a history of medical disease and substance abuse were considered as exclusion criteria.
Kyle_1995	Four hundred ninetyfive nulliparous women with no history of hypertension, asthma, or cardiovascular, cerebral, or renal disease were recruited from antenatal clinics at Queen Charlotte's and the John Radcliffe Hospitals before 28 weeks' gestation.<br><br>For the purposes of the trial of	Four hundred ninety-five nulliparous women with no history of hypertension, asthma, or cardiovascular, ce- rebral, or renal disease were recruited from antenatal clinics at Queen Charlotte's and the John Radcliffe Hospitals before 28 weeks' gestation.

	prophylaxis with low-dose aspirin, only those subjects with a positive angiotensin sensitivity test result were asked to participate further.	
Lambers_2009	We included patients who met the following criteria: <39 years of age at the start of treatment, with serum FSH level %10 IU/L on cycle day 3 and with at least one previous IVF or intracytoplasmic sperm injection (ICSI) treatment with failed conception. In the previous treatment cycle, the patients had been given a maximum daily dosage of 225 IU FSH resulting in at least four oocytes at oocyte retrieval. Patients did not have a previous ongoing pregnancy, both ovaries were present, and there was no contraindication for aspirin.	Patients were excluded if they had tubal pathology, ovarian hyperstimulation syndrome in a previous treatment cycle, body mass index >30 kg/m2, smoking habit of more than 5 cigarettes per day, untreated endocrinopathy, systemic dis- ease, hypertension, previous allergic reaction to study medi- cation, or contraindication for pregnancy.
Leslie_1995	Pregnant women attending Royal North Shore Hospital. Sydney, for antenatal care and delivery were eligible for enrolment if they had a singleton	Women with a history of aspirin allergy, aspirin-sensitive asthma or a pre-existing bleeding diathesis were excluded from the study.

	pregnancy and were considered to be at high risk of developing pre-eclampsia because of either pre- existing chronic essential hypertension, pre-existing renal dis- ease or previous severe pre-eclampsia.	
Lin_2021	Inclusion criteria were as follows: (1) age ≥18 and <55 years; (2) singleton pregnancy; (3) live fetus at the gestational age of 12 to 20 weeks; (4) definition of high risk of developing preeclampsia: (a) at least 1 high risk factor, namely, history of preeclampsia, diabetes mellitus (type 1 or 2), or chronic hypertension; or (b) at least 2 of the following intermediate risk factors, including obesity (pre-pregnancy body mass index [pre-BMI] ≥28 kg/m2), advanced maternal age (≥35 years), family history of preeclampsia (mother or/and sister) or nulliparity; (5) ability to undergo all procedures listed in the	The exclusion criteria included (1) allergy to aspirin; (2) asthma; (3) peptic ulcers; (4) no tolerance to this study because of severe heart, liver, or renal disease; (5) autoimmune diseases; (6) mental disorders; (7) history of alcohol or drug abuse within 6 months; (8) in-vitro fertiliza- tion; (9) previous registration in another drug trial within the previous 3 months.

	protocol; (6) a written informed consent for participation in the study.	
Liu_2016	The diagnostic criteria of pregnancy induced hypertension syndrome was according to “2010 NICE guide” (3), by inclu- ding age ≥40, BMI ≥35 kg/m2, preeclampsia family history, multiple pregnancy history, previous hypertensive disease, chronic nephrosis, autoim- mune disease like systemic lupus erythematosus; systemic lupus erythematosus and antiphospholi- pid syndrome, type 1 or type 2 diabetes, chronic hypertension well controlled. Select standard: (1) 50 > Age ≥ 18; (2) Single birth; (3) No abortion history.	Rule out standard: (1) Coagulation di- sorder; 2) Need oral intake of anticoagulants like Warfarin or vein application of heparin; (3) Have hypertension before pregnancy and fail to control up to standard with drug; (4) Being allergic to aspirin, low compliance.
Liu_2017	The inclusion criteria for the study were: i) ≥18 but <50 years of age; ii) in accordance with 2013 standards for hyperten- sion in pregnancy issued by ACOG, but with high risk of pre- eclampsia such as >40 years, obesity, chronic hyperten- sion, chronic kidney	The exclusion criteria were: i) artificial insemina- tion; ii) coagulation disorders; iii) hard to control medication for hypertension in pregnancy; iv) an allergy to aspirin; and v) bad compliance.

	disease, type 1 or 2 diabetes mellitus, family history of pre-eclampsia, previous pregnancy along with pre-eclampsia, thrombosis history, and systemic lupus erythematosus; iii) single birth; and iv) no past history of having aspirin and anticoagulant medications such as warfarin, and no complications such as rheumatic disease and chronic atrial fibrillation that require ingesting aspirin or warfarin.	
Louden_1992	One group included 18 normal primigravidae (mean age 24, SD S years) who were recruited from the antenatal booking clinic at 16 weeks gestation, and these subjects were simply randomized with block size four, to receive either 60 mg aspirin (n=10), or identical placebo (n=8),daily until delivery. Another group included 16 primi- gravidae (mean age 23, S D 4 years) who were recruited after	None of the pregnancies was complicated by essential hyper- tension, renal disease or use of drugs known to influence plate- let behavior. Informed consent was obtained in all cases.

	admission to hospital with a diagnosis of GH	
McCowan_1999	Preg- nant women with singleton pregnancies were eligible for recruitment to the study if they met the following cri- teria: ultrasound evidence suggesting a small for gesta- tional age fetus (abdominal circumference c lo%)? a previous anatomy scan at < 20 weeks to confirm dates and with no evidence of fetal abnormality; a gestational age of between 24 and 36 weeks; an umbilical artery Doppler resistance index (RI) > 95% for gestation17;no previous aspirin use during the pregnancy; no contra- indications to aspirin use; and provided informed con- sent to participate in the study. Two umbilical Doppler results > 95% performed 1 to 2 days apart were necessary before recruitment.	
McParland_1990	Patients underwent doppler ultrasound examination of the uteroplacental circulation as previously described.2,s	Reasons for exclusion from the study included known aspirin allergy, maternal diabetes mellitus, bleeding

	Patients with abnormal waveforms2,12 had repeat examinations at 24 weeks' gestation, and if the waveforms were still abnormal they were invited to take part in the trial.	disorders, peptic ulceration, and systemic lupus erythematosus.
Mone_2018	Nulliparous women over 18 years old between 11 and 13+6 weeks' gestation with a viable singleton pregnancy who did not meet criteria for taking aspirin based upon major pre-eclampsia risk-factors (chronic kidney disease; autoimmune disease, eg, systemic lupus erythematosus, diabetes mellitus and chronic hypertension) were eligible for inclusion and thus were recruited at antenatal booking clinics selected at random.	Exclusion criteria included participants already taking part in a clinical trial, co-existence of a fetal congenital anomaly at recruitment or those with aspirin hypersensitivity.
Morris_1996	flow velocity waveforms were obtained from both uterine arteries in all nulliparous women at the routine l&week fetal morphology scan. If abnormal uteroplacental resistance was demonstrated, one of three clinicians	We excluded from the trial any woman whose ultrasound examination dated the preg- nancy as less than 17 weeks or more than 19 weeks and 6 days.

	(JMM, RAF, or DAE) asked the women to participate in a randomized, double-blind, placebo-controlled trial of low-dose aspi- rin therapy.	
North_1995	Women with glomerulonephritis, reflux nephropathy or diabetic nephropathy who during pregnancy received either no anticoagulant therapy, low dose aspirin alone or heparin with antiplatelet drugs, were included in the study. To be eligible for the study, the renal disease had to be evident before 20 weeks gestation and the pregnancy continue beyond 16 weeks gestation.	Women who received multiple therapeutic regimens, such as renal transplant patients, were excluded.
Obido_2015	inclusion criteria were singleton pregnancy under- going ultrasound examination at 11+0 to 13+6weeks and deemed to be at high risk for pre-eclampsia by the criteria listed: Chronic hypertension, prepregnancy diabetes mellitus, previous preeclampsia, obesity (BMI >30), bilateral uterine artery	We excluded pregnancies with multiple gestation, fetal aneuploidy, major fetal structural anomaly and bleeding disorder, and women with allergy to aspirin or already on aspirin or heparin.

	notches preeclampsia risk score greater than 6, low PAPP-A (< 0.52 MoM)	
Pattison_2000	Only women with antiphospholipid syndrome were included. All women had a history of ≥3 miscarriages and one or more of the following laboratory findings before pregnancy or early during the index pregnancy: anticardiolipin antibodies (≥5 immunoglobu- lin G phospholipid [GPL] units or ≥5 immunoglobulin M phospholipid [MPL] units) or detection of lupus antico- agulant. The trial was conducted on an intent-to-treat basis, and compliance was neither recorded nor tested for but was strongly encouraged.	Women with a history of thrombosis, systemic lupus erythematosus, or current or planned therapy with corticosteroids, nonsteroidal anti- inflamma- tory drugs, heparin, or marine lipids were excluded.
Rolnik_2017	The eligibility criteria were maternal age ≥ 18 years, no serious mental illness or learning difficulty and singleton pregnancy with live fetus with no major abnormality demonstrated on the 11–13-week scan.	We excluded pregnancies with no follow-up and those ending in termination or miscarriage.

Rotchell_1998	All women between 12 and 32 weeks of ges- tation without contraindications were eligible for entry into the trial.	Contraindications were an increased risk of bleeding, known allergy to aspirin, high likelihood of immediate delivery or previous placental abruption.
Scazoccio_2017	Inclusion criteria were as follows: (1) maternal age ≥18 years; (2) singleton pregnancy; (3) crown–rump length (CRL) of 45–84mm; and (4) mean UtA pulsatility index (PI) > 95th percentile14 .	Exclu- sion criteria were as follows: (1) pre-existing hypertensive, immune, renal or cardiovascular disease; (2) history of PE in previous pregnancy; (3) history of gastric ulcer; (4) known allergy or hypersensitivity to aspirin; (5) hemorrhagic disease; (6) fetal malformation (including chromosomopathy); or (7) active treatment with heparin or aspirin before recruitment.
Schiff_1989	At least one of the following factors:<br>nulliparity<br>twin gestation<br>history of preeclamptic toxaemia	history of chronic (essential) hypertension<br>long term treatment with NSAID, or usage of such drugs during the previous six weeks<br>pregnancy-induced hypertension detected before screening<br>proteinuria detected before screening

		history of obstructive pulmonary disease<br>coagulation disorders<br>heart failure<br>chronic renal or pulmonary disease<br>hepatic or peptic ulcer disease<br>sensitivity to aspirin
Schiff_1990	At least one of the following factors:<br>nulliparity gestational age between 30-36 weeks a diagnosis of mild pregnancy induced hypertension no signs of moderate to severe pregnancy induced hypertension	known sensitivity to aspirin chronic hypertension chronic renal disorder antihypertensive treatment before admission
Schröcksnadel_1992	primiparous women between the 28th and 32nd week of pregnancy with a positive roll over test	already manifest or preexistent hypertension<br>impending premature delivery<br>preexisting disorders of the kidney, liver, stomach, heart, or lungs mental illness substance abuse DM neoplastic illness GI disturbances with impaired medication resorption, abnormal ultrasound findings, body weight over 100kg, medications with anticoagulants or analgesics, known aspirin allergy

Sibai_1993	Nulliparous women from 13 to 25 weeks pregnancy; initial blood pressure below 135/85mmHg; no prteinuria.	Women with chronic hypertension, renal disease, diabetes mellitus, and other medical illnesses were excluded.
Stanescu_2018	Inclusion criterion: singleton pregnancies – 150 patients were screened positive for FGR prediction using the FMF algorithm for first trimester screening.	Exclusion criteria: constitutionally small fetuses, screened posi- tive for infections, fetal chromosomal or structural abnormalities, gestational diabetes or multiple pregnancies.
Subtil_2003	They were eligible if they were nulliparous (no previous delivery at or after 22 weeks), at a gestational age between 14 and 20þ6 weeks, planned to continue prenatal care and give birth in the participating facility and provided written informed con- sent. Inclusion did not require any additional examination (in particular, bleeding time was not tested before or during the treatment).	Exclusion was required if a patient had a known history of hypertension, a potential indication (antiphospholipid antibodies, lupus) for or a contraindication (allergy, fre- quent haematomas or bleeding, history of haemorrhage during surgery, tooth extraction or other, recent gastric or duodenal ulcer, severe asthma) to aspirin or other anticoagu- lant treatment during this pregnancy. Multiple pregnancy was not an exclusion criterion.
Taherian_2002	Nulliparity, single gestation, first prenatal visit before 20 weeks of	A history of cardiovascular, renal or endocrinologic problems, medical or

	gestation, systolic/diastolic blood pressure (BP) lower than 130/80 mmHg, and no proteinuria detectable by a dipstick.	obstetric complications and those with known hazardous condition (multifetal gestation, hydatidiform mole).
Trudinger_1988	The 46 patients included in this trial were those in whom the umbilical artery velocity wave form systolic/diastolic ratio was above the 95th centile of our previously reported nor- mal range.8 The trial was restricted to patients with a singleton pregnancy and gestational age between 28 and 36 weeks. Patients were invited to participate in this trial on the first occasion that the Doppler study result was recognized as abnormal during this period.	Women with severe hypertension (diastolic blood pressure > 110 mm Hg or diastolic blood pressure >90 mm Hg and proteinuria) in whom the maternal condition rather than the fetal compromise might necessitate early delivery were not included in the study.
Tulppala_1997	at least 3 consecutive miscarriages	
Vainio_2002	Women at risk of pre-eclampsia or intrauterine growth retardation were recruited from the population of pregnant women routinely attending antenatal clinics in Tampere and its	The exclusion criteria were gestational weeks < 12 or > 14, asthma, allergy to acetylsalicyclic acid, previous peptic ulcer, or the use of prostaglandin

	envious. Anamnestic risk factors included a history of chronic hypertension, familial risk of pre-eclampsia (mother or sister), gestational diabetes, age < 20 or > 40 years, previous pre-eclampsia, previous intrauterine growth retardation, or previous intrauterine death.	inhibitors within ten days before investigation.
Viinikka_1993	They were regarded to be at increased risk of developing preeclampsia, since they had arterial hypertension (blood pressure without treatment >140/90mmHg already before pregnancy), or had had severe pre-eclampsia in previous pregnancy (Table 1). None of them excreted protein (~30m0g/24 h) in urine before pregnancy.	
Villa_2012	Age under 20 years<br>Age over 40 years<br>Obesity (body mass index over 30 kg/m2)<br>Chronic hypertension (‡140/90 mmHg	The exclusion criteria were allergy to aspirin; tobacco smoking (during this pregnancy); multiple pregnancy; and a history of asthma, peptic ulcer, placental ablation, infammatory bowel diseases

	or medication for hypertension before 20 weeks of gestation)<br>Sjögren's syndrome<br>A history of one of the following conditions:<br>Gestational diabetes<br>Pre-eclampsia (blood pressure ‡140 mmHg systolic or ‡90 mmHg diastolic and proteinuria ‡0.3 g/day or dipstick equivalent in two consecutive measurements) Small for gestational age (birthweight <)2SD)<br>Fetus mortus (fetal death after 22 weeks of gestation or >500 g weight in a previous pregnancy)	(Crohn's disease, colitis ulcerosa), rheumatoid arthritis, haemophilia or thrombophilia (previous venous or pulmonary thrombosis or coagulation abnormality).
Wallenburg_1986	Uncomplicated pregnancy of 26 week's. Sensitivity to intravenously infused angiotensin II	ThNone of the women had a history of hypertension or cardiovascular or renal disease; course of pregnancy in all cases had been uncomplicated, with a maximum diastolic blood pressure of 80mmHg; None had taken drugs except oral iron supplements; and diets were unrestricted

Wang_1996	pregnant women at high risk for IUGR	No liver and hematologic diseases
Yu_2003	Women with a mean PI above 1.6, which was the 95th centile in our previous screening study11, were offered the option of participating in the trial and those agreeing gave written consent.	Exclusion criteria were: pre-existing hypertensive, renal or cardiovascular disease, diabetes mellitus, bleeding disorders, systemic lupus erythematosus, peptic ulceration, hypersensitivity to aspirin, and the finding at the 23-week scan of a fetal abnormality or fetal growth restriction.
Zimmermann_1996	Chronic hypertension<br>Familar risk of pre-eclampsia History of pre-eclampsia<br>History of IUGR<br>History of intrauterine death	

Supplementary Table S4. Outcomes and outcome definitions in the included studies

Author (year)	Outcome	Outcome definition
Abdali_2013	IUGR	no definition given
Abdali_2013	preterm delivery	no definition given
Abdali_2013	preeclampsia	no definition given
Abdi_2020	IUGR	Review of the literature shows that IUGR, as fetal growth below the 10th percentile appropriate for gestational age.
Abdi_2020	preeclampsia	no definition given
Abdi_2020	gestational age	gestational age in weeks
Abdi_2020	birth weight	actual birth weight in gramms
Andrade_2021	preeclampsia	no definition given
Ayala_2012	preeclampsia	Preeclampsia was defined as gestational hypertension (following the criteria given above) and proteinuria, ≥300 mg/24-h urine, diagnosed after the 20th week of gestation in a previously normoten- sive woman.

Ayala_2012	preterm delivery	Preterm delivery was defined as delivery at <37 weeks of gestation
Ayala_2012	IUGR	no definition given
Ayala_2012	stillbirth	no definition given
Ayala_2012	gestational age	gestational age in weeks
Ayala_2012	birth weight	actual birth weight in grms
Benigni_1989	preterm delivery	no definition given
Benigni_1989	perinatal death	fetal death + perinatal death
Beroyz_1994	preeclampsia	The study outcome of proteinuric preeclampsia required the development of hypertension and proteinuria after randomization. For those with baseline diastolic pressure below 90 mm Hg, hypertension was defined as a rise of at least 25 mm Hg, to 90 mm Hg or higher. For those with an initial diastolic pressure of 90 mm Hg or above, an increment of at least 15 mm Hg was required. Proteinuria was defined as the appearance after randomization of at least 1 + on protein stick-testing during

		pregnancy, without evidence of urinary tract infection
Beroyz_1994	preterm delivery	delivery <37 weeks
Beroyz_1994	IUGR	Fetal growth below the 3rd percentile appropriate for gestational age.
Beroyz_1994	perinatal death	Stillbirths included all intrauterine deaths at or after 24 weeks, and neonatal deaths included all deaths after birth up to the age of 28 days. Spontaneous miscarriages and induced abortions before 24 weeks and postneonatal deaths up to age 1 year were also recorded. All losses after randomisation up to age 1 year are referred to as total mortality
Beroyz_1994	gestational age	gestational age in weeks
Beroyz_1994	birth weight	actual birth weight in grams
Beroyz_1994	placental abruption	no definition given
Beroyz_1994	NIC admission	no definition given
Blomqvist_2018	NIC admission	no definition given
Blomqvist_2018	preeclampsia	no definition given

Blomqvist_2018	preterm delivery	delivery before 37 gestational weeks
Blomqvist_2018	perinatal death	intrauterine death
Blomqvist_2018	NIC admission	no definition given
Byaruhanga_1998	preeclampsia	hypertension with proteinuria developing after 20 weeks of gestation in the antepartum, intrapartum, or post-partum period in a previously normotensive woman 16 . w x Significant proteinuria was defined using dipsticks as greater thanq1, or presence of 300 mg of protein or more per litre
Byaruhanga_1998	preterm delivery	delivery before 37 weeks of estimated gestation
Byaruhanga_1998	gestational age	gestational age in weeks
Byaruhanga_1998	birth weight	actual birth weight in grams
Byaruhanga_1998	IUGR	Fetal growth below the 10th percentile appropriate for gestational age.
Byaruhanga_1998	placental abruption	no definition given
Byaruhanga_1998	perinatal death	no definition given
Byaruhanga_1998	NIC admission	no definition given

Caritis_1998	preeclampsia	Preeclampsia, defined in the women who did not have hypertension or proteinuria at base line as the development of hypertension plus one of the following: proteinuria, thrombocytopenia, or pulmonary edema. Hypertension was defined as either a systolic blood pressure 140 mm Hg or a diastolic blood pressure 90 mm Hg on two occasions at least four hours apart. Proteinuria was defined as excretion of 300 mg of protein in a 24-hour urine collection, or two dipstick-test results of 2(100 mg per deciliter), the values recorded at least 4 hours apart, with no evidence of urinary tract infection. Thrombocytopenia was defined as a platelet count of less than 100,000 per cubic millimeter. In women who had normal blood pressure but proteinuria at base line, the diagnosis of preeclampsia required the presence of thrombocytopenia, a serum aspartate aminotransferase concentration of 70 U

		per liter, or hypertension accompanied by either severe headaches, epigastric pain, or a sudden increase in proteinuria (either five times the base-line value or twice base line if the base-line value exceeded 5 g per 24 hours). In the women who had hypertension but no proteinuria at base line, a diagnosis of preeclampsia required the development of proteinuria or thrombocytopenia. In the women who had both hypertension and proteinuria at base line, the diagnosis of preeclampsia required any one of the following: thrombocytopenia, an elevated serum concentration of aspartate aminotransferase (70 U per liter), or worsening hypertension (as shown by two diastolic readings 110 mm Hg taken four hours apart in the week before delivery) combined with either exacerbation of proteinuria (see above), severe headaches, or epigastric pain. A woman was deemed to have

		preeclampsia if she had an eclamptic convulsion or the HELLP syndrome, defined as hemolysis (serum total bilirubin concentration, 1.2 mg per deciliter [20 mmol per liter], a serum lactate dehydrogenase concentration of 600 U per liter, or hemolytic anemia as determined by peripheral smear), elevated serum concentration of aspartate aminotransferase (70 U per liter), and thrombocytopenia
Caritis_1998	placental abruption	Abruptio placentae was diagnosed according to clinical criteria (vaginal bleeding and uterine tenderness) and examination of the placenta
Caritis_1998	preterm delivery	Delivery before the completion of 37 weeks' gestation
Caritis_1998	IUGR	Fetal growth below the 10th percentile appropriate for gestational age.
Caritis_1998	perinatal death	no definition given
Caspi_1994	preterm delivery	delivery before 37-wk gestation

Caspi_1994	IUGR	Fetal growth below the 10th percentile appropriate for gestational age.
Caspi_1994	birth weight	actual birth weight separately to the first and second twin as well
Caspi_1994	gestational age	gestational age in weeks
Caspi_1994	perinatal death	no definition given
Caspi_1994	placental abruption	no definition given
Caspi_1994	preeclampsia	PIH was diagnosed in cases with sustained elevation of more than 15 mm Hg of diastolic pressure or 30 mm Hg of systolic pressure (on at least two occasions 6 or more h apart) compared to levels in the second trimester before treatment. In addition, the presence of proteinuria of more than 300 mg per day, (in the absence of urinary tract infection associated with hypertension) was defined as preeclampsia.
Chiaffarino_2004	gestational age	gestational age in weeks
Chiaffarino_2004	birth weight	actual birth weight in grams
Dasari_1998	gestational age	gestational age in weeks

Dasari_1998	birth weight	actual birth weight in grams
Davies_1995	preeclampsia	Gestational hypertension was defined as 2 diastolic blood pressure readings of greater than 90 mm Hg at least 6 h apart or a single reading of greater than 110 mm Hg. Proteinuria was defined as + + on reagent strip testing or greater than 300 mg/ L in a 24-h urine collection
Davies_1995	NIC admission	no definition given
Davies_1995	perinatal death	no definition given
Ebrashy_2015	preeclampsia	Preeclampsia was defined as development of hypertension (140/90 mm Hg or more) plus proteinuria (>300 mg protein in 24- hour urine sample). Severity was diagnosed when systolic blood pressure reached 160 mm Hg, diastolic blood pressure reached 110 mm Hg, proteinuria reached 2 g in 24 hours urine sample, urine output was

Ebrashy_2015	IUGR	Fetal growth below the 10th percentile appropriate for gestational age.
Ebrashy_2015	preterm delivery	no definition given
Ebrashy_2015	birth weight	actual birth weight in grams
Gallery_1997	gestational age	gestational age in weeks
Gallery_1997	birth weight	actual birth weight in grams
Gallery_1997	preterm delivery	Delivery at <37 wks of gestation
Gallery_1997	perinatal death	intrauterine + neonatal death
Golding_1998	preeclampsia	Development of hypertension using any of the definitions: diastolic blood pressure 2 90 mmHg; systolic blood pressure 2 140 mmHg; rise of 25 mmHg in diastolic blood pressure; rise of 40 mmHg in systolic blood pressure. Proteinuric preeclampsia defined as any hypertension as defined in (1) with proteinuria of 2 l+.
Golding_1998	preterm delivery	Delivery at <37 wks of gestation
Golding_1998	birth weight	actual birth weight in grams
Golding_1998	perinatal death	Excludes miscarriages and terminations < 24 weeks

Golding_1998	NIC admission	no definition given
Grab_2000	preeclampsia	proteinuric hypertension
Grab_2000	gestational age	gestational age in weeks
Grab_2000	birth weight	actual birth weight in grams
Gu_2020	preeclampsia	y the combination of a high blood pressure (the systolic blood pressure should be >140 mmHg and/or the diastolic blood pressure should be >90 mmHg on at least two occasions four hours apart developing after 20 weeks of gestation in previously normotensive women) and proteinuria (>300 mg in 24 h or two readings of at least ++ on dipstick analysis of midstream or catheter urine specimens if no 24 -h collection is available). Women with gestational hypertension in the absence of proteinuria are diagnosed with preeclampsia if they present with any of the following severe features: thrombocytopenia, impaired liver function as indicated by abnormally

		elevated blood concentrations of liver enzymes, severe persistent right upper quadrant or epigastric pain and not accounted for by alternative diagnoses, renal insufficiency, pulmonary edema or new-onset headache unresponsive to acetaminophen and not accounted for by alternative diagnoses or visual disturbances. As for the definition of preeclampsia,there is a more general consensus that early-onset would be labeled when preeclampsia presents before 34 weeks
Gu_2020	IUGR	no definition given
Gu_2020	preterm delivery	Delivery at <34 wks of gestation
Gu_2020	placental abruption	no definition given
Haapsamo_2010	preeclampsia	PIH and pre-eclampsia were classified according to ACOG criteria (ACOG, 2002).
Haapsamo_2010	IUGR	Fetal growth below the 5th percentile appropriate for gestational age in singleton pregnancies.

Haapsamo_2010	gestational age	gestational age in weeks
Haapsamo_2010	birth weight	actual birth weight in grams
Harrington_2000	preeclampsia	The development of pre-eclampsia was based on the definitions given by the CLASP2 study: for those with baseline diastolic pressure below 90 mmHg, hypertension was defined as a rise of at least 25 mmHg, to 90 mmHg or higher, while for those with an initial diastolic pressure of 90 mmHg or above, an increment of at least 15 mmHg was required, proteinuria was defined as the appearance of at least $1 on protein stick-testing during pregnancy without evidence of urinary tract infection
Harrington_2000	IUGR	Fetal growth below the 3rd and 10th percentile separately appropriate for gestational age in singleton pregnancies..
Harrington_2000	placental abruption	The diagnosis of placental abruption was made when there was a clinical diagnosis of antepartum hemorrhage and abdominal pain associated with

		retroplacental clot. Postpartum hemorrhage (PPH) was defined as blood loss from the lower genital tract, after the second stage of labor up to the 24 h post-delivery, in excess of 500 ml as assessed by the midwife/doctor.
Harrington_2000	NIC admission	no definition given
Harrington_2000	perinatal death	stillbirth/neonatal death
Harrington_2000	preterm delivery	Premature delivery was defined as delivery, spontaneous or by intervention, before 37 completed weeks of pregnancy
Hauth_1993	preeclampsia	Preeclamspia: mild, a diastolic blood pressure ~ 90 but < 110 mm Hg on at least two occasions at least 1 hour apart and before or during labor or within 12 hours postpartum and proteinuria of ~ 1 + on two or more occasions at least 1 hour apart in the absence of a urinary tract infection or gross hematuria or ~ 0.5 gm per 24 hours; severe, a diastolic blood pressure ~ 110 mm Hg on at least two occasions at least 1 hour apart before or

		during labor or within 12 hours of delivery and proteinuria as defined for mild preeclampsia. One of the following was also present: headaches or visual disturbance, epigastric pain, oligura, thrombocytopenia, increased bilirubin, increased aspartate aminotransferase, fetal growth retardation, or proteinuria ~ 3 to 4(+) or 4g per 24 hours.
Hauth_1993	preterm delivery	no definition given
Hauth_1993	perinatal death	fetal + neonatal death
Hauth_1993	IUGR	Fetal growth below the 10th percentile separately appropriate for gestational age in singleton pregnancies.
Hauth_1993	birth weight	actual birth weight in gramms
Herabutya_1996	preeclampsia	no definition given
Hermida_1997	preeclampsia	(here defined as gestational hypertension and proteinuria, above 300 mg/24 h, with or without edema)
Hermida_1997	IUGR	no definition given
Hermida_1997	preterm delivery	delivery before 37 weeks of gestation

Hermida_1997	birth weight	actual birth weight in grams
Hermida_1997	gestational age	gestational age in weeks
Hoffman_2020	preterm delivery	deliveries at or after 20 weeks and 0 days of gestation, and before 37 weeks and 0 days of gestation.
Hoffman_2020	preeclampsia	no definition given
Hoffman_2020	IUGR	birthweight lower than 2500 g and birthweight lower than 1500 g
Hoffman_2020	perinatal death	stillbirths and deaths in the perinatal period of 20 weeks' gestation to 7 days postpartum
Kaandorp_2010	preeclampsia	no definition given
Kaandorp_2010	placental abruption	no definition given
Kaandorp_2010	perinatal death	intrauterine fetal death (fetal death after 20 weeks of gestation)
Kaandorp_2010	IUGR	Fetal growth below the 10th percentile separately appropriate for gestational age in singleton pregnancies.
Kaandorp_2010	preterm delivery	Premature delivery was classified a priori in three subgroups according to weeks of

		gestational age (24 to <28, 28 to <32, and 32 to <37).
Kaandorp_2010	gestational age	gestational age in weeks
Khazardoost_2013	preterm delivery	delivery before 37 weeks of gestation
Khazardoost_2013	preeclampsia	blood pressure 140/90 mm Hg and 4 hour apart with proteinuria 300 mg/ 24 h
Khazardoost_2013	IUGR	birth weight less than 10th percentile for gestational age
Khazardoost_2013	perinatal death	fetal loss - abortion <24weeks + neonatal death
Khazardoost_2013	NIC admission	no definition given
Kyle_1995	preeclampsia	Preeclampsia was defined before the start of the trial as an increase in diastolic pressure from the initial reading in the first half of pregnancy by z 25 mmHg to a maximum of 2 90 mm Hg, a definition that selects a high proportion of primgavid women, who are characteristically more susceptible to the disqrder (Oxford definition).", I2 Proteinuric preeclampsia was distinguished by the new

		occurrence of at least " + " of noninfective proteinuria on two or more successive urine samples or >0.3 g per 24-hour urine collection. This is the only definition that has been developed from an analysis of a large database (16,000 pregnancies) and validated on a database of similar size. For comparison of outcome, two other definitions of preeclampsia were used. The International Society for the Study of Hypertension in Pregnancy defines gestational hypertension as a diastolic pressure > 110 mmHg or P 90 mm Hg on two or more occasions z 4 hours apart. Preeclampsia is reserved for gestational hypertension and proteinuria greater or equal to "+" by dipstick analysis or 0.3 gm per 24 hours.'3 The National High Blood Pressure Education Program working party defines pregnancy-induced hypertension as a rise of

		systolic or diastolic pressure of 230 or 15 mmHg, respectively, when the first blood pressure is taken before 20 weeks' gestation. Preeclampsia is defined when there are other systemic signs of disease or the presence of proteinuria (" + " by dipstick or 2 0.3 gm per 24 hours). Transient hypertension is a retrospective diagnosis of nonsustained hypertension with no other features of disease."
Kyle_1995	birth weight	actual birth weight in gramms
Kyle_1995	gestational age	gestational age in days
Kyle_1995	perinatal death	Fetal or neonatal death
Lambers_2009	preeclampsia	n. PE was defined as PIH and proteinuria (300 mg/24 h). In case of haemolysis, elevated liver enzymes and low platelets patients were diagnosed with HELLP syndrome
Lambers_2009	preterm delivery	no definition given

Lambers_2009	IUGR	Small for gestational age (SGA) was defined as birthweight below the 5th percentile.
Leslie_1995	gestational age	gestational age in weeks
Leslie_1995	birth weight	actual birth weight in gramms
Leslie_1995	perinatal death	stillbirths + neonatal death
Leslie_1995	NIC admission	no definition given
Lin_2021	preeclampsia	Preeclampsia was defined according to the American College of Obstetricians and Gynecologists (ACOG) Practice Bulletin, number 222.19 Preeclampsia was diagnosed with SBP 140 mm Hg or DBP 90 mm Hg on at least 2 occasions 4 hours apart, developing after 20 weeks of gestation with previously normal blood pressure (SBP<140 mm Hg and DBP <90 mm Hg), and accompanied by proteinuria. Any of the following criteria were used for diagnoses of proteinuria: protein in urine is 300 mg per 24 hours of urine collection (or can be extrapolated from a timed collection), protein to

		creatinine ratio is 0.3 mg/dL, dipstick reading 2þ (used only if other quantitative methods are not available), and new-onset vital organ damage.
Lin_2021	placental abruption	no definition given
Lin_2021	gestational age	gestational age in weeks
Lin_2021	birth weight	actual birth weight in grams
Lin_2021	preterm delivery	delivery at or after 28 weeks of gestation and before 37þ0 weeks of gestation
Lin_2021	IUGR	birth weight less than 10th percentile for gestational age
Lin_2021	NIC admission	no definition given
Lin_2021	perinatal death	excludes miscarriage, stillbirth, or neonatal death; fetal death with preeclampsia
Liu_2016	preeclampsia	no def. given
Liu_2016	gestational age	gestational age in weeks
Liu_2017	preeclampsia	no definition given
Liu_2017	gestational age	gestational age in weeks
Liu_2017	placental abruption	no definition given

Liu_2017	perinatal death	no definition given
Louden_1992	gestational age	gestational age in weeks
Louden_1992	birth weight	actual birth weight in grams
Louden_1992	IUGR	birth weight less than 10th percentile for gestational age
McCowan_1999	preeclampsia	Pre-eclampsia was defined as gestational hypertension and proteinuria of > 300 mg/24 h and/or at least '++' proteinuria on repeated testing with urine dipsticks in the absence of urinary tract infection
McCowan_1999	IUGR	birth weight less than 10th percentile for gestational age
McCowan_1999	gestational age	gestational age in weeks
McCowan_1999	birth weight	actual birth weight in grams
McCowan_1999	NIC admission	no definition given
McCowan_1999	perinatal death	no definition given
McParland_1990	preeclampsia	proteinuric hypertension
McParland_1990	gestational age	gestational age in weeks
McParland_1990	birth weight	actual birth weight in grams

McParland_1990	perinatal death	intrauterine death, stillbirth, neonatal death
Mone_2018	NIC admission	no definition given
Mone_2018	perinatal death	(stillbirth, neonatal or infant death
Mone_2018	preterm delivery	prior to 34 weeks
Mone_2018	preeclampsia	Pre-eclampsia was defined based on the definition from the International Society for the Study of Hypertension in Pregnancy, with new-onset hypertension (>140 mm Hg systolic or >90 mm Hg diastolic) after 20 weeks' gestation associated with: (1) proteinuria of at least 1 g/L (2+) on urine dipstick testing, (2) maternal organ dysfunction and/or (3) fetal growth restriction (FGR).
Morris_1996	preeclampsia	Preeclampsia was defined as pregnancy-induced hypertension plus proteinuria (l+ or more on dipstick testing on at least two occasions 6 hours apart) or hyperuricemia'
Morris_1996	preterm delivery	Preterm delivery was defined as delivery before 37 completed weeks' gestation.

Morris_1996	birth weight	actual birth weight in gramms
Morris_1996	IUGR	birth weight less than 10th percentile for gestational age
Obido_2015	preeclampsia	early pre-eclampsia (delivery < 34 weeks); severe pre-eclampsia (blood pressure > 160/110 or symptoms including persistent headaches, visual disturbance, or evidence of abnormal renal failure, abnormal liver enzymes or thrombocytopenia)
Obido_2015	IUGR	birth weight less than 10th percentile for gestational age
Pattison_2000	birth weight	actual birth weight in grams
Pattison_2000	preterm delivery	deilvery between weeks 24-37
Pattison_2000	IUGR	birth weight less than 5th percentile for gestational age
Pattison_2000	NIC admission	no definition given
Rolnik_2017	preeclampsia	as proteinuria ≥ 300 mg in 24 h or two readings of at least ++ on dipstick analysis of midstream or catheter urine specimens if no 24-h collection was

		available. PE superimposed on chronic hypertension was defined as significant proteinuria (as defined above) developing after 20 weeks of gestation in women with known chronic hypertension (history of hypertension before conception or presence of hypertension at booking visit before 20 weeks' gestation in the absence of trophoblastic disease
Rolnik_2017	IUGR	no definition given
Rolnik_2017	placental abruption	no definition given
Rolnik_2017	perinatal death	no definition given
Rolnik_2017	NIC admission	no definition given
Rolnik_2017	preterm delivery	no definition given
Rotchell_1998	preeclampsia	proteinuric pre-eclampsia: blood pressure changes as above, with more than trace proteinuria
Rotchell_1998	gestational age	gestational age in weeks
Rotchell_1998	birth weight	actual birth weight in grams
Rotchell_1998	placental abruption	no definition given

Rotchell_1998	perinatal death	stillbirth + neonatal death + infant death
Rotchell_1998	NIC admission	no definition given
Scazoccio_2017	preeclampsia	(systolic BP ≥ 140 mmHg or diastolic BP ≥ 90 mmHg on two readings at least 4h apart in previously normotensive women after 20 weeks of gestation, and proteinuria > 300 mg/24 h)
Scazoccio_2017	gestational age	gestational age in weeks
Scazoccio_2017	birth weight	actual birth weight in grams
Scazoccio_2017	IUGR	) FGR and small-for-gestational age, defined as birth weight < 3rd and < 10th customized percentiles
Scazoccio_2017	NIC admission	no definition given
Schiff_1989	preeclampsia	PE (systolic BP ≥ 140 mmHg or diastolic BP ≥ 90 mmHg on two readings at least 4 h apart in previously normotensive women after 20 weeks of gestation, and proteinuria > 300 mg/24 h)18; (2) early-onset PE, which required delivery before 34 weeks of gestation; (3) severe PE, defined as a BP ≥ 160/110 mmHg on two or more occasions, proteinuria ≥ 5 g/24 h,

		or the presence of maternal complications including: (i) eclampsia; (ii) hemolysis, elevated liver enzymes, low platelet count (HELLP) syndrome (lactate dehydrogenase > 600 IU/L, aspartate transaminase > 62 IU/L, platelet count < 100 × 109/L); (iii) acute renal failure (creatinine > 1.2 mg/dL); (iv) subcapsular hepatic hematoma; (v) pulmonary edema (dyspnea, low oxygen saturation and compatible chest X-ray findings); (vi) placental abruption; or (vii) the presence of disseminated intravascular disease;
Schiff_1989	gestational age	gestational age in weeks
Schiff_1989	birth weight	actual birth weight in grams
Schiff_1989	IUGR	birth weight less than 10th percentile for gestational age
Schiff_1989	NIC admission	no definition given
Schiff_1989	perinatal death	stillbirth + neonatal death
Schiff_1989	preterm delivery	deilvery before week 37

Schiff_1990	preeclampsia	hypertension accompanied by proteinuria (>1g/24h)
Schiff_1990	gestational age	gestational age in weeks
Schiff_1990	birth weight	actual birth weight in grams
Schröcksnadel_1992	preeclampsia	no definition given
Schröcksnadel_1992	gestational age	gestational age in weeks
Schröcksnadel_1992	birth weight	actual birth weight in grams
Schröcksnadel_1992	NIC admission	no definition given
Schröcksnadel_1992	perinatal death	intrauterine + postnatal death
Schröcksnadel_1992	preterm delivery	delivery <37 wk
Schröcksnadel_1992	IUGR	birth weight less than 10th percentile for gestational age
Sibai_1993	preeclampsia	Hypertension + proteinuria (either >= 300 mg/24h or 2 positive dipstick on two occasions four or more hours apart)
Sibai_1993	gestational age	gestational age in weeks
Sibai_1993	birth weight	actual birth weight in grams
Sibai_1993	NIC admission	no definition given
Sibai_1993	perinatal death	fetal and neonatal death

Sibai_1993	IUGR	birth weight less than 10th percentile for gestational age
Stanescu_2018	preeclampsia	no definition given
Stanescu_2018	IUGR	birth weight less than 10th percentile for gestational age
Subtil_2003	preeclampsia	Preeclampsia was diagnosed when pregnancy-related hypertension occurred together with permanent proteinuria, defined as at least two pluses (þþ) on the reagent strip or at least 0.5 g/L in a urine test18 (unless measured during a urinary tract infection). The pre-eclampsia was considered severe if at least two of the following five criteria were present: systolic blood pressure 160 mmHg or diastolic blood pressure 110 mmHg, proteinuria 5 g daily or 3þ on the reagent strip, diuresis 400 mL/day or 20 mL/hour for least two consecutive hours, clinical visual disturbances or abnormal findings at an eye examination, epigastric pain,

		pulmonary oedema, or eclamptic convulsions.
Subtil_2003	placental abruption	either clinical or detected in a pathology examination of the placenta
Subtil_2003	IUGR	birth weight less than 3rd and 10th percentile for gestational age
Subtil_2003	perinatal death	death between 22 weeks of gestation and 7 days after birth, excluding medically indicated terminations of pregnancy
Subtil_2003	gestational age	gestational age in weeks
Subtil_2003	birth weight	actual birth weight in grams
Subtil_2003	NIC admission	no definition given
Taherian_2002	preeclamspia	Patients were considered to have mild preeclampsia if they demonstrated an increase of 30 mmHg in systolic or 15 mmHg in diastolic BP above the standard pressure. In addition, they should have demonstrated equal or greater than 300 mg/24 hours in urine collection, or in two random urine specimens obtained 4 hours apart and containing at least 1+ protein by the dipstick method. Severe

		preeclampsia was defined as BP equal or greater than 160/110 mmHg and 4+ protein by dipstick on two occasions 4 hours apart, according to the American College of Obstetricians and Gynecologists' Bulletin.
Taherian_2002	birth weight	actual birth weight in gramms
Taherian_2002	IUGR	intrauterine growth retardation (IUGR), according to Divon et al
Taherian_2002	preterm delivery	before 37 weeks of gestation
Taherian_2002	perinatal death	no definition given
Trudinger_1988	NIC admission	no definition given
Trudinger_1998	perinatal death	stillbirths + neonatal death
Tulppala_1997	preeclampsia	blood pressure ˘140/95 mm Hgsignificant). Pre-eclampsia (blood pressure ˘140/95 mm Hg and proteinuria .0.3 g/day
Tulppala_1997	IUGR	birth weight less than 10th percentile for gestational age
Tulppala_1997	gestational age	gestational age in weeks
Tulppala_1997	birth weight	actual birth weight in gramms

Vainio_2002	preeclampsia	Pre-eclampsia was defined as blood pressure changes as above and proteinuria (defined as > 300mg/24h or 1þ dipstick in a random urine sample). Superimposed preeclampsia was defined as proteinuria developing during pregnancy in a woman with known chronic hypertension
Vainio_2002	IUGR	birth weight less than 10th percentile for gestational age
Vainio_2002	gestational age	gestational age in weeks
Vainio_2002	birth weight	actual birth weight in grams
Viinikka_1993	preeclampsia	exacerbation of pre-existing hypertension, (usually >160/120 mmHg), necessitating the initiation of antihypertensive treatment, (usually dihydralazine or beta blockers), or, if they were administered before pregnancy, the increase of dose during the prophylaxis, or the rise of blood pressure (usually

		>160/ 110 mmHg) in those normotensive before pregnancy + The appearance of proteinuria greater than 300 mg/24 h.
Viinikka_1993	gestational age	gestational age in weeks
Viinikka_1993	birth weight	actual birth weight in grams
Viinikka_1993	IUGR	birth weight less than 2SD for gestational age
Viinikka_1993	NIC admission	no definition given
Viinikka_1993	perinatal death	no definition given
Villa_2012	preeclampsia	(blood pressure ‡140 and/or 90 mmHg in two consecutive measurements and proteinuria ‡0.3 g/24 hours)
Wallenburg_1986	preeclampsia	PIH and concomitant proteinuria (>0 - 5 g/1) in the absence of a urinary tract infection was defined as pre-eclampsia.
Wallenburg_1986	birth weight	actual birth weight in grams
Wallenburg_1986	IUGR	birth weight less than 3rd and 10th percentile for gestational age
Yu_2003	preeclampsia	pre-eclampsia, as defined by the International Society for the Study of

		Hypertension in Pregnancy12. This requires two recordings of diastolic blood pressure of 90 mmHg or higher at least 4 h apart or one recording of diastolic blood pressure of at least 120 mm Hg, in a previously normotensive woman, and urine protein excretion of at least 300 mg in 24 h or two readings of 2+ or higher on dipstick analysis of midstream or catheter urine specimens if no 24-h collection is available.
Yu_2003	preterm delivery	preterm delivery (before 37 weeks of gestation) and early preterm delivery (before 34 weeks)
Yu_2003	perinatal death	neonatal + intrauterine deaths
Yu_2003	NIC admission	no definition given
Yu_2003	placental abruption	clinical diagnosis of antepartum hemorrhage and abdominal pain associated with the finding of retroplacental clot at delivery
Yu_2003	IUGR	birth weight less than 5th percentile for gestational age

Zimmermann_1996	preeclampsia	Pre-eclampsia was defined as blood pressure of 145/85 mmHg or higher on two or more measurements and proteinuria on dipstick testing of a midstream urine specimen on two or more occasions more than 24 h apart, occurring for the first time in the second half of pregnancy
Zimmermann_1996	perinatal death	Intrauterine death
Zimmermann_1996	placental abruption	/uterine hemorrhage
Zimmermann_1996	IUGR	birth weight less than 10th percentile for gestational age
Zimmermann_1996	preterm delivery	delivery before week 37
Zimmermann_1996	gestational age	gestational age in weeks
Zimmermann_1996	birth weight	actual birth weight in gramms

Supplementary Table S5. Risk of bias assessment of each individual study and outcome

Unique ID	D1	D2	D3	D4	D5	Overall
Abdali_2013_preeclampsia	+	+	+	+	+	+
Abdali_2013_preterm_delivery	+	+	+	+	+	+
Abdali_2013_IUGR	+	+	+	+	+	+
Abdi_2020_preeclampsia	+	+	+	+	+	+
Abdi_2020_preterm_delivery	+	+	+	+	+	+
Abdi_2020_IUGR	+	+	+	+	+	+
Abdi_2020_gestational_age	+	+	+	+	+	+
Abdi_2020_birth_weight	+	+	+	+	+	+
Andrade_2021_preeclampsia	+	+	+	+	+	+
Ayala_2012_preeclampsia	+	+	+	+	+	+
Ayala_2012_preterm_delivery	+	+	+	+	+	+
Ayala_2012_IUGR	+	+	+	+	+	+
Ayala_2012_gestational_age	+	+	+	+	+	+
Ayala_2012_birth_weight	+	+	+	+	+	+
Ayala_2012_postpartum_haemorrhage	+	+	+	+	+	+
Ayala_2012_perinatal_death	+	+	+	+	+	+
Benigni_1989_preterm_delivery	!	+	+	+	!	!
Benigni_1989_gestational_age	!	+	+	+	!	!

Benigni_1989_birth_weight	!	+	+	+	!	!
Benigni_1989_perinatal_death						!
Beroyz_1994_postpartum_haemorrhage	!	+	+	+	!	!
Beroyz_1994_preeclampsia	!	+	+	+	+	!
Beroyz_1994_preterm_delivery	!	+	+	+	+	!
Beroyz_1994_IUGR	!	+	+	+	+	!
Beroyz_1994_gestational_age	!	+	+	+	+	!
Beroyz_1994_birth_weight	!	+	+	+	+	!
Beroyz_1994_placental_abruption	!	+	+	+	+	!
Beroyz_1994_NIC_admission	!	+	+	+	+	!
Blomqvist_2018_preeclampsia	+	+	+	+	+	+
Blomqvist_2018_preterm_delivery	+	+	+	+	+	+
Blomqvist_2018_IUGR	+	+	+	+	+	+
Blomqvist_2018_perinatal death						+
Blomqvist_2018_NIC_admission	+	+	+	+	+	+
Byaruhanga_1998_preeclampsia	+	+	+	+	+	+
Byaruhanga_1998_preterm_delivery	+	+	+	+	+	+
Byaruhanga_1998_gestational_age	+	+	+	+	+	+
Byaruhanga_1998_birth_weight	+	+	+	+	+	+
Byaruhanga_1998_IUGR	+	+	+	+	+	+
Byaruhanga_1998_placental abruption	+	+	+	+	+	+

Byaruhanga_perinatal_death	+	+	+	+	+	+
Byaruhanga_1998_NIC_admission	+	+	+	+	+	+
Caritis_1998_postpartum_haemorrhage	+	+	+	+	+	+
Caritis_1998_preeclampsia	+	+	+	+	+	+
Caspi_1994_preterm_delivery	+	+	+	+	+	+
Caspi_1994_IUGR	+	+	+	+	+	+
Caspi_1994_birth_weight	+	+	+	+	+	+
Caspi_1994_gestational_age	+	+	+	+	+	+
Caspi_1994_perinatal_death	+	+	+	+	+	+
Caspi_1994_placental_abruption	+	+	+	+	+	+
Chiaffarino_preeclampsia	+	+	+	+	+	+
Chiaffarino_2004_gestational_age	+	+	+	+	+	+
Chiaffarino_2004_birth_weight	+	+	+	+	+	+
Dasari_1998_birth_weight	!	+	+	!	!	!
Dasari_1998_gestational_age	!	+	+	!	!	!
Davies_1995_preeclampsia	+	+	+	+	+	+
Davies_1995_gestational_age	+	+	+	+	+	+
Davies_1995_placental_abruption	+	+	+	+	+	+
Davies_1995_birth_weight	+	+	+	+	+	+
Davies_1995_IUGR	+	+	+	+	+	+
Davies_1995_NIC_admission	+	+	+	+	+	+

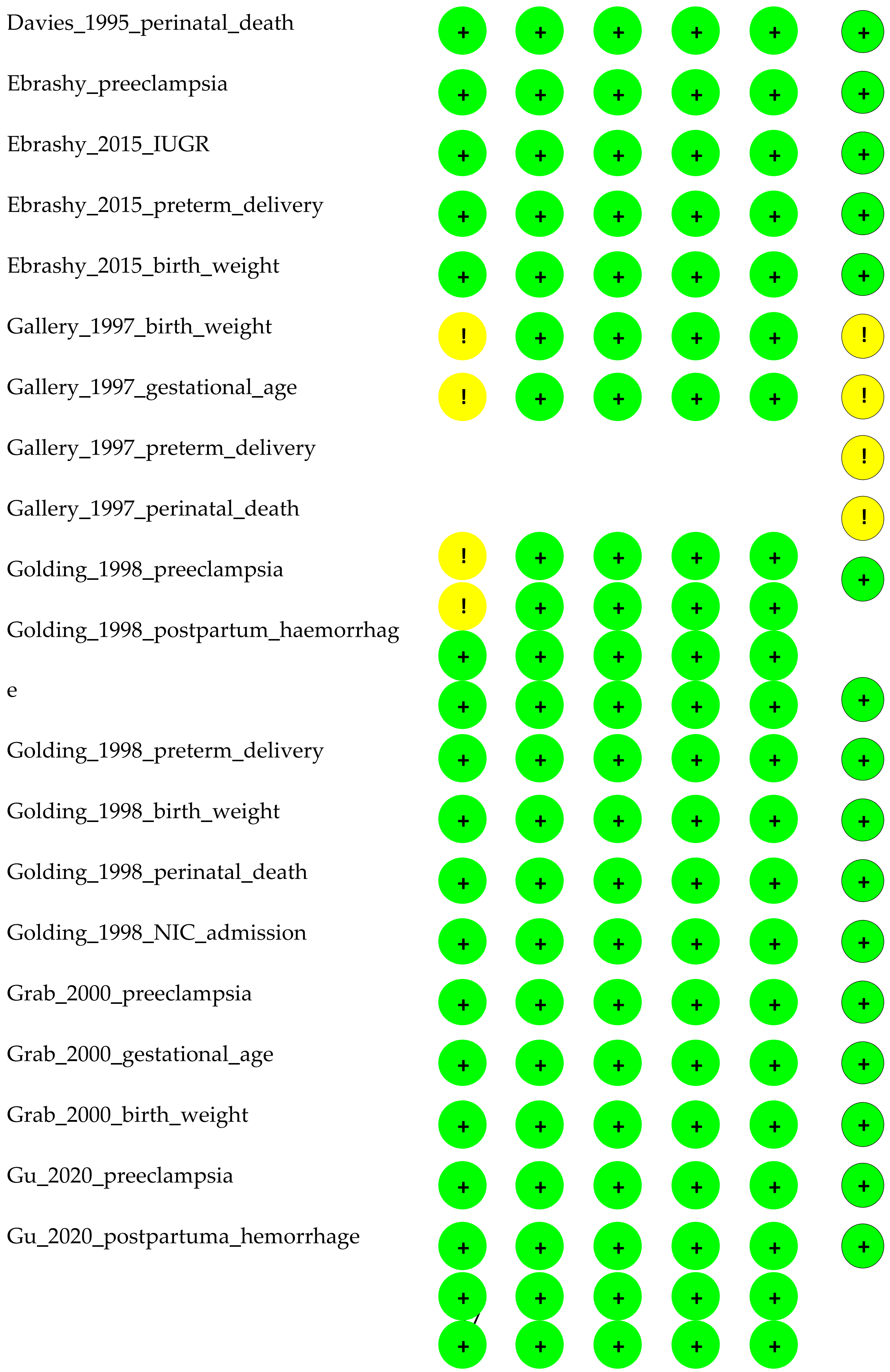

Davies_1995_perinatal_death
Ebrashy_preeclampsia
Ebrashy_2015_IUGR
Ebrashy_2015_preterm_delivery
Ebrashy_2015_birth_weight
Gallery_1997_birth_weight
Gallery_1997_gestational_age
Gallery_1997_preterm_delivery
Gallery_1997_perinatal_death
Golding_1998_preeclampsia
Golding_1998_postpartum_haemorrhag
e
Golding_1998_preterm_delivery
Golding_1998_birth_weight
Golding_1998_perinatal_death
Golding_1998_NIC_admission
Grab_2000_preeclampsia
Grab_2000_gestational_age
Grab_2000_birth_weight
Gu_2020_preeclampsia
Gu_2020_postpartuma_hemorrhage

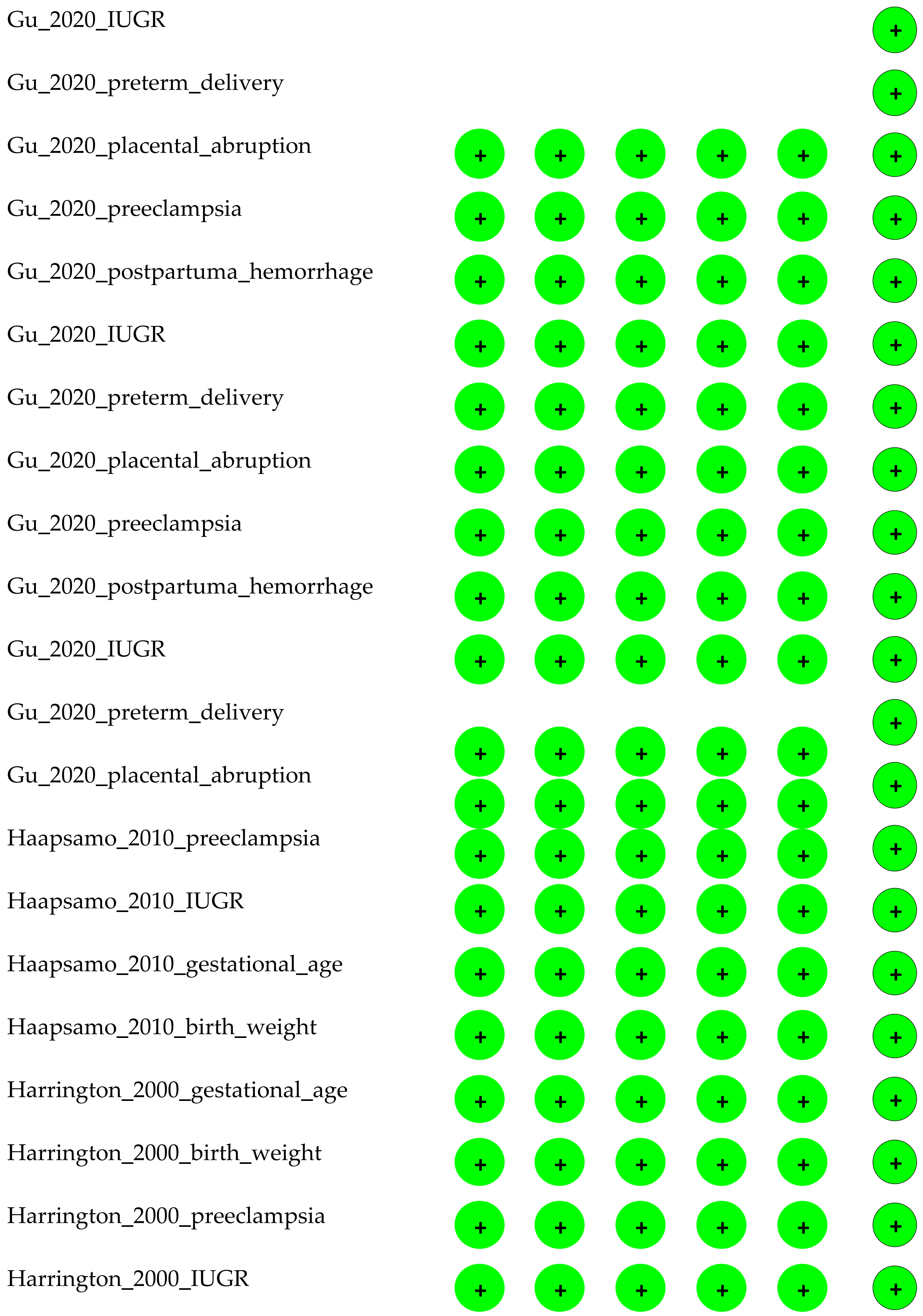
Gu_2020_IUGR
Gu_2020_preterm_delivery
Gu_2020_placental_abruption
Gu_2020_preeclampsia
Gu_2020_postpartuma_hemorrhage
Gu_2020_IUGR
Gu_2020_preterm_delivery
Gu_2020_placental_abruption
Gu_2020_preeclampsia
Gu_2020_postpartuma_hemorrhage
Gu_2020_IUGR
Gu_2020_preterm_delivery
Gu_2020_placental_abruption
Haapsamo_2010_preeclampsia
Haapsamo_2010_IUGR
Haapsamo_2010_gestational_age
Haapsamo_2010_birth_weight
Harrington_2000_gestational_age
Harrington_2000_birth_weight
Harrington_2000_preeclampsia
Harrington_2000_IUGR

Harrington_2000_placental_abruption	+	+	+	+	+	+
Harrington_2000_NIC_admission	+	+	+	+	+	+
Harrington_2000_perinatal_death	+	+	+	+	+	+
Hauth_1993_preeclampsia	+	+	+	+	+	+
Hauth_1993_IUGR	+	+	+	+	+	+
Hauth_1993_preterm_delivery	+	+	+	+	+	+
Hauth_1993_birth_weight	+	+	+	+	+	+
Hauth_1993_ perinatal_death	+	+	+	+	+	+
Herabutya_1996_preeclampsia	!	+	+	+	+	!
Hermida_1997_preeclampsia	+	+	+	+	+	+
Hermida_1997_IUGR	+	+	+	+	+	+
Hermida_1997_preterm_delivery	+	+	+	+	+	+
Hermida_1997_birth_weight	+	+	+	+	+	+
Hermida_1997_gestational_age	+	+	+	+	+	+
Hoffman_2020_preeclampsia	+	+	+	+	+	+
Hoffman_2020_postpartum_hemorrhage	+	+	+	+	+	+
Hoffman_2020_IUGR	+	+	+	+	+	+
Hoffman_2020_preterm_delivery	+	+	+	+	+	+
Hoffman_2020_perinatal_death	+	+	+	+	+	+
Kaandorp_2010_preeclampsia	+	+	+	+	+	+
Kaandorp_2010_IUGR	+	+	+	+	+	+

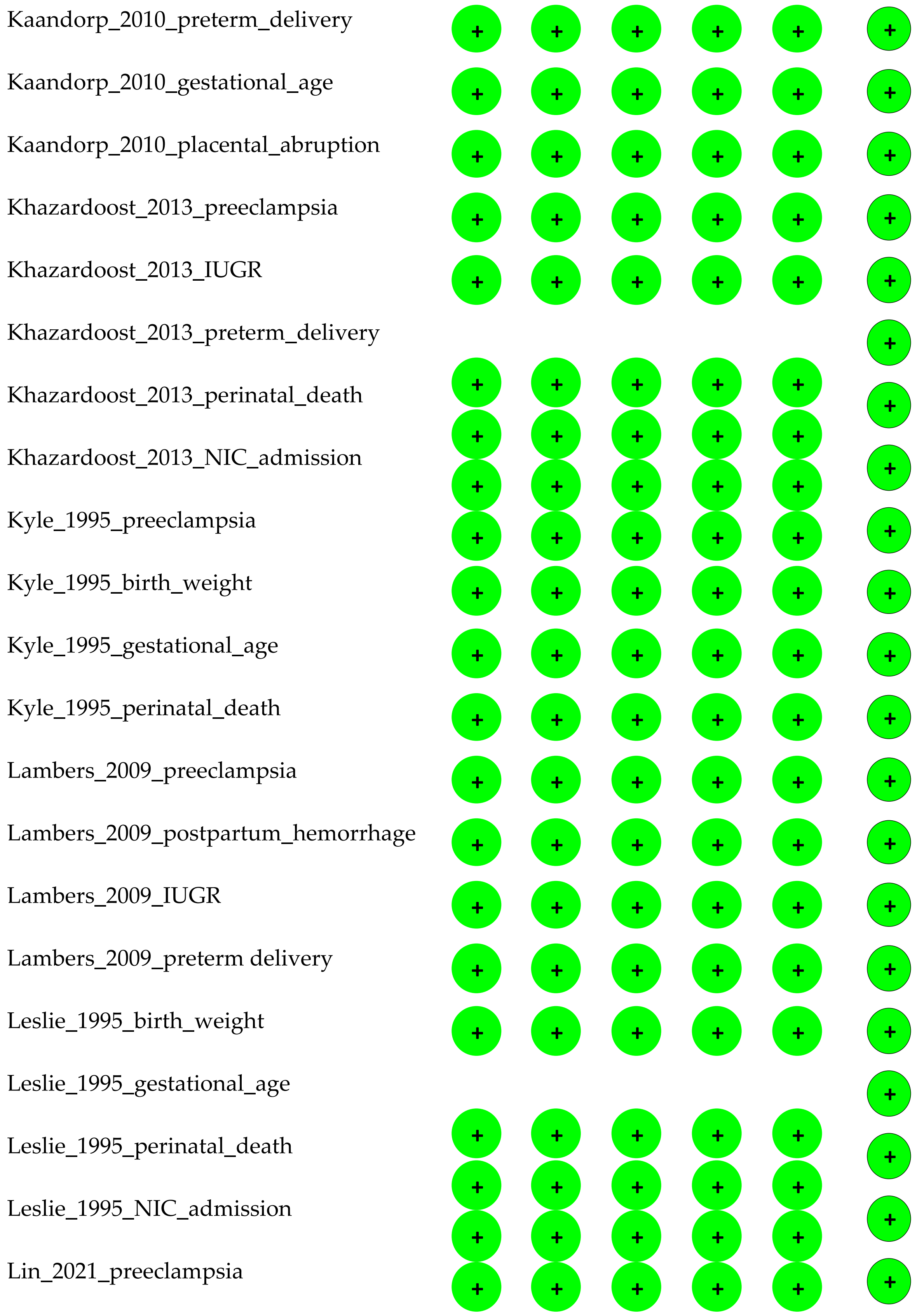
Kaandorp_2010_preterm_delivery
Kaandorp_2010_gestational_age
Kaandorp_2010_placental_abruption
Khazardoost_2013_preeclampsia
Khazardoost_2013_IUGR
Khazardoost_2013_preterm_delivery
Khazardoost_2013_perinatal_death
Khazardoost_2013_NIC_admission
Kyle_1995_preeclampsia
Kyle_1995_birth_weight
Kyle_1995_gestational_age
Kyle_1995_perinatal_death
Lambers_2009_preeclampsia
Lambers_2009_postpartum_hemorrhage
Lambers_2009_IUGR
Lambers_2009_preterm delivery
Leslie_1995_birth_weight
Leslie_1995_gestational_age
Leslie_1995_perinatal_death
Leslie_1995_NIC_admission
Lin_2021_preeclampsia
+

Lin_2021_postpartum_hemorrhage	+	+	+	+	+	+
Lin_2021_IUGR	+	+	+	+	+	+
Lin_2021_preterm_delivery	+	+	+	+	+	+
Lin_2021_birth_weight	+	+	+	+	+	+
Lin_2021_gestational_age	+	+	+	+	+	+
Lin_2021_placental_abruption	+	+	+	+	+	+
Lin_2021_perinatal_death	+	+	+	+	+	+
Lin_2021_NIC_admission	+	+	+	+	+	+

Liu_2016_preeclampsia	+	+	+	+	+	+
Liu_2016_gestational_age	+	+	+	+	+	+
Liu_2017_preeclampsia	+	+	+	+	+	+
Liu_2017_gestational_age	+	+	+	+	+	+
Liu_2017_placental_abruption	+	+	+	+	+	+
Liu_2017_perinatal_death	+	+	+	+	+	+
Liu_2017_preeclampsia	+	+	+	+	+	+
Liu_2017_gestational_age	+	+	+	+	+	+
Liu_2017_placental_abruption	+	+	+	+	+	+
Liu_2017_perinatal_death	+	+	+	+	+	+
Liu_2017_preeclampsia	+	+	+	+	+	+

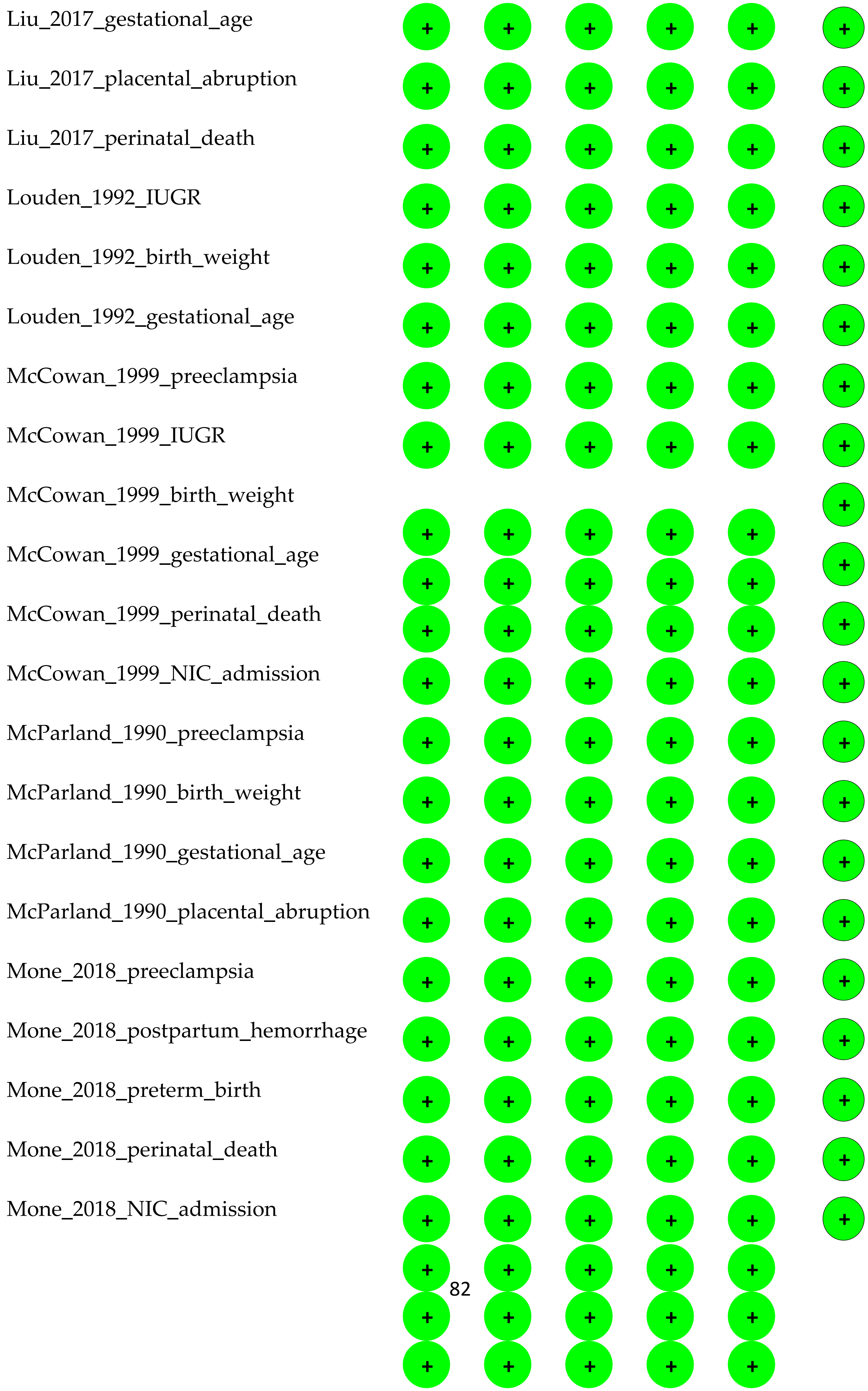
Liu_2017_gestational_age
Liu_2017_placental_abruption
Liu_2017_perinatal_death
Louden_1992_IUGR
Louden_1992_birth_weight
Louden_1992_gestational_age
McCowan_1999_preeclampsia
McCowan_1999_IUGR
McCowan_1999_birth_weight
McCowan_1999_gestational_age
McCowan_1999_perinatal_death
McCowan_1999_NIC_admission
McParland_1990_preeclampsia
McParland_1990_birth_weight
McParland_1990_gestational_age
McParland_1990_placental_abruption
Mone_2018_preeclampsia
Mone_2018_postpartum_hemorrhage
Mone_2018_preterm_birth
Mone_2018_perinatal_death
Mone_2018_NIC_admission

Morris_1996_preeclampsia						+
Morris_1996_IUGR						+
Morris_1996_preterm_birth						+
Morris_1996_birth_weight	+	+	+	+	+	+
Obido_2015_preeclampsia	+	+	+	+	+	+
Obido_2015_IUGR	+	+	+	+	+	+
Pattison_2000_IUGR	+	+	+	+	+	+
Pattison_2000_IUGR	+	+	+	+	+	+
Pattison_2000_IUGR	+	+	+	+	+	+
Pattison_2000_IUGR	+	+	+	+	+	+
Rolnik_2017_preeclampsia	+	+	+	+	+	+
Rolnik_2017_IUGR	+	+	+	+	+	+
Rolnik_2017_preterm_birth	+	+	+	+	+	+
Rolnik_2017_placental_abruption	+	+	+	+	+	+
Rolnik_2017_perinatal_death	+	+	+	+	+	+
Rolnik_2017_NIC_admission	+	+	+	+	+	+
Rotchell_1998_preeclampsia	+	+	+	+	+	+
Rotchell_1998_postpartum_hemorrhage	+	+	+	+	+	+
Rotchell_1998_birth_weight	+	+	+	+	+	+
Rotchell_1998_gestational_age	+	+	+	+	+	+

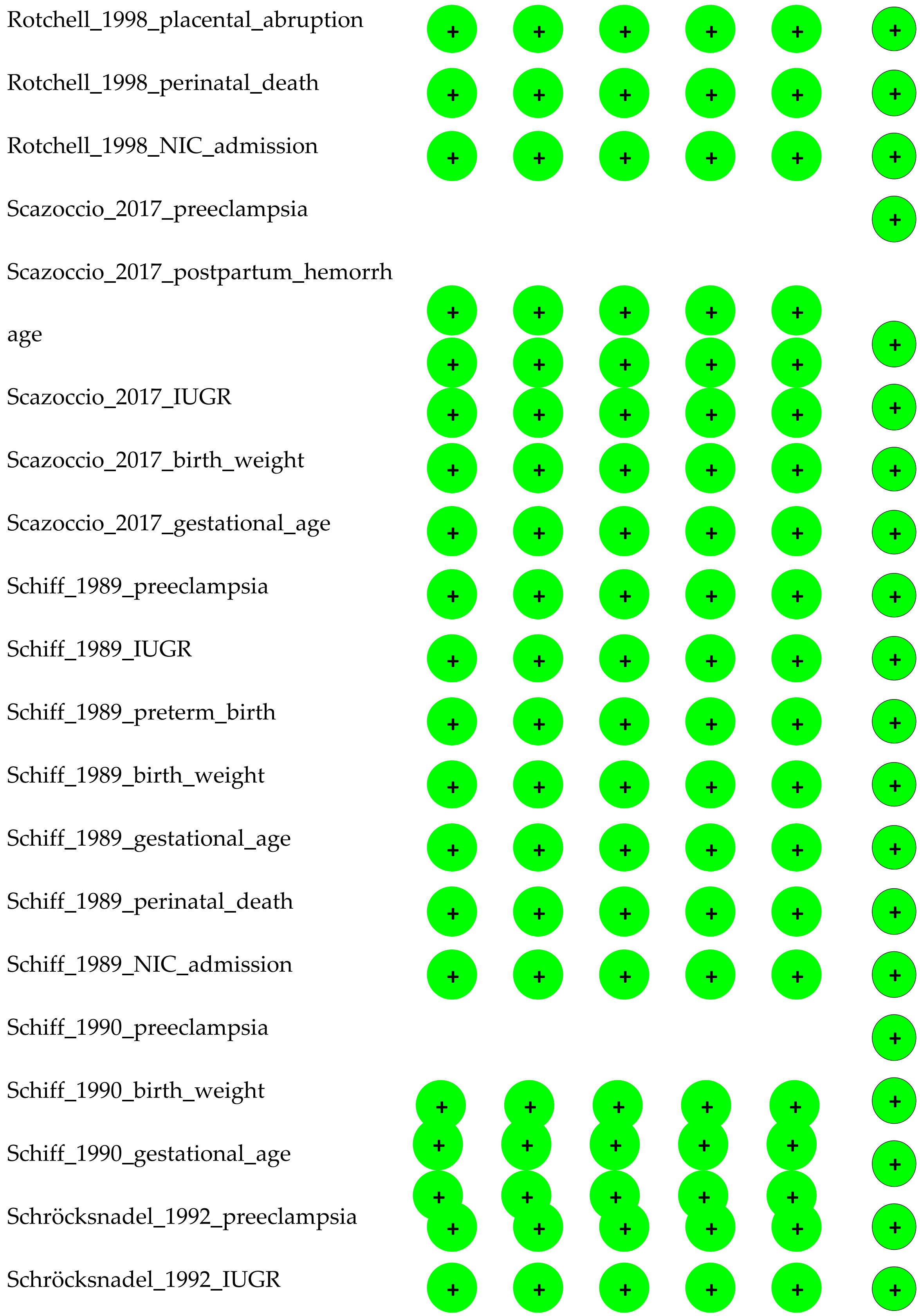
Rotchell_1998_placental_abruption
Rotchell_1998_perinatal_death
Rotchell_1998_NIC_admission
Scazoccio_2017_preeclampsia
Scazoccio_2017_postpartum_hemorrh
age
Scazoccio_2017_IUGR
Scazoccio_2017_birth_weight
Scazoccio_2017_gestational_age
Schiff_1989_preeclampsia
Schiff_1989_IUGR
Schiff_1989_preterm_birth
Schiff_1989_birth_weight
Schiff_1989_gestational_age
Schiff_1989_perinatal_death
Schiff_1989_NIC_admission
Schiff_1990_preeclampsia
Schiff_1990_birth_weight
Schiff_1990_gestational_age
Schröcksnadel_1992_preeclampsia
Schröcksnadel_1992_IUGR

Schröcksnadel_1992_preterm_birth	+	+	+	+	+	+
Schröcksnadel_1992_birth_weight	+	+	+	+	+	+
Schröcksnadel_1992_gestational_age	+	+	+	+	+	+
Schröcksnadel_1992_perinatal_death	+	+	+	+	+	+
Schröcksnadel_1992_NIC_admission	+	+	+	+	+	+
Sibai_1993_preeclampsia	+	+	+	+	+	+
Sibai_1993_IUGR	+	+	+	+	+	+
Sibai_1993_birth_weight	+	+	+	+	+	+
Sibai_1993_gestational_age	+	+	+	+	+	+
Sibai_1993_perinatal_death	+	+	+	+	+	+
Sibai_1993_NIC_admission	+	+	+	+	+	+
Stanescu_2018_preeclampsia	+	+	+	+	+	+
Stanescu_2018_IUGR	+	+	+	+	+	+
Subtil_2003_preeclampsia	+	+	+	+	+	+
Subtil_2003_postpartum_hemorrhage	+	+	+	+	+	+
Subtil_2003_IUGR	+	+	+	+	+	+
Subtil_2003_birth_weight	+	+	+	+	+	+
Subtil_2003_gestational_age	+	+	+	+	+	+
Subtil_2003_placental_abruption	+	+	+	+	+	+
Subtil_2003_perinatal_death	+	+	+	+	+	+
Subtil_2003_NIC_admission	+	+	+	+	+	+

Taherian_2002_preeclampsia	+	+	+	+	+	+
Taherian_2002_IUGR	+	+	+	+	+	+
Taherian_2002_preterm_birth	+	+	+	+	+	+
Taherian_2002_birth_weight	+	+	+	+	+	+
Taherian_2002_perinatal_death	+	+	+	+	+	+
Trudinger_1988_NIC_admission	+	+	+	+	+	+
Trudinger_1988_perinatal_death	+	+	+	+	+	+
Tulppala_1997_preeclampsia	!	+	+	+	+	!
Tulppala_1997_IUGR	!	+	+	+	+	!
Tulppala_1997_birth_weight	!	+	+	+	+	!
Tulppala_1997_gestational_age	!	+	+	+	+	!
Vainio_2002_preeclampsia	+	+	+	+	+	+
Vainio_2002_IUGR	+	+	+	+	+	+
Vainio_2002_birth_weight	+	+	+	+	+	+
Vainio_2002_gestational_age	+	+	+	+	+	+
Viinikka_1993_preeclampsia	+	+	+	+	+	+
Viinikka_1993_IUGR	+	+	+	+	+	+
Viinikka_1993_birth_weight	+	+	+	+	+	+
Viinikka_1993_gestational_age	+	+	+	+	+	+
Viinikka_1993_perinatal_death	+	+	+	+	+	+
Viinikka_1993_NIC_admission	+	+	+	+	+	+

Villa_2012_preeclampsia	+	+	+	+	+	+
Villa_2012_IUGR	+	+	+	+	+	+
Wallenburg_1986_preeclampsia	+	+	+	+	+	+
Wallenburg_1986_IUGR	+	+	+	+	+	+
Wallenburg_1986_birth_weight	+	+	+	+	+	+
Wang_1996_IUGR	+	+	+	+	+	+
Wang_1996_preterm_birth	+	+	+	+	+	+
Wang_1996_birth_weight	+	+	+	+	+	+
Wang_1996_gestational_age	+	+	+	+	+	+
Wang_1996_perinatal_death	+	+	+	+	+	+
Yu_2003_preeclampsia	+	+	+	+	+	+
Yu_2003_postpartum_hemorrhage	+	+	+	+	+	+
Yu_2003_IUGR	+	+	+	+	+	+
Yu_2003_preterm_birth	+	+	+	+	+	+
Yu_2003_placental_abruption	+	+	+	+	+	+
Yu_2003_perinatal_death	+	+	+	+	+	+
Yu_2003_NIC_admission	+	+	+	+	+	+
Zimmermann_1996_preeclampsia	+	+	+	+	+	+
Zimmermann_1996_IUGR	+	+	+	+	+	+
Zimmermann_1996_preterm_birth	+	+	+	+	+	+
Zimmermann_1996_birth_weight	+	+	+	+	+	+

Zimmermann_1996_gestational_age	+	+	+	+	+	+
Zimmermann_1996_placental_abruption	+	+	+	+	+	+
Zimmermann_1996_perinatal_death	+	+	+	+	+	+
Landman_2022_preterm_birth	+	+	+	+	+	+
Landman_2022_gestational_age	+	+	+	+	+	+
Landman_2022_postpartum_haemorrhage	+	+	+	+	+	+
Landman_2022_birth_weight	+	+	+	+	+	+
Landman_2022_IUGR	+	+	+	+	+	+
Huai_2021_preeclampsia	+	+	+	+	+	+
Huai_2021_preterm_birth	+	+	+	+	+	+
Huai_2021_placental_abruption	+	+	+	+	+	+
Huai_2021_postpartum_haemorrhage	+	+	+	+	+	+
Huai_2021_postpartum_IUGR	+	+	+	+	+	+

Supplementary Table S6. Certainty of evidence using the GRADEPro tool

Certainty assessment							№ of patients		Effect		Certainty	Importance
**№ of studies**	**Study design**	**Risk of bias**	**Inconsistency**	**Indirectness**	**Imprecision**	**Other considerations**	**ASPIRIN**	**PLACEBO / NO_TREATMENT**	**Relative (95% CI)**	**Absolute (95% CI)**		
**Preeclampsia (ASA started at any time during pregnancy)**												
46	randomised trials	not serious	not serious	not serious	not serious	dose response gradient	1352/23861 (5.7%)	1607/24233 (6.6%)	**RR 0.76** (0.64 to 0.90)	**16 fewer per 1 000** (from 24 fewer to 7 fewer)	⊕⊕⊕⊕ High	IMPORTANT
**Preeclampsia (ASA started before week 20 during pregnancy)**												
30	randomised trials	not serious	not serious	not serious	not serious	none	741/14081 (5.3%)	941/14251 (6.6%)	**RR 0.73** (0.61 to 0.87)	**18 fewer per 1 000** (from 26 fewer to 9 fewer)	⊕⊕⊕⊕ High	IMPORTANT
**Preeclampsia (ASA started after week 20 during pregnancy)**												

Certainty assessment							№ of patients		Effect		Certainty	Importance
**№ of studies**	**Study design**	**Risk of bias**	**Inconsistency**	**Indirectness**	**Imprecision**	**Other considerations**	**ASPIRIN**	**PLACEBO / NO_TREATMENT**	**Relative (95% CI)**	**Absolute (95% CI)**		
12	randomised trials	not serious	not serious	not serious	not serious	none	192/2187 (8.8%)	219/2161 (10.1%)	**RR 0.67** (0.35 to 1.28)	**33 fewer per 1 000** (from 66 fewer to 28 more)	⨁⨁⨁⨁ High	IMPORTANT
**IUGR <10% (ASA started at any time during pregnancy)**												
24	randomised trials	not serious	not serious	not serious	not serious	none	668/5739 (11.6%)	750/5764 (13.0%)	**RR 0.90** (0.81 to 1.00)	**13 fewer per 1 000** (from 25 fewer to 0 fewer)	⨁⨁⨁⨁ High	IMPORTANT
**IUGR <10% (ASA started before week 20 during pregnancy)**												
16	randomised trials	not serious	not serious	not serious	not serious	none	543/3784 (14.3%)	593/3979 (14.9%)	**RR 0.91** (0.79 to 1.04)	**13 fewer per 1 000** (from 31 fewer to 6 more)	⨁⨁⨁⨁ High	IMPORTANT

Certainty assessment							№ of patients		Effect		Certainty	Importance
№ of studies	Study design	Risk of bias	Inconsistency	Indirectness	Imprecision	Other considerations	ASPIRIN	PLACEBO / NO_TREATMENT	Relative (95% CI)	Absolute (95% CI)		
**IUGR <10% (ASA started after week 20 during pregnancy)**												
5	randomised trials	not serious	not serious	not serious	not serious	none	50/402 (12.4%)	58/402 (14.4%)	**RR 0.95** (0.65 to 1.37)	**7 fewer per 1 000** (from 50 fewer to 53 more)	⨁⨁⨁⨁ High	IMPORTANT
**IUGR <5% (ASA started at any time during pregnancy)**												
7	randomised trials	not serious	not serious	not serious	not serious	none	181/1422 (12.7%)	215/1448 (14.8%)	**RR 0.84** (0.70 to 1.00)	**24 fewer per 1 000** (from 45 fewer to 0 fewer)	⨁⨁⨁⨁ High	IMPORTANT
**IUGR <5% (ASA started before week 20 during pregnancy)**												

Certainty assessment							№ of patients		Effect		Certainty	Importance
№ of studies	Study design	Risk of bias	Inconsistency	Indirectness	Imprecision	Other considerations	ASPIRIN	PLACEBO / NO_TREATMENT	Relative (95% CI)	Absolute (95% CI)		
5	randomised trials	not serious	not serious	not serious	not serious	none	94/1066 (8.8%)	110/1095 (10.0%)	**RR 0.88** (0.68 to 1.15)	**12 fewer per 1 000** (from 32 fewer to 15 more)	⊕⊕⊕⊕ High	IMPORTANT
**IUGR <3% (ASA started at any time during pregnancy)**												
5	randomised trials	not serious	not serious	not serious	not serious	none	480/7317 (6.6%)	510/7414 (6.9%)	**RR 0.99** (0.66 to 1.47)	**1 fewer per 1 000** (from 23 fewer to 32 more)	⊕⊕⊕⊕ High	IMPORTANT
**IUGR <3% (ASA started before week 20 during pregnancy)**												
3	randomised trials	not serious	not serious	not serious	not serious	none	203/4503 (4.5%)	228/4596 (5.0%)	**RR 1.00** (0.56 to 1.77)	**0 fewer per 1 000** (from 22 fewer to 38 more)	⊕⊕⊕⊕ High	IMPORTANT

Certainty assessment							№ of patients		Effect		Certainty	Importance
№ of studies	Study design	Risk of bias	Inconsistency	Indirectness	Imprecision	Other considerations	ASPIRIN	PLACEBO / NO_TREATMENT	Relative (95% CI)	Absolute (95% CI)		
**Preterm birth (ASA started at any time during pregnancy)**												
28	randomised trials	not serious	not serious	not serious	not serious	none	2382/16596 (14.4%)	2676/17337 (15.4%)	**RR 0.84** (0.67 to 1.06)	**25 fewer per 1 000** (from 51 fewer to 9 more)	⨁⨁⨁⨁ High	IMPORTANT
**Preterm birth (ASA started before week 20 during pregnancy)**												
20	randomised trials	not serious	not serious	not serious	not serious	none	1618/12593 (12.8%)	1795/13314 (13.5%)	**RR 0.85** (0.61 to 1.19)	**20 fewer per 1 000** (from 53 fewer to 26 more)	⨁⨁⨁⨁ High	IMPORTANT
**Preterm birth (ASA started after week 20 during pregnancy)**												

Certainty assessment							№ of patients		Effect		Certainty	Importance
№ of studies	Study design	Risk of bias	Inconsistency	Indirectness	Imprecision	Other considerations	ASPIRIN	PLACEBO / NO_TREATMENT	Relative (95% CI)	Absolute (95% CI)		
8	randomised trials	not serious	not serious	not serious	not serious	none	325/2059 (15.8%)	407/2038 (20.0%)	**RR 0.79** (0.70 to 0.91)	**42 fewer per 1 000** (from 60 fewer to 18 fewer)	⊕⊕⊕⊕ High	IMPORTANT

Gestational age at delivery (ASA started at any time during pregnancy)

30	randomised trials	not serious	not serious	not serious	not serious	none	11340	11368	-	MD **0.3 week more** (0.07 more to 0.52 more)	⊕⊕⊕⊕ High	IMPORTANT

Gestational age at delivery (ASA started before week 20 during pregnancy)

Certainty assessment							№ of patients		Effect		Certainty	Importance
№ of studies	Study design	Risk of bias	Inconsistency	Indirectness	Imprecision	Other considerations	ASPIRIN	PLACEBO / NO_TREATMENT	Relative (95% CI)	Absolute (95% CI)		
15	randomised trials	not serious	not serious	not serious	not serious	none	1205	1208	-	MD **0.38 weeks more** (0.1 more to 0.67 more)	⨁⨁⨁⨁ High	IMPORTANT
**Gestational age at delivery (ASA started after week 20 during pregnancy)**												
9	randomised trials	not serious	not serious	not serious	not serious	none	386	386	-	MD **0.25 weeks more** (0.38 fewer to 0.87 more)	⨁⨁⨁⨁ High	IMPORTANT
**Actual birth weight (ASA started at any time during pregnancy)**												

Certainty assessment							№ of patients		Effect		Certainty	Importance
**№ of studies**	**Study design**	**Risk of bias**	**Inconsistency**	**Indirectness**	**Imprecision**	**Other considerations**	**ASPIRIN**	**PLACEBO / NO_TREATMENT**	**Relative (95% CI)**	**Absolute (95% CI)**		
33	randomised trials	not serious	not serious	not serious	not serious	none	11894	14897	-	MD **65.54 gramms more** (28.06 more to 103.02 more)	⨁⨁⨁⨁ High	IMPORTANT

Actual birth weight (ASA started before week 20 during pregnancy)

15	randomised trials	not serious	not serious	not serious	not serious	none	1227	1172	-	MD **99.87 gramms more** (32.35 more to 167.39 more)	⨁⨁⨁⨁ High	IMPORTANT

Actual birth weight (ASA started after week 20 during pregnancy)

Certainty assessment							№ of patients		Effect		Certainty	Importance
№ of studies	Study design	Risk of bias	Inconsistency	Indirectness	Imprecision	Other considerations	ASPIRIN	PLACEBO / NO_TREATMENT	Relative (95% CI)	Absolute (95% CI)		
9	randomised trials	not serious	not serious	not serious	not serious	none	655	662	-	MD **42.37 gramms more** (15.96 fewer to 100.7 more)	⨁⨁⨁⨁ High	IMPORTANT
**Placental abruption (ASA started at any time during pregnancy)**												
14	randomised trials	not serious	not serious	not serious	not serious	none	172/10344 (1.7%)	149/10354 (1.4%)	**RR 1.13** (0.91 to 1.40)	**2 more per 1 000** (from 1 fewer to 6 more)	⨁⨁⨁⨁ High	IMPORTANT
**NIC admission (ASA started at any time during pregnancy)**												

Certainty assessment							№ of patients		Effect		Certainty	Importance
№ of studies	Study design	Risk of bias	Inconsistency	Indirectness	Imprecision	Other considerations	ASPIRIN	PLACEBO / NO_TREATMENT	Relative (95% CI)	Absolute (95% CI)		
23	randomised trials	not serious	not serious	not serious	not serious	none	2051/15396 (13.3%)	2148/15613 (13.8%)	**RR 0.96** (0.86 to 1.06)	**6 fewer per 1 000** (from 19 fewer to 8 more)	⊕⊕⊕⊕ High	IMPORTANT
**Perinatal death (ASA started at any time during pregnancy)**												
36	randomised trials	not serious	not serious	not serious	not serious	none	566/21391 (2.6%)	664/21387 (3.1%)	**RR 0.86** (0.77 to 0.96)	**4 fewer per 1 000** (from 7 fewer to 1 fewer)	⊕⊕⊕⊕ High	IMPORTANT
**Perinatal death (ASA started before week 20 during pregnancy)**												
20	randomised trials	not serious	not serious	not serious	not serious	none	403/13202 (3.1%)	492/13309 (3.7%)	**RR 0.82** (0.72 to 0.93)	**7 fewer per 1 000** (from 10 fewer to 3 fewer)	⊕⊕⊕⊕ High	IMPORTANT

Certainty assessment							№ of patients		Effect		Certainty	Importance
№ of studies	Study design	Risk of bias	Inconsistency	Indirectness	Imprecision	Other considerations	ASPIRIN	PLACEBO / NO_TREATMENT	Relative (95% CI)	Absolute (95% CI)		
**Perinatal death (ASA started after week 20 during pregnancy)**												
12	randomised trials	not serious	not serious	not serious	not serious	none	46/2277 (2.0%)	74/2269 (3.3%)	**RR 0.68** (0.48 to 0.98)	**10 fewer per 1 000** (from 17 fewer to 1 fewer)	⊕⊕⊕⊕ High	IMPORTANT
**Postpartum haemorrhage (ASA started at any time during the pregnancy)**												
15	randomised trials	not serious	not serious	not serious	not serious	none	1926/19016 (10.1%)	1835/19295 (9.5%)	**RR 1.13** (0.95 to 1.34)	**12 more per 1 000** (from 5 fewer to 32 more)	⊕⊕⊕⊕ High	IMPORTANT

Supplementary Figure S1. PRISMA Flowchart of article selection

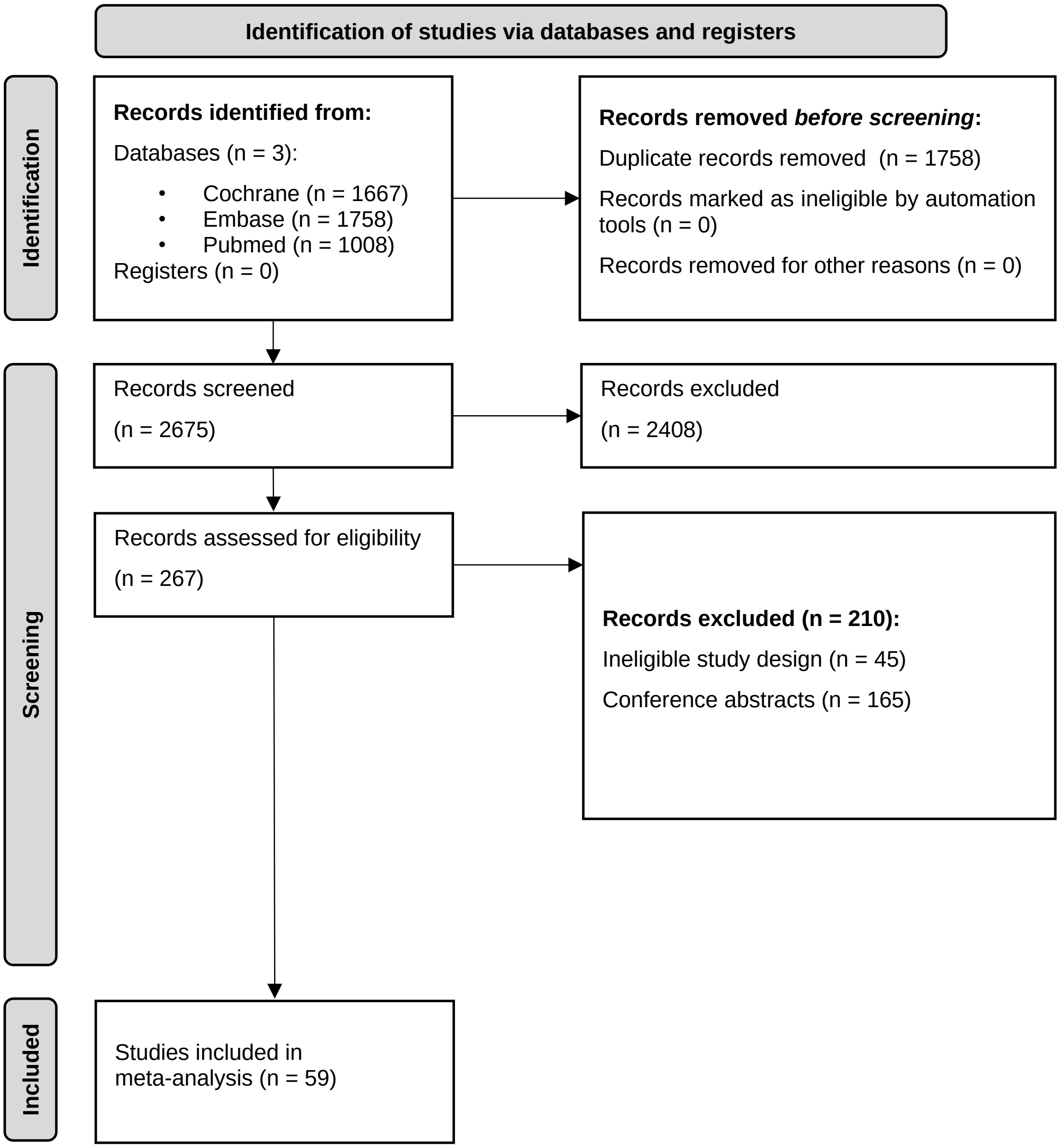

Supplementary Figure S2. Forest plot of the outcome **preeclampsia** regardless of aspirin

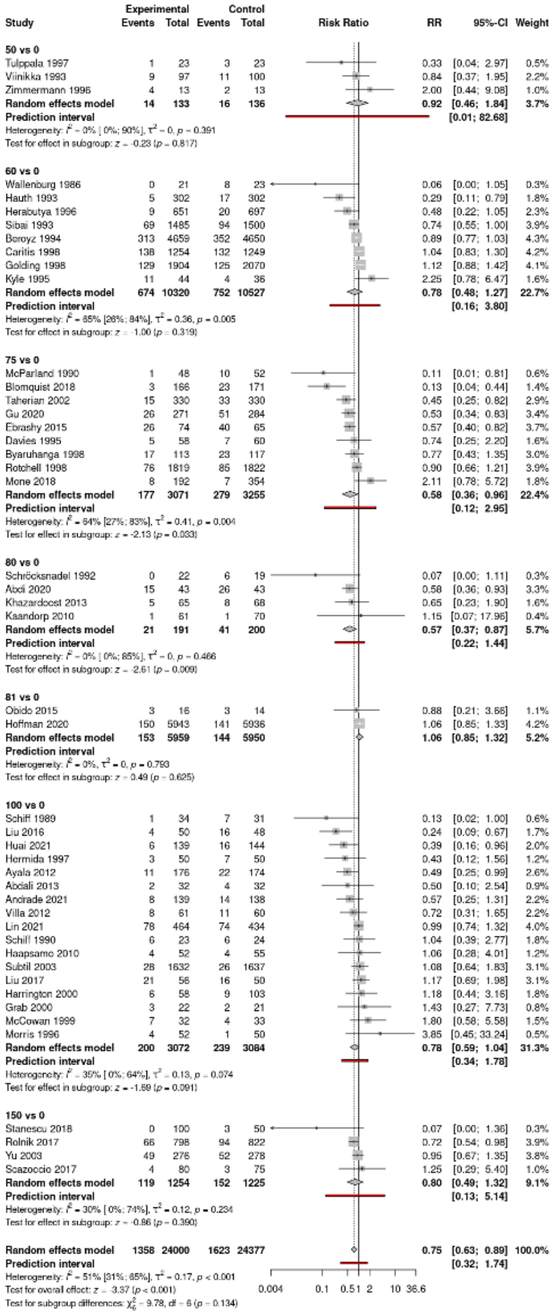

initiation time

Supplementary Figure S3. Forest plot of the outcome **intrauterine growth restriction below the 10 percentile** regardless of aspirin initiation time

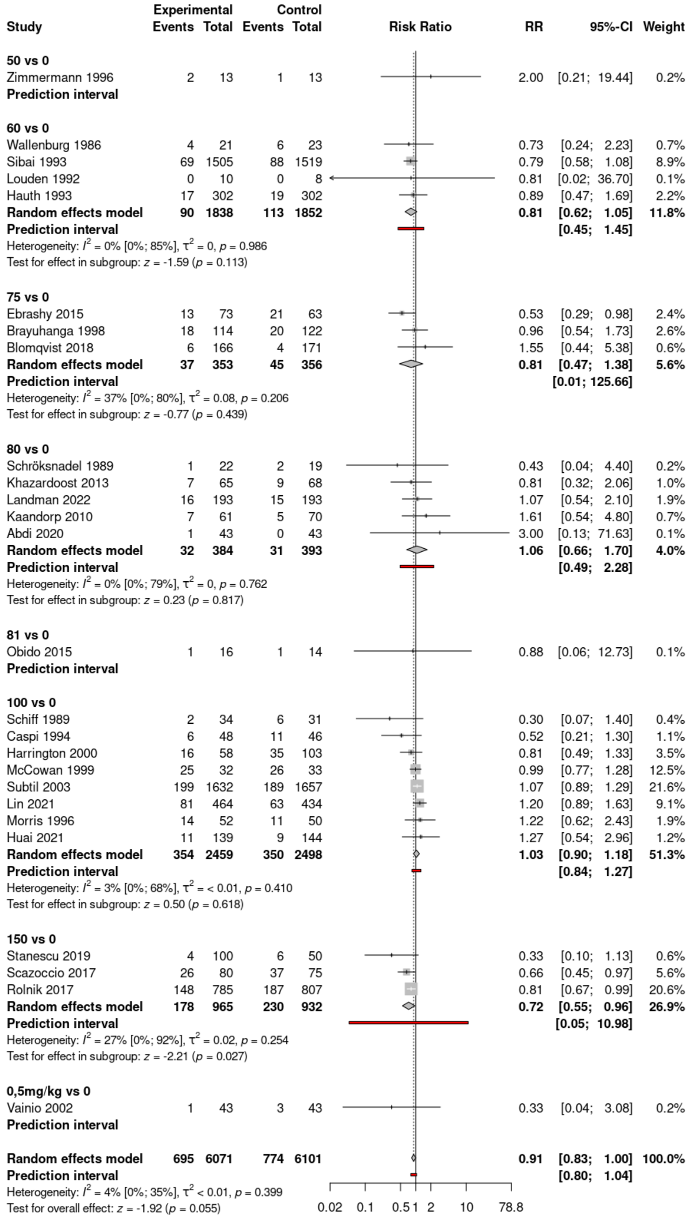

Supplementary Figure S4. Forest plot of the outcome **intrauterine growth restriction below the 5 percentile** regardless of aspirin initiation time

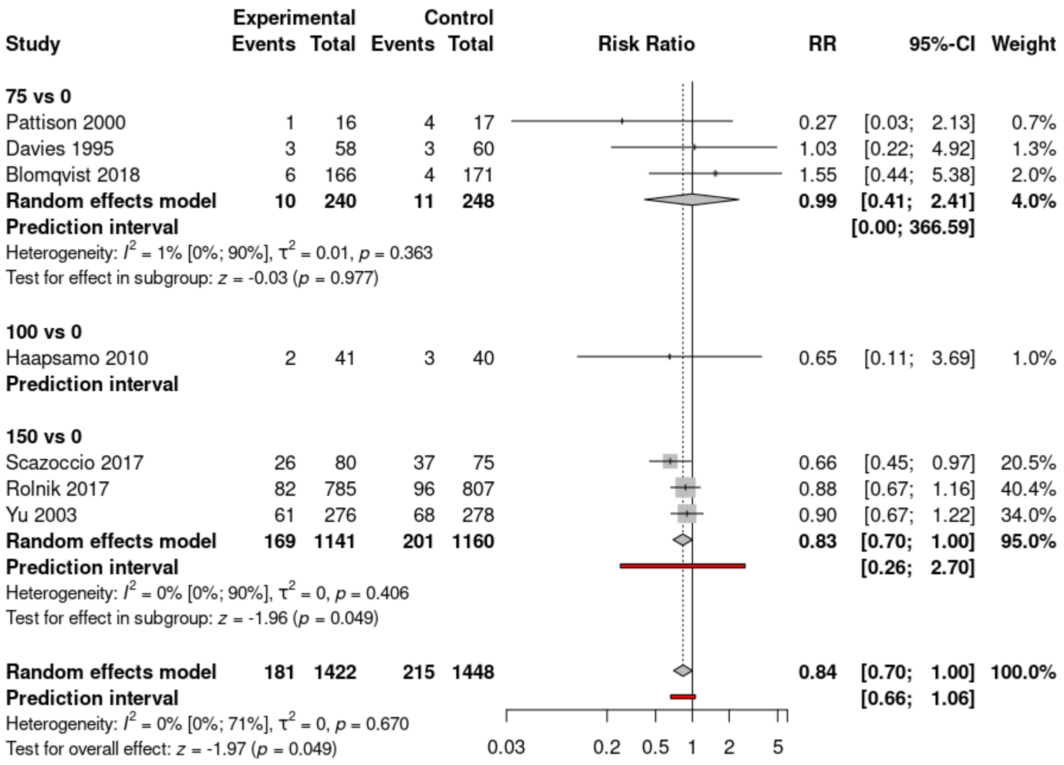

Supplementary Figure S5. Forest plot of the outcome **intrauterine growth restriction below the 3 percentile** regardless of aspirin initiation time

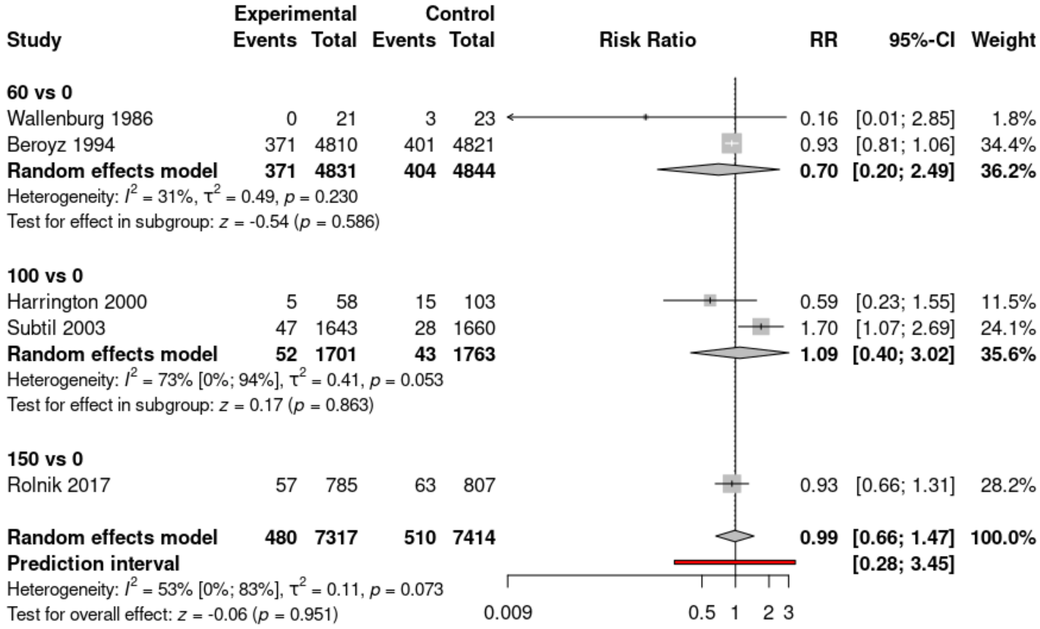

Supplementary Figure S6. Forest plot of the outcome **preterm birth before week 37** regardless of aspirin initiation time

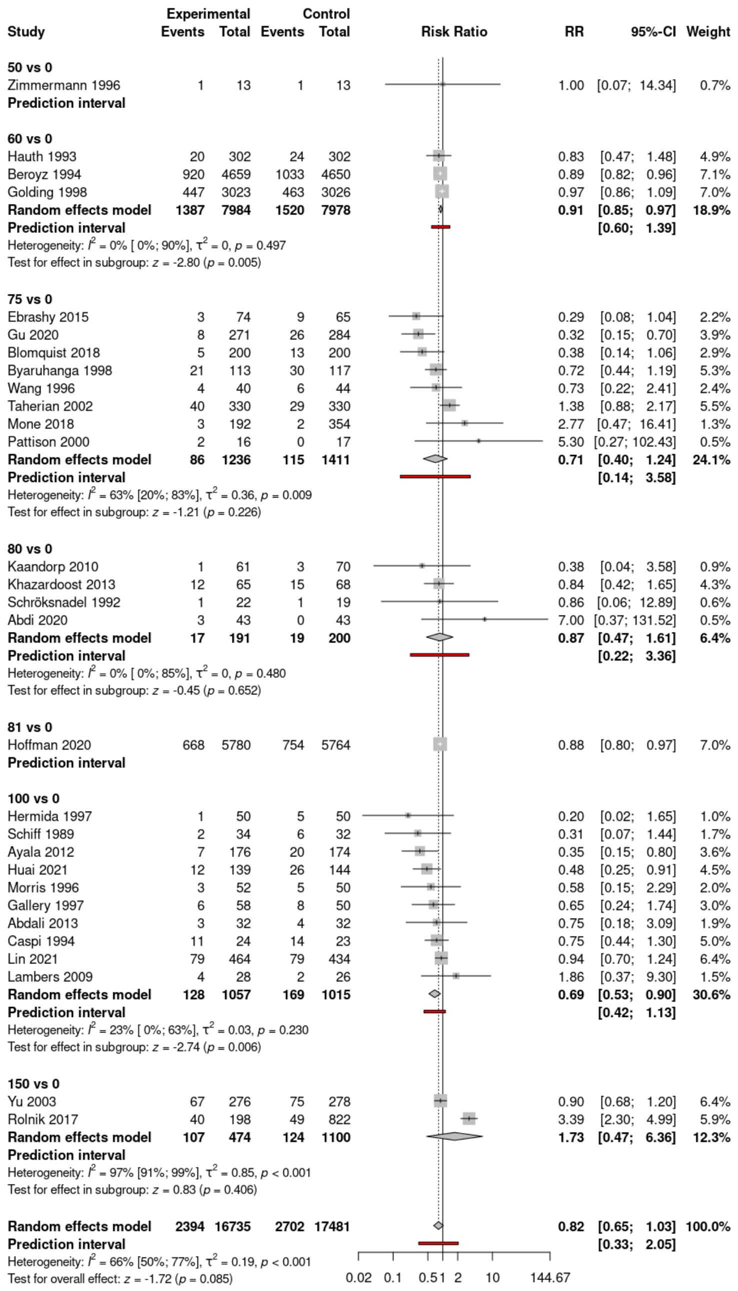

Supplementary Figure S7. Forest plot of the outcome of **gestational age at delivery (in weeks)** regardless of aspirin initiation time

Study	Experimental Total	Experimental Mean	Experimental SD	Control Total	Control Mean	Control SD	MD	95%-CI	Weight
**50 vs 0**									
Viinikka 1993	97	38.60	2.10	100	38.20	2.00	0.40	[-0.17; 0.97]	4.2%
Zimmermann 1996	13	39.20	1.60	13	38.50	3.30	0.70	[-1.29; 2.69]	0.9%
**Random effects model**	**110**			**113**			**0.42**	**[-0.13; 0.97]**	**5.1%**
**Prediction interval**									
Heterogeneity: $I^2 = 0\%$, $\tau^2 = 0$, $p = 0.777$									
Test for effect in subgroup: $z = 1.51$ ($p = 0.132$)									
**60 vs 0**									
Sibai 1993	1505	38.60	3.30	1519	38.70	2.90	-0.10	[-0.32; 0.12]	5.7%
Kyle 1995	44	39.76	1.84	36	39.77	1.91	-0.01	[-0.84; 0.82]	3.1%
Louden 1992	10	40.00	1.70	8	40.00	1.50	0.00	[-1.48; 1.48]	1.5%
Beroyz 1994	4659	38.15	3.82	4650	37.99	3.93	0.16	[0.00; 0.32]	5.9%
Benigni 1989	17	39.00	2.00	16	35.00	4.00	4.00	[1.82; 6.18]	0.8%
**Random effects model**	**6235**			**6229**			**0.49**	**[-0.55; 1.54]**	**16.9%**
**Prediction interval**								**[-3.30; 4.29]**	
Heterogeneity: $I^2 = 75\%$ [38%; 90%], $\tau^2 = 1.14$, $p = 0.003$									
Test for effect in subgroup: $z = 0.92$ ($p = 0.356$)									
**75 vs 0**									
Wang 1996	40	38.60	1.50	44	39.70	1.20	-1.10	[-1.68; -0.52]	4.1%
Rotchel 1998	1819	38.70	3.40	1822	38.50	3.70	0.20	[-0.03; 0.43]	5.6%
Byaruhanga 1998	113	37.60	3.30	117	37.10	3.70	0.50	[-0.41; 1.41]	2.8%
Liu 2017	58	36.20	3.60	50	35.60	3.30	0.60	[-0.70; 1.90]	1.8%
McParland 1990	48	39.50	2.10	52	38.70	3.90	0.80	[-0.42; 2.02]	2.0%
**Random effects model**	**2078**			**2085**			**0.10**	**[-0.61; 0.81]**	**16.3%**
**Prediction interval**								**[-2.36; 2.57]**	
Heterogeneity: $I^2 = 79\%$ [51%; 91%], $\tau^2 = 0.47$, $p < 0.001$									
Test for effect in subgroup: $z = 0.28$ ($p = 0.779$)									
**80 vs 0**									
Kaandorp 2010	61	39.50	1.30	70	39.60	1.60	-0.10	[-0.60; 0.40]	4.5%
Schröcksnadel 1992	22	39.74	1.16	19	39.79	2.43	-0.05	[-1.25; 1.15]	2.0%
Landman 2022	194	38.14	1.49	193	38.09	1.81	0.05	[-0.28; 0.38]	5.3%
Abdi 2020	43	36.79	2.63	43	36.30	3.30	0.49	[-0.77; 1.75]	1.9%
**Random effects model**	**320**			**325**			**0.02**	**[-0.24; 0.28]**	**13.7%**
**Prediction interval**								**[-0.55; 0.60]**	
Heterogeneity: $I^2 = 0\%$ [0%; 85%], $\tau^2 = 0$, $p = 0.849$									
Test for effect in subgroup: $z = 0.17$ ($p = 0.868$)									
**100 vs 0**									
McCowan 1999	49	34.40	3.40	50	34.90	3.30	-0.50	[-1.82; 0.82]	1.8%
Harrington 2000	58	38.10	3.30	103	38.30	3.90	-0.20	[-1.34; 0.94]	2.2%
Subtil 2003	1645	39.30	1.90	1660	39.30	1.90	0.00	[-0.13; 0.13]	5.9%
Dasari 1998	25	38.72	0.73	25	38.60	1.26	0.12	[-0.45; 0.69]	4.2%
Lin 2021	464	37.46	3.20	434	37.28	3.40	0.18	[-0.25; 0.61]	4.8%
Hermida 1997	50	39.60	1.41	50	39.40	2.12	0.20	[-0.51; 0.91]	3.6%
Ayala 2012	176	39.50	1.60	174	39.20	1.90	0.30	[-0.07; 0.67]	5.1%
Schiff 1990	23	38.29	2.29	24	37.86	2.57	0.43	[-0.96; 1.82]	1.6%
Liu 2016	50	38.40	4.60	48	37.60	5.50	0.80	[-1.21; 2.81]	0.9%
Caspi 1994	24	36.00	3.10	23	35.00	2.30	1.00	[-0.56; 2.56]	1.4%
Leslie 1995	54	38.60	1.47	50	37.50	2.83	1.10	[0.22; 1.98]	2.9%
Schiff 1989	34	38.86	1.14	31	37.29	0.86	1.57	[1.08; 2.06]	4.5%
Chiaffarino 2004	16	38.30	1.30	19	36.50	3.10	1.80	[0.27; 3.33]	1.4%
**Random effects model**	**2668**			**2691**			**0.46**	**[0.09; 0.82]**	**40.3%**
**Prediction interval**								**[-0.72; 1.63]**	
Heterogeneity: $I^2 = 76\%$ [59%; 86%], $\tau^2 = 0.25$, $p < 0.001$									
Test for effect in subgroup: $z = 2.47$ ($p = 0.014$)									
**150 vs 0**									
Scazoccio 2017	80	39.30	1.70	75	39.40	1.60	-0.10	[-0.62; 0.42]	4.4%
**Prediction interval**									
**0,5mg/kg vs 0**									
Vainio 2002	43	39.50	1.70	43	39.20	2.00	0.30	[-0.48; 1.08]	3.3%
**Prediction interval**									
**Random effects model**	**11534**			**11561**			**0.26**	**[0.05; 0.46]**	**100.0%**
**Prediction interval**								**[-0.65; 1.16]**	
Heterogeneity: $I^2 = 67\%$ [51%; 77%], $\tau^2 = 0.19$, $p < 0.001$									
Test for overall effect: $z = 2.42$ ($p = 0.016$)									

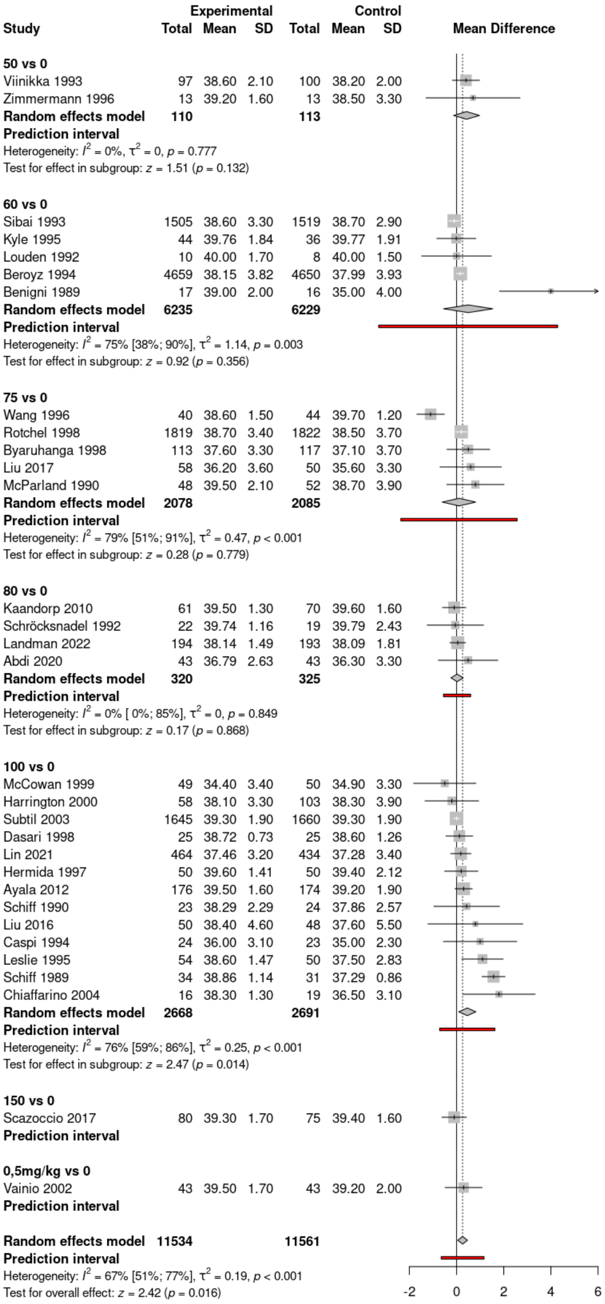

Supplementary Figure S8. Forest plot of the outcome of **actual birth weight (in grams)** regardless of aspirin initiation time

Study	Experimental Total	Experimental Mean	Experimental SD	Control Total	Control Mean	Control SD	MD	95%-CI	Weight
**50 vs 0**									
Zimmermann 1996	13	3199.00	487.00	13	3405.00	937.00	-206.00	[-780.04; 368.04]	0.2%
Tulppala 1997	33	3604.10	582.50	33	3498.20	643.97	105.90	[-190.36; 402.16]	0.6%
Viinikka 1993	97	3348.00	707.00	100	3170.00	665.00	178.00	[-13.79; 369.79]	1.3%
**Random effects model**	**143**			**146**			**130.26**	**[-24.76; 285.27]**	**2.0%**
**Prediction interval**								**[-874.70; 1135.21]**	
Heterogeneity: $I^2 = 0\%$ [0%; 90%], $\tau^2 = < 0.01$, $p = 0.453$									
Test for effect in subgroup: $z = 1.65$ ($p = 0.100$)									
**60 vs 0**									
Kyle 1995	44	3252.00	472.00	36	3335.00	449.00	-83.00	[-285.39; 119.39]	1.2%
Sibai 1993	1505	3188.00	613.00	1519	3189.00	615.00	-1.00	[-44.77; 42.77]	13.3%
Louden 1992	10	3300.00	600.00	8	3300.00	400.00	0.00	[-463.81; 463.81]	0.2%
Golding 1998	23	3041.00	519.00	3026	3023.00	533.00	18.00	[-194.95; 230.95]	1.1%
Beroyz 1994	4810	3024.00	788.00	4821	2991.00	810.00	33.00	[1.08; 64.92]	17.4%
Hauth 1993	302	3249.00	577.00	302	3169.00	553.00	80.00	[-10.14; 170.14]	5.0%
Benigni 1989	17	2922.00	599.00	16	2264.00	1072.00	658.00	[60.52; 1255.48]	0.1%
**Random effects model**	**6711**			**9728**			**24.82**	**[-3.63; 53.28]**	**38.3%**
**Prediction interval**								**[-22.85; 72.50]**	
Heterogeneity: $I^2 = 29\%$ [0%; 69%], $\tau^2 = 133.10$, $p = 0.207$									
Test for effect in subgroup: $z = 1.71$ ($p = 0.087$)									
**75 vs 0**									
Pattison 2000	16	3038.00	790.00	17	3367.00	582.00	-329.00	[-804.80; 146.80]	0.2%
Rotchel 1998	1834	3098.00	538.00	1841	3086.00	541.00	12.00	[-22.89; 46.89]	16.2%
Wang 1996	40	3056.00	259.00	44	3040.00	416.00	16.00	[-130.80; 162.80]	2.2%
Byaruhanga 1998	114	2774.00	693.00	122	2694.00	866.00	80.00	[-119.49; 279.49]	1.2%
McParland 1990	48	3068.00	555.00	52	2954.00	852.00	114.00	[-165.78; 393.78]	0.6%
Ebrashy 2015	74	3065.40	798.00	65	2948.30	812.00	117.10	[-151.27; 385.47]	0.7%
**Random effects model**	**2126**			**2141**			**15.41**	**[-17.48; 48.30]**	**21.1%**
**Prediction interval**								**[-31.19; 62.00]**	
Heterogeneity: $I^2 = 0\%$ [0%; 75%], $\tau^2 = < 0.01$, $p = 0.626$									
Test for effect in subgroup: $z = 0.92$ ($p = 0.359$)									
**80 vs 0**									
Landman 2022	194	3046.00	752.00	193	3109.00	724.00	-63.00	[-210.07; 84.07]	2.1%
Abdi 2020	43	2778.60	754.66	43	2729.06	721.66	49.54	[-262.56; 361.64]	0.5%
Schröcksnadel 1992	22	3336.00	395.00	19	3218.00	661.00	118.00	[-221.97; 457.97]	0.4%
**Random effects model**	**259**			**255**			**-21.23**	**[-145.12; 102.67]**	**3.1%**
**Prediction interval**								**[-824.41; 781.96]**	
Heterogeneity: $I^2 = 0\%$ [0%; 90%], $\tau^2 = 0$, $p = 0.562$									
Test for effect in subgroup: $z = -0.34$ ($p = 0.737$)									
**100 vs 0**									
McCowan 1999	49	1705.00	715.00	50	1787.00	678.00	-82.00	[-356.58; 192.58]	0.7%
Subtil 2003	1645	3195.00	550.00	1660	3210.00	535.00	-15.00	[-52.00; 22.00]	15.5%
Lin 2021	464	3015.07	744.67	434	3022.94	771.75	-7.87	[-107.18; 91.44]	4.3%
Harrington 2000	107	3027.00	696.00	103	3010.00	771.00	17.00	[-181.90; 215.90]	1.2%
Schiff 1990	23	2802.00	280.00	24	2772.00	294.00	30.00	[-134.10; 194.10]	1.8%
Haapsamo 2010	41	3446.00	465.00	40	3380.00	645.00	66.00	[-179.38; 311.38]	0.8%
Morris 1996	52	3145.00	633.00	50	3049.00	576.00	96.00	[-138.71; 330.71]	0.9%
Hermida 1997	50	3265.00	452.55	50	3155.00	678.82	110.00	[-116.14; 336.14]	1.0%
Ayala 2012	176	3286.00	519.00	174	3162.00	580.00	124.00	[8.65; 239.35]	3.3%
Chiaffarino 2004	16	2790.00	340.00	19	2616.00	779.00	174.00	[-213.87; 561.87]	0.3%
Dasari 1998	25	2850.00	210.00	25	2660.00	310.00	190.00	[43.22; 336.78]	2.2%
Leslie 1995	54	3291.00	514.39	50	3029.00	714.18	262.00	[21.15; 502.85]	0.8%
Caspi 1994	24	2506.00	623.00	23	2204.00	533.00	302.00	[-29.02; 633.02]	0.5%
**Random effects model**	**2726**			**2702**			**67.38**	**[8.64; 126.12]**	**33.2%**
**Prediction interval**								**[-84.23; 218.98]**	
Heterogeneity: $I^2 = 43\%$ [0%; 70%], $\tau^2 = 3846.12$, $p = 0.051$									
Test for effect in subgroup: $z = 2.25$ ($p = 0.025$)									
**150 vs 0**									
Scazoccio 2017	80	3112.00	526.00	75	3117.00	529.00	-5.00	[-171.19; 161.19]	1.7%
**Prediction interval**									
**0,5mg/kg vs 0**									
Vainio 2002	43	3462.00	604.00	43	3553.00	767.00	-91.00	[-382.80; 200.80]	0.6%
**Prediction interval**									
**Random effects model**	**12088**			**15090**			**27.56**	**[5.09; 50.04]**	**100.0%**
**Prediction interval**								**[-23.31; 78.44]**	
Heterogeneity: $I^2 = 15\%$ [0%; 45%], $\tau^2 = 492.38$, $p = 0.222$									
Test for overall effect: $z = 2.40$ ($p = 0.016$)									

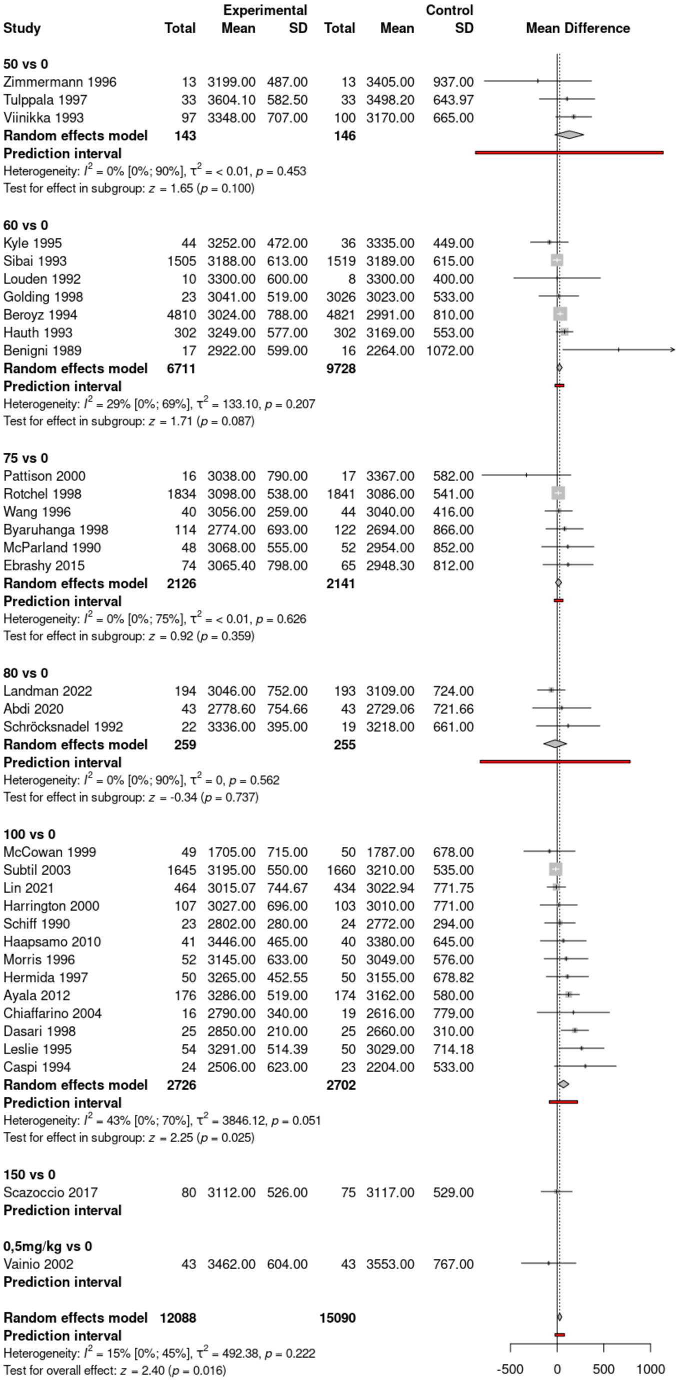

Supplementary Figure S9. Forest plot of the outcome of **placental abruption** regardless of aspirin initiation time

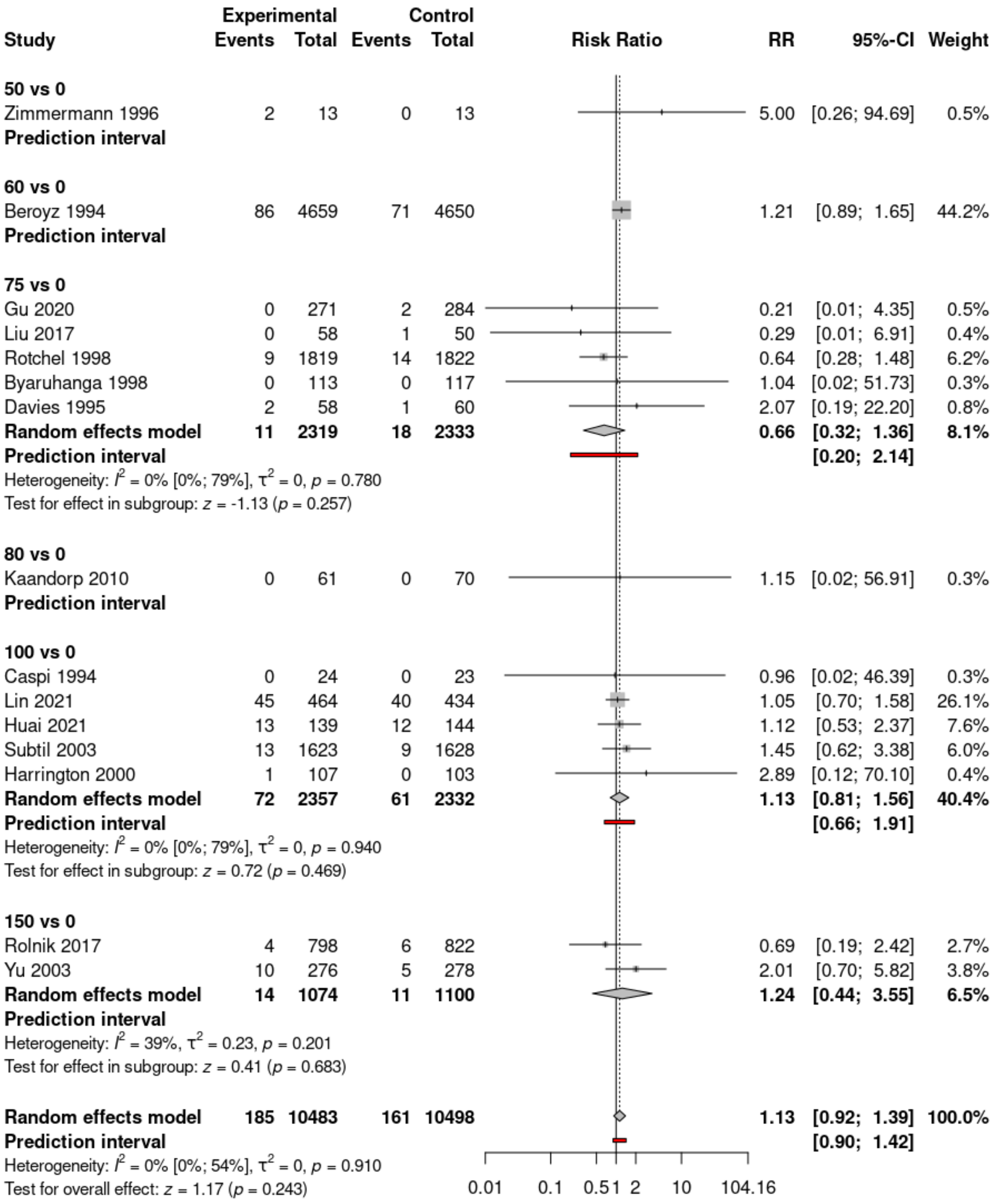

Supplementary Figure S10. Forest plot of the outcome of **neonatal intensive care unit admission** regardless of aspirin initiation time

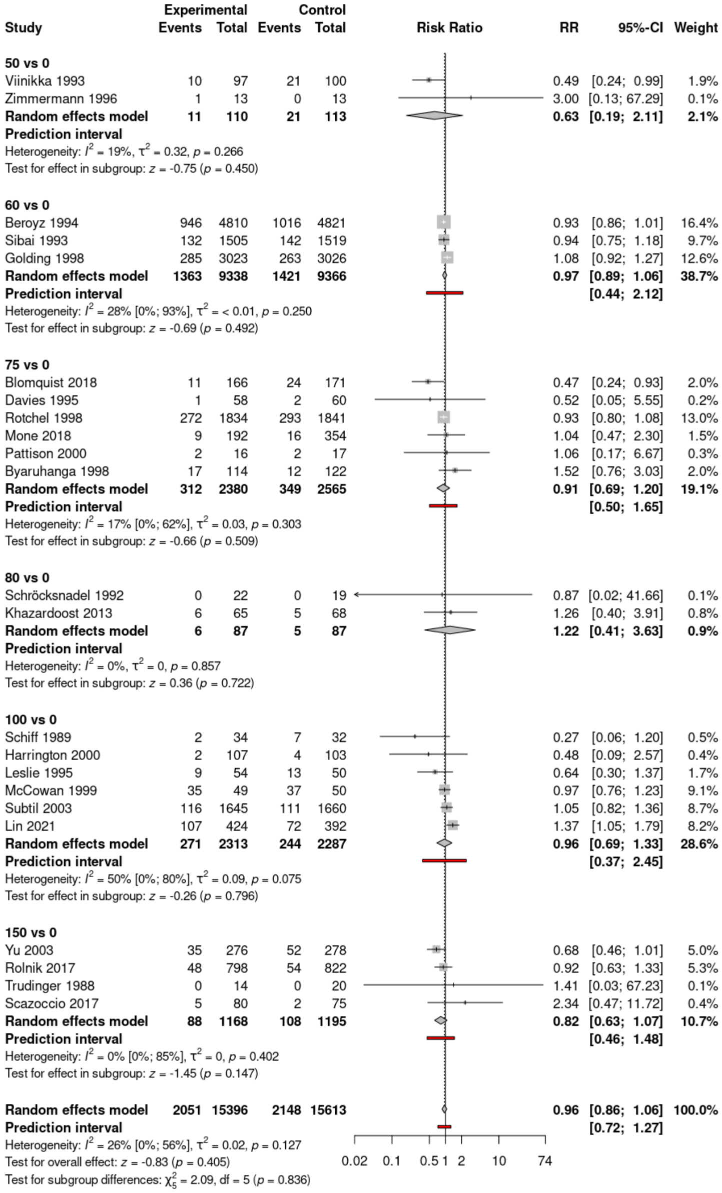

Supplementary Figure S11. Forest plot of the outcome of **perinatal death** regardless of aspirin initiation time

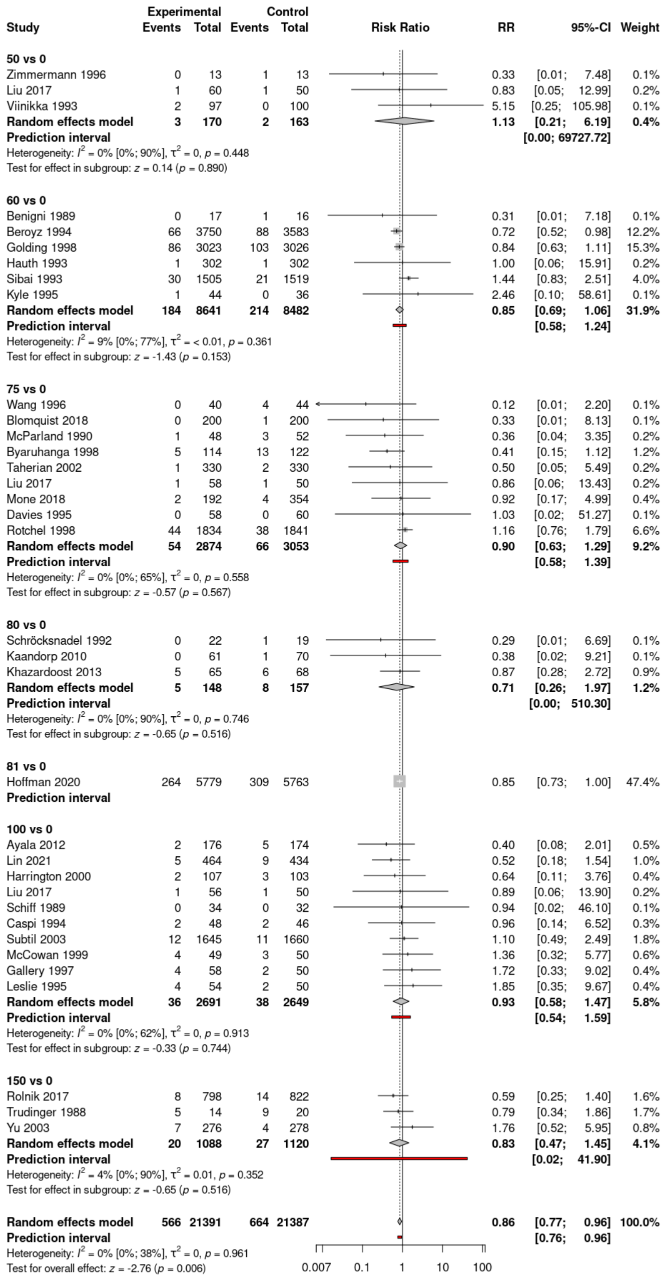

Supplementary Figure S12. Forest plot of the outcome of **postpartum hemorrhage** regardless of aspirin initiation time

Study	Experimental Events	Experimental Total	Control Events	Control Total	RR	95%-CI	Weight
**60 vs 0**							
Caritis 1998	75	1254	75	1249	1.00	[0.73; 1.36]	9.3%
Beroyz 1994	1200	4659	1182	4650	1.01	[0.95; 1.09]	12.9%
Golding 1998	145	1904	105	2070	1.50	[1.18; 1.91]	10.5%
**Random effects model**	**1420**	**7817**	**1362**	**7969**	**1.14**	**[0.88; 1.48]**	**32.7%**
**Prediction interval**						**[0.06; 23.59]**	
Heterogeneity: $I^2 = 79\%$ [31%; 93%], $\tau^2 = 0.04$, $p = 0.009$							
Test for effect in subgroup: $z = 1.00$ ($p = 0.316$)							
**75 vs 0**							
Gu 2020	6	271	15	284	0.42	[0.17; 1.06]	2.7%
Rotchell 1998	178	1819	175	1822	1.02	[0.84; 1.24]	11.2%
Mone 2018	33	192	25	354	2.43	[1.49; 3.97]	6.4%
**Random effects model**	**217**	**2282**	**215**	**2460**	**1.07**	**[0.41; 2.81]**	**20.4%**
**Prediction interval**						**[0.00; 152824.41]**	
Heterogeneity: $I^2 = 86\%$ [61%; 95%], $\tau^2 = 0.63$, $p < 0.001$							
Test for effect in subgroup: $z = 0.15$ ($p = 0.884$)							
**80 vs 0**							
Landman 2022	50	192	51	188	0.96	[0.69; 1.34]	8.8%
**Prediction interval**							
**81 vs 0**							
Hoffman 2020	54	5928	43	5907	1.25	[0.84; 1.86]	7.8%
**Prediction interval**							
**100 vs 0**							
Ayala 2012	3	176	6	174	0.49	[0.13; 1.95]	1.4%
Lin 2021	30	464	23	434	1.22	[0.72; 2.07]	5.9%
Subtil 2003	63	1634	49	1640	1.29	[0.89; 1.86]	8.3%
Huai 2021	13	139	10	144	1.35	[0.61; 2.97]	3.5%
Lambers 2009	1	28	0	26	2.79	[0.12; 65.53]	0.3%
**Random effects model**	**110**	**2441**	**88**	**2418**	**1.24**	**[0.94; 1.63]**	**19.5%**
**Prediction interval**						**[0.79; 1.93]**	
Heterogeneity: $I^2 = 0\%$ [0%; 79%], $\tau^2 = 0$, $p = 0.722$							
Test for effect in subgroup: $z = 1.51$ ($p = 0.130$)							
**150 vs 0**							
Scazoccio 2017	2	80	5	75	0.38	[0.08; 1.87]	1.1%
Yu 2003	73	276	71	278	1.04	[0.78; 1.37]	9.8%
**Random effects model**	**75**	**356**	**76**	**353**	**0.86**	**[0.40; 1.86]**	**10.9%**
**Prediction interval**							
Heterogeneity: $I^2 = 33\%$, $\tau^2 = 0.17$, $p = 0.223$							
Test for effect in subgroup: $z = -0.38$ ($p = 0.700$)							
**Random effects model**	**1926**	**19016**	**1835**	**19295**	**1.13**	**[0.95; 1.34]**	**100.0%**
**Prediction interval**						**[0.65; 1.98]**	
Heterogeneity: $I^2 = 54\%$ [18%; 74%], $\tau^2 = 0.06$, $p = 0.006$							
Test for overall effect: $z = 1.39$ ($p = 0.166$)							

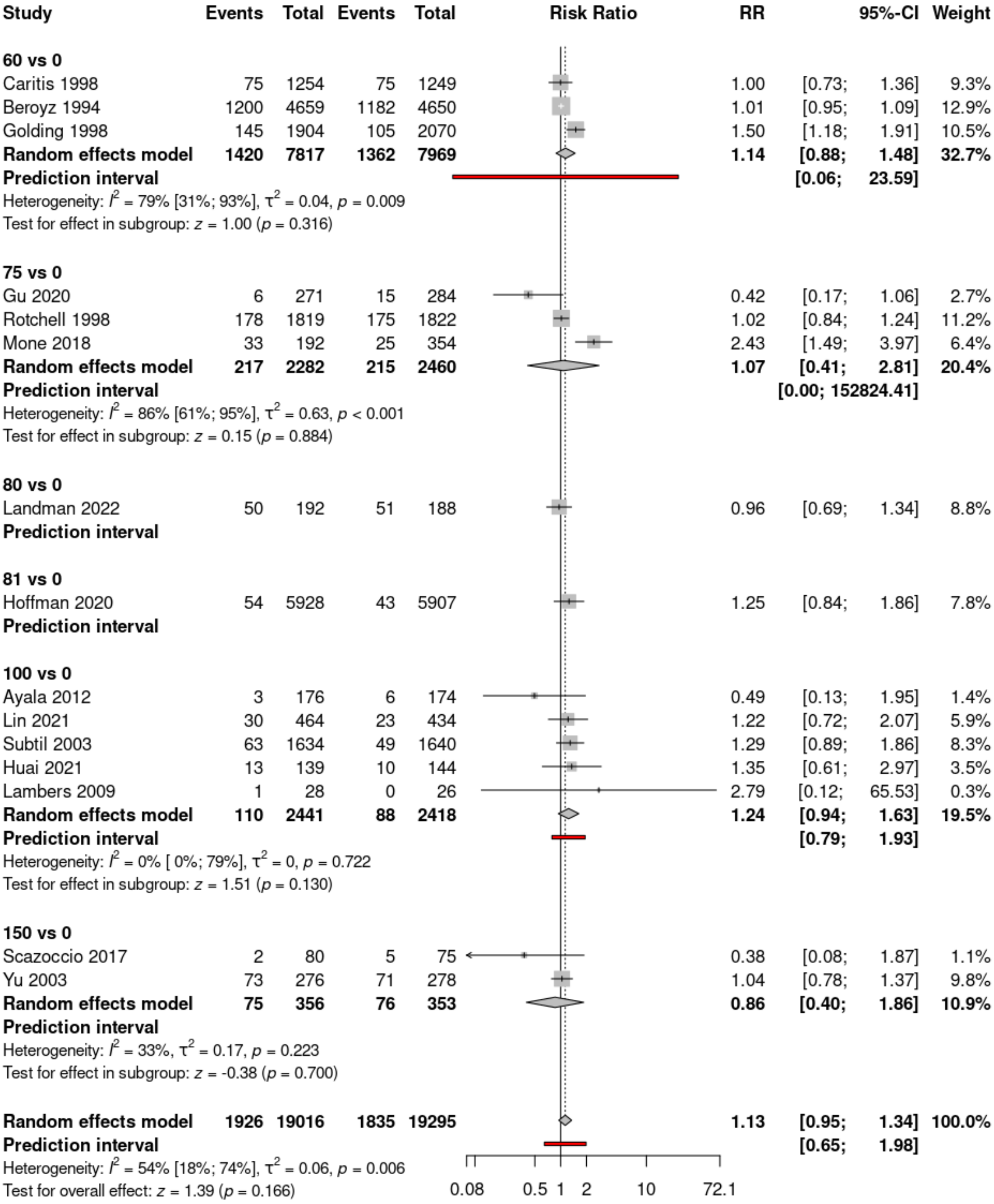

Supplementary Figure S13. Forest plot of the outcome **preeclampsia** with early initiated (<week 20) aspirin

Study	Experimental Events	Experimental Total	Control Events	Control Total	RR	95%-CI	Weight
**50 vs 0**							
Tulppala 1997	1	23	3	23	0.33	[0.04; 2.97]	1.3%
Viinikka 1993	9	97	11	100	0.84	[0.37; 1.95]	3.5%
**Random effects model**	**10**	**120**	**14**	**123**	**0.75**	**[0.34; 1.64]**	**4.8%**
**Prediction interval**							
Heterogeneity: $I^2 = 0\%$, $\tau^2 = 0$, $p = 0.437$							
Test for effect in subgroup: $z = -0.72$ ($p = 0.469$)							
**60 vs 0**							
Beroyz 1994	176	2733	222	2749	0.80	[0.66; 0.96]	5.0%
**Prediction interval**							
**75 vs 0**							
Taherian 2002	15	330	33	33	0.05	[0.03; 0.08]	4.4%
Blomquist 2018	3	166	23	171	0.13	[0.04; 0.44]	2.7%
Gu 2020	26	271	51	284	0.53	[0.34; 0.83]	4.5%
Ebrashy 2015	26	74	40	65	0.57	[0.40; 0.82]	4.7%
Davies 1995	5	58	7	60	0.74	[0.25; 2.20]	2.9%
Rotchell 1998	76	1819	85	1822	0.90	[0.66; 1.21]	4.8%
Mone 2018	8	192	7	354	2.11	[0.78; 5.72]	3.1%
**Random effects model**	**159**	**2910**	**246**	**2789**	**0.43**	**[0.17; 1.10]**	**27.2%**
**Prediction interval**						**[0.01; 12.24]**	
Heterogeneity: $I^2 = 95\%$ [92%; 97%], $\tau^2 = 1.47$, $p < 0.001$							
Test for effect in subgroup: $z = -1.77$ ($p = 0.077$)							
**80 vs 0**							
Abdi 2020	15	43	26	43	0.58	[0.36; 0.93]	4.4%
Khazardoost 2013	5	65	8	68	0.65	[0.23; 1.90]	3.0%
Kaandorp 2010	1	61	1	70	1.15	[0.07; 17.96]	0.9%
**Random effects model**	**21**	**169**	**35**	**181**	**0.60**	**[0.39; 0.92]**	**8.3%**
**Prediction interval**						**[0.04; 9.61]**	
Heterogeneity: $I^2 = 0\%$ [0%; 90%], $\tau^2 = 0$, $p = 0.876$							
Test for effect in subgroup: $z = -2.35$ ($p = 0.019$)							
**81 vs 0**							
Obido 2015	3	16	3	14	0.88	[0.21; 3.66]	2.2%
Hoffman 2020	150	5943	141	5936	1.06	[0.85; 1.33]	4.9%
**Random effects model**	**153**	**5959**	**144**	**5950**	**1.06**	**[0.85; 1.32]**	**7.2%**
**Prediction interval**							
Heterogeneity: $I^2 = 0\%$, $\tau^2 = 0$, $p = 0.793$							
Test for effect in subgroup: $z = 0.49$ ($p = 0.625$)							
**100 vs 0**							
Liu 2016	4	50	16	48	0.24	[0.09; 0.67]	3.1%
Huai 2021	6	139	16	144	0.39	[0.16; 0.96]	3.4%
Hermida 1997	3	50	7	50	0.43	[0.12; 1.56]	2.5%
Ayala 2012	11	176	22	174	0.49	[0.25; 0.99]	3.9%
Abdali 2013	2	32	4	32	0.50	[0.10; 2.54]	1.9%
Andrade 2021	8	139	14	138	0.57	[0.25; 1.31]	3.5%
Villa 2012	8	61	11	60	0.72	[0.31; 1.65]	3.5%
Lin 2021	78	464	74	434	0.99	[0.74; 1.32]	4.8%
Haapsamo 2010	4	52	4	55	1.06	[0.28; 4.01]	2.4%
Liu 2017	21	56	16	50	1.17	[0.69; 1.98]	4.3%
Harrington 2000	6	58	9	103	1.18	[0.44; 3.16]	3.2%
Grab 2000	3	22	2	21	1.43	[0.27; 7.73]	1.8%
Morris 1996	4	52	1	50	3.85	[0.45; 33.24]	1.3%
**Random effects model**	**158**	**1351**	**196**	**1359**	**0.73**	**[0.54; 0.99]**	**39.7%**
**Prediction interval**						**[0.34; 1.57]**	
Heterogeneity: $I^2 = 36\%$ [0%; 67%], $\tau^2 = 0.09$, $p = 0.095$							
Test for effect in subgroup: $z = -2.01$ ($p = 0.045$)							
**150 vs 0**							
Stanescu 2018	0	100	3	50	0.07	[0.00; 1.36]	0.8%
Rolnik 2017	66	798	94	822	0.72	[0.54; 0.98]	4.8%
Scazoccio 2017	4	80	3	75	1.25	[0.29; 5.40]	2.2%
**Random effects model**	**70**	**978**	**100**	**947**	**0.63**	**[0.18; 2.16]**	**7.8%**
**Prediction interval**						**[0.00; 365076.94]**	
Heterogeneity: $I^2 = 31\%$ [0%; 93%], $\tau^2 = 0.70$, $p = 0.234$							
Test for effect in subgroup: $z = -0.73$ ($p = 0.464$)							
**Random effects model**	**747**	**14220**	**957**	**14098**	**0.63**	**[0.47; 0.84]**	**100.0%**
**Prediction interval**						**[0.16; 2.45]**	
Heterogeneity: $I^2 = 83\%$ [77%; 88%], $\tau^2 = 0.42$, $p < 0.001$							
Test for overall effect: $z = -3.19$ ($p = 0.001$)							

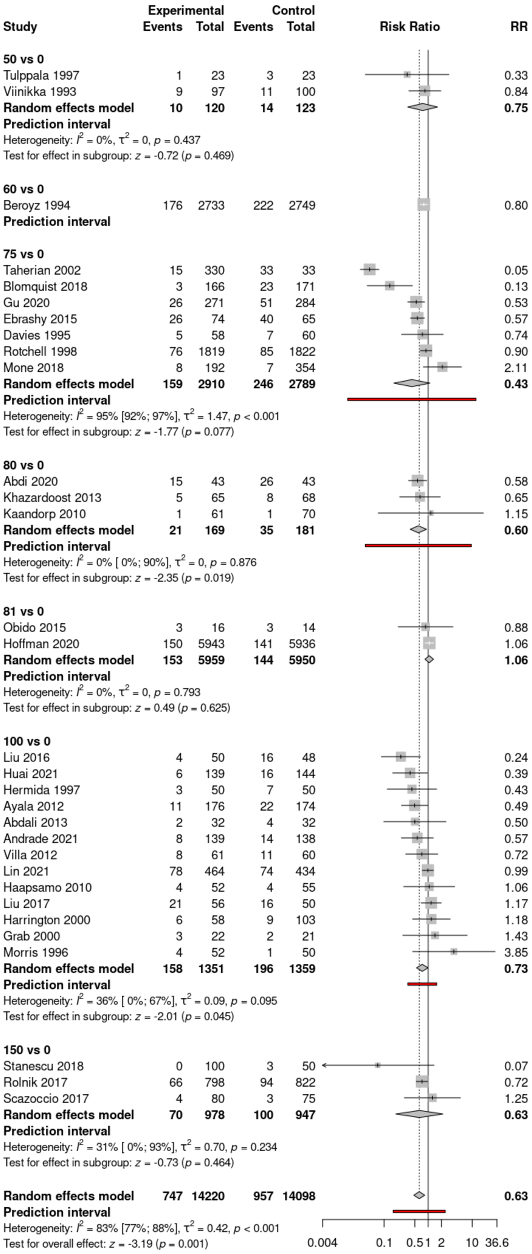

Supplementary Figure S14. Forest plot of the outcome **intrauterine growth restriction below the 10 percentile** with early initiated (<week 20) aspirin

Study	Experimental Events	Experimental Total	Control Events	Control Total	RR	95%-CI	Weight
**60 vs 0**							
Louden 1992	0	10	0	8	0.81	[0.02; 36.70]	0.1%
**75 vs 0**							
Ebrashy 2015	13	73	21	63	0.53	[0.29; 0.98]	4.6%
Brayuhanga 1998	18	114	20	122	0.96	[0.54; 1.73]	4.8%
Blomqvist 2018	6	166	4	171	1.55	[0.44; 5.38]	1.2%
**Random effects model**	**37**	**353**	**45**	**356**	**0.81**	**[0.47; 1.38]**	**10.5%**
**Prediction interval**	.	.	.	.		**[0.01; 125.66]**	--
Heterogeneity: I^2 = 37% [0%; 80%], τ^2 = 0.08, p = 0.206							
Test for effect in subgroup: z = -0.77 (p = 0.439)							
**80 vs 0**							
Schröksnadel 1989	1	22	2	19	0.43	[0.04; 4.40]	0.3%
Khazardoost 2013	7	65	9	68	0.81	[0.32; 2.06]	2.0%
Kaandorp 2010	7	61	5	70	1.61	[0.54; 4.80]	1.5%
Abdi 2020	1	43	0	43	3.00	[0.13; 71.63]	0.2%
**Random effects model**	**16**	**191**	**16**	**200**	**1.05**	**[0.54; 2.03]**	**4.0%**
**Prediction interval**	.	.	.	.		**[0.25; 4.49]**	--
Heterogeneity: I^2 = 0% [0%; 85%], τ^2 = 0, p = 0.603							
Test for effect in subgroup: z = 0.14 (p = 0.888)							
**81 vs 0**							
Obido 2015	1	16	1	14	0.88	[0.06; 12.73]	0.3%
**100 vs 0**							
Harrington 2000	16	58	35	103	0.81	[0.49; 1.33]	6.4%
Subtil 2003	199	1632	189	1657	1.07	[0.89; 1.29]	25.1%
Lin 2021	81	464	63	434	1.20	[0.89; 1.63]	14.2%
Morris 1996	14	52	11	50	1.22	[0.62; 2.43]	3.6%
**Random effects model**	**310**	**2206**	**298**	**2244**	**1.08**	**[0.93; 1.25]**	**49.3%**
**Prediction interval**	.	.	.	.		**[0.78; 1.49]**	--
Heterogeneity: I^2 = 0% [0%; 85%], τ^2 = 0, p = 0.594							
Test for effect in subgroup: z = 1.02 (p = 0.309)							
**150 vs 0**							
Stanescu 2019	4	100	6	50	0.33	[0.10; 1.13]	1.2%
Scazoccio 2017	26	80	37	75	0.66	[0.45; 0.97]	9.6%
Rolnik 2017	148	785	187	807	0.81	[0.67; 0.99]	24.5%
**Random effects model**	**178**	**965**	**230**	**932**	**0.72**	**[0.55; 0.96]**	**35.3%**
**Prediction interval**	.	.	.	.		**[0.05; 10.98]**	--
Heterogeneity: I^2 = 27% [0%; 92%], τ^2 = 0.02, p = 0.254							
Test for effect in subgroup: z = -2.21 (p = 0.027)							
**0,5mg/kg vs 0**							
Vainio 2002	1	43	3	43	0.33	[0.04; 3.08]	0.4%
**Random effects model**	**543**	**3784**	**593**	**3797**	**0.91**	**[0.79; 1.04]**	**100.0%**
**Prediction interval**						**[0.70; 1.18]**	
Heterogeneity: I^2 = 20% [0%; 55%], τ^2 < 0.01, p = 0.217							
Test for overall effect: z = -1.38 (p = 0.168)							

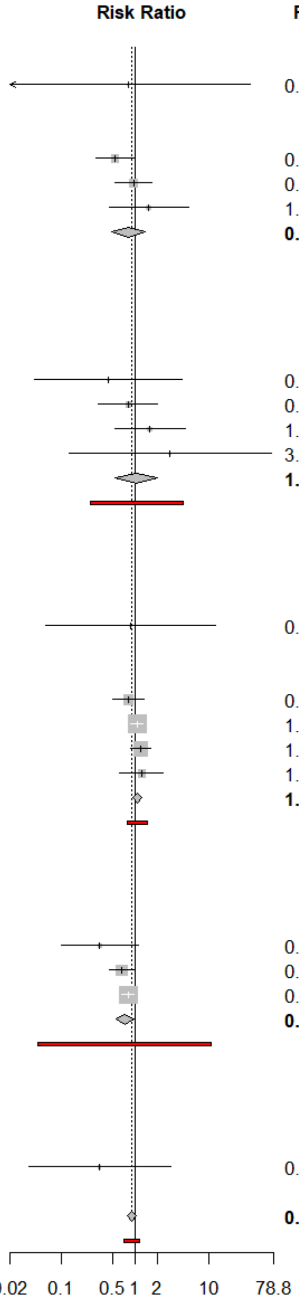

Supplementary Figure S15. Forest plot of the outcome **intrauterine growth restriction below the 5 percentile** with early initiated (<week 20) aspirin

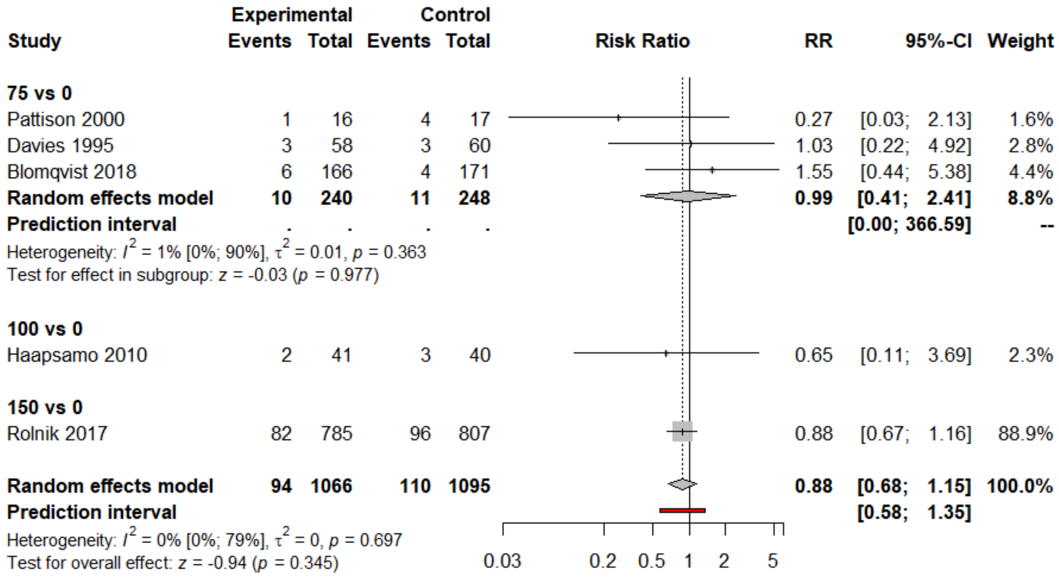

Supplementary Figure S16. Forest plot of the outcome **intrauterine growth restriction below 3 percentile** with early initiated (<week 20) aspirin

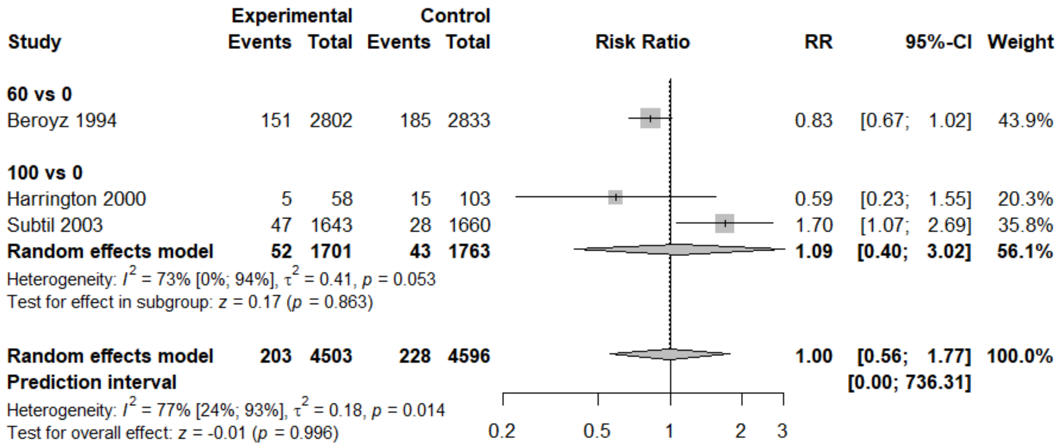
Experimental Control
Study Events Total Events Total Risk Ratio RR 95%-CI Weight
60 vs 0
Beroyz 1994 151 2802 185 2833 0.83 [0.67; 1.02] 43.9%
100 vs 0
Harrington 2000 5 58 15 103 0.59 [0.23; 1.55] 20.3%
Subtil 2003 47 1643 28 1660 1.70 [1.07; 2.69] 35.8%
Random effects model 52 1701 43 1763 1.09 [0.40; 3.02] 56.1%
Heterogeneity: I^2 = 73% [0%; 94%], τ^2 = 0.41, p = 0.053
Test for effect in subgroup: z = 0.17 (p = 0.863)
Random effects model 203 4503 228 4596 1.00 [0.56; 1.77] 100.0%
Prediction interval [0.00; 736.31]
Heterogeneity: I^2 = 77% [24%; 93%], τ^2 = 0.18, p = 0.014
Test for overall effect: z = -0.01 (p = 0.996)
0.2 0.5 1 2 3

Supplementary Figure S17. Forest plot of the outcome **preterm birth before week 37** with early initiated (<week 20) aspirin

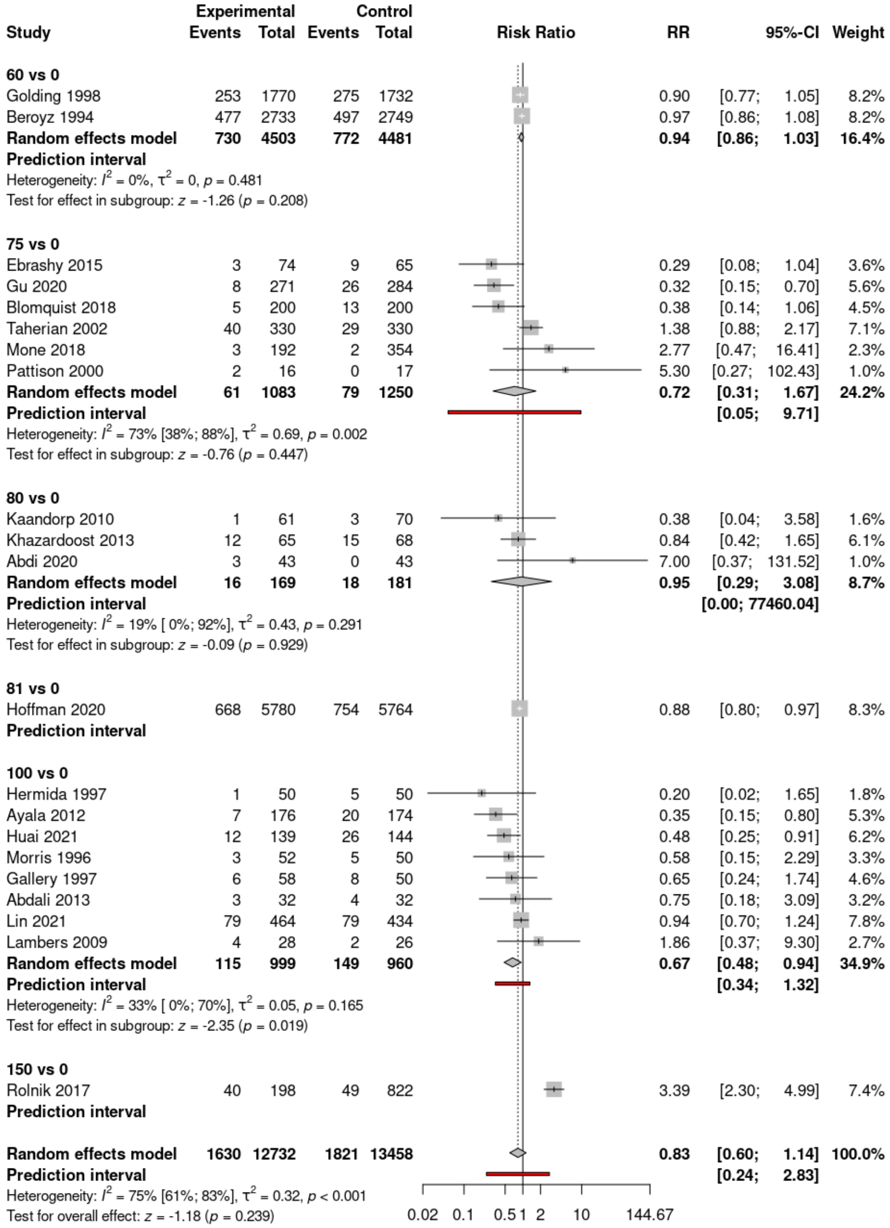

Supplementary Figure S18. Forest plot of the outcome **gestational age at delivery (in weeks)** with early initiated (<week 20) aspirin

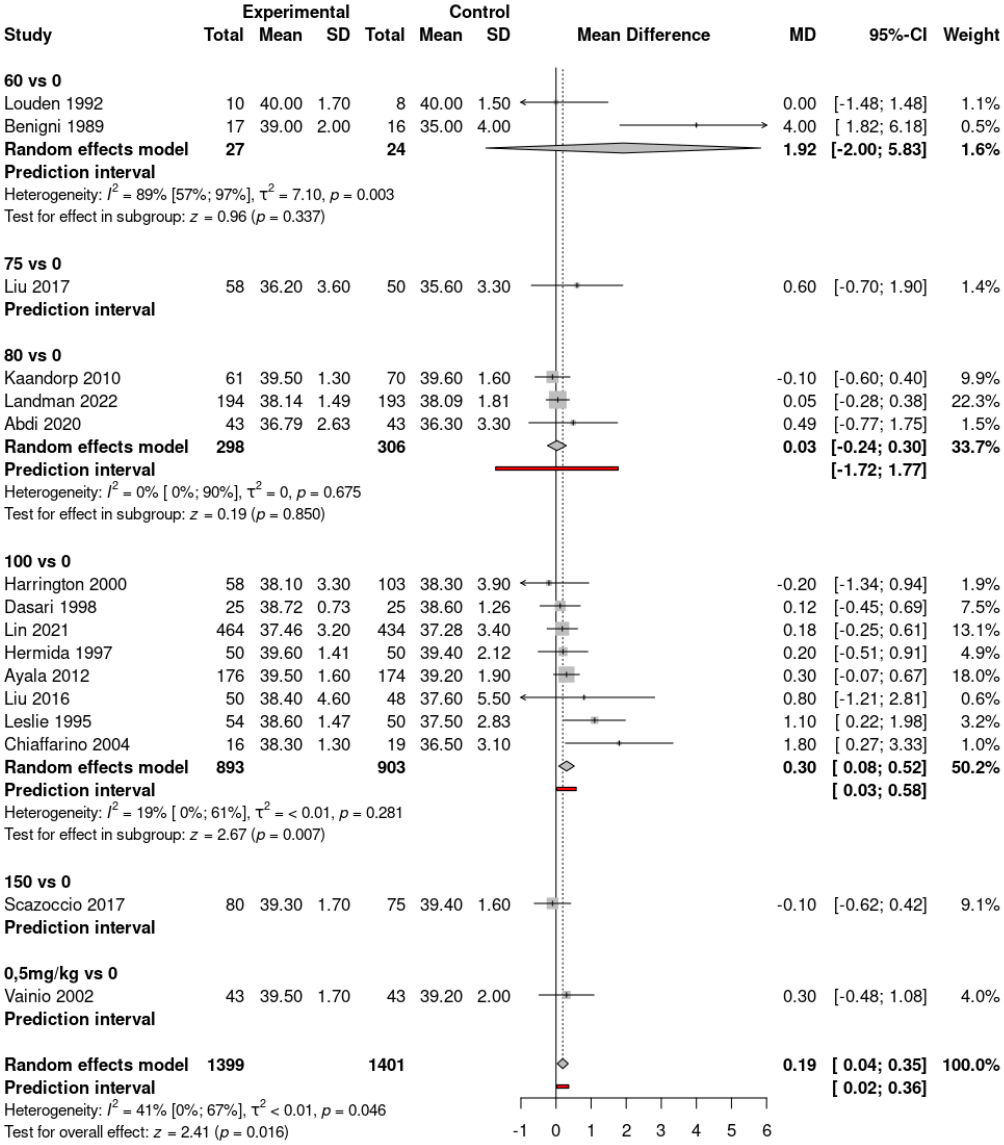

Supplementary Figure S19. Forest plot of the outcome **actual birth weight (in grams)** with early initiated (<week 20) aspirin

Study	Experimental Total	Experimental Mean	Experimental SD	Control Total	Control Mean	Control SD	MD	95%-CI	Weight
**60 vs 0**									
Louden 1992	10	3300.00	600.00	8	3300.00	400.00	0.00	[-463.81; 463.81]	1.5%
Benigni 1989	17	2922.00	599.00	16	2264.00	1072.00	658.00	[60.52; 1255.48]	0.9%
**Random effects model**	**27**			**24**			**300.94**	**[-341.54; 943.42]**	**2.5%**
**Prediction interval**									
Heterogeneity: $I^2 = 66\%$ [0%; 92%], $\tau^2 = 142017.03$, $p = 0.088$									
Test for effect in subgroup: $z = 0.92$ ($p = 0.359$)									
**75 vs 0**									
Pattison 2000	16	3038.00	790.00	17	3367.00	582.00	-329.00	[-804.80; 146.80]	1.5%
Ebrashy 2015	74	3065.40	798.00	65	2948.30	812.00	117.10	[-151.27; 385.47]	4.2%
**Random effects model**	**90**			**82**			**-60.91**	**[-489.08; 367.25]**	**5.6%**
**Prediction interval**									
Heterogeneity: $I^2 = 61\%$ [0%; 91%], $\tau^2 = 60662.40$, $p = 0.109$									
Test for effect in subgroup: $z = -0.28$ ($p = 0.780$)									
**80 vs 0**									
Landman 2022	194	3046.00	752.00	193	3109.00	724.00	-63.00	[-210.07; 84.07]	10.5%
Abdi 2020	43	2778.60	754.66	43	2729.06	721.66	49.54	[-262.56; 361.64]	3.2%
**Random effects model**	**237**			**236**			**-42.55**	**[-175.59; 90.49]**	**13.7%**
**Prediction interval**									
Heterogeneity: $I^2 = 0\%$, $\tau^2 = 0$, $p = 0.523$									
Test for effect in subgroup: $z = -0.63$ ($p = 0.531$)									
**100 vs 0**									
Lin 2021	464	3015.07	744.67	434	3022.94	771.75	-7.87	[-107.18; 91.44]	16.3%
Harrington 2000	107	3027.00	696.00	103	3010.00	771.00	17.00	[-181.90; 215.90]	6.8%
Morris 1996	52	3145.00	633.00	50	3049.00	576.00	96.00	[-138.71; 330.71]	5.2%
Hermida 1997	50	3265.00	452.55	50	3155.00	678.82	110.00	[-116.14; 336.14]	5.6%
Ayala 2012	176	3286.00	519.00	174	3162.00	580.00	124.00	[8.65; 239.35]	14.0%
Chiaffarino 2004	16	2790.00	340.00	19	2616.00	779.00	174.00	[-213.87; 561.87]	2.2%
Dasari 1998	25	2850.00	210.00	25	2660.00	310.00	190.00	[43.22; 336.78]	10.5%
Leslie 1995	54	3291.00	514.39	50	3029.00	714.18	262.00	[21.15; 502.85]	5.0%
**Random effects model**	**944**			**905**			**99.81**	**[26.32; 173.30]**	**65.6%**
**Prediction interval**								**[-65.28; 264.91]**	
Heterogeneity: $I^2 = 18\%$ [0%; 61%], $\tau^2 = 3146.38$, $p = 0.288$									
Test for effect in subgroup: $z = 2.66$ ($p = 0.008$)									
**150 vs 0**									
Scazoccio 2017	80	3112.00	526.00	75	3117.00	529.00	-5.00	[-171.19; 161.19]	8.9%
**Prediction interval**									
**0,5mg/kg vs 0**									
Vainio 2002	43	3462.00	604.00	43	3553.00	767.00	-91.00	[-382.80; 200.80]	3.6%
**Prediction interval**									
**Random effects model**	**1421**			**1365**			**62.88**	**[3.75; 122.00]**	**100.0%**
**Prediction interval**								**[-71.72; 197.48]**	
Heterogeneity: $I^2 = 27\%$ [0%; 60%], $\tau^2 = 3028.52$, $p = 0.156$									
Test for overall effect: $z = 2.08$ ($p = 0.037$)									

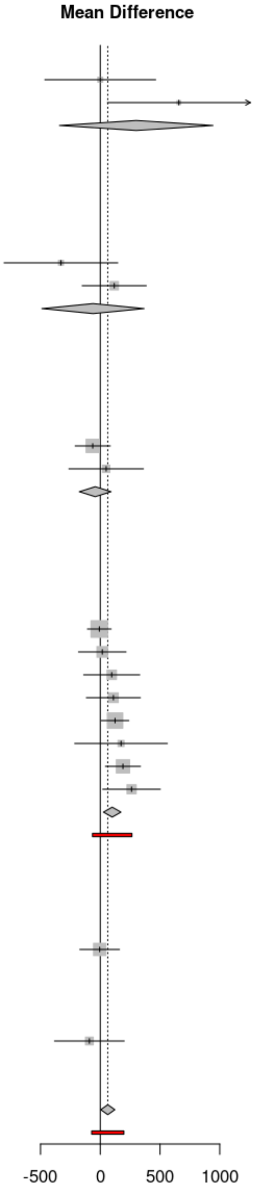

Supplementary Figure S20. Forest plot of the outcome **preeclampsia** with late initiated (>week 20) aspirin

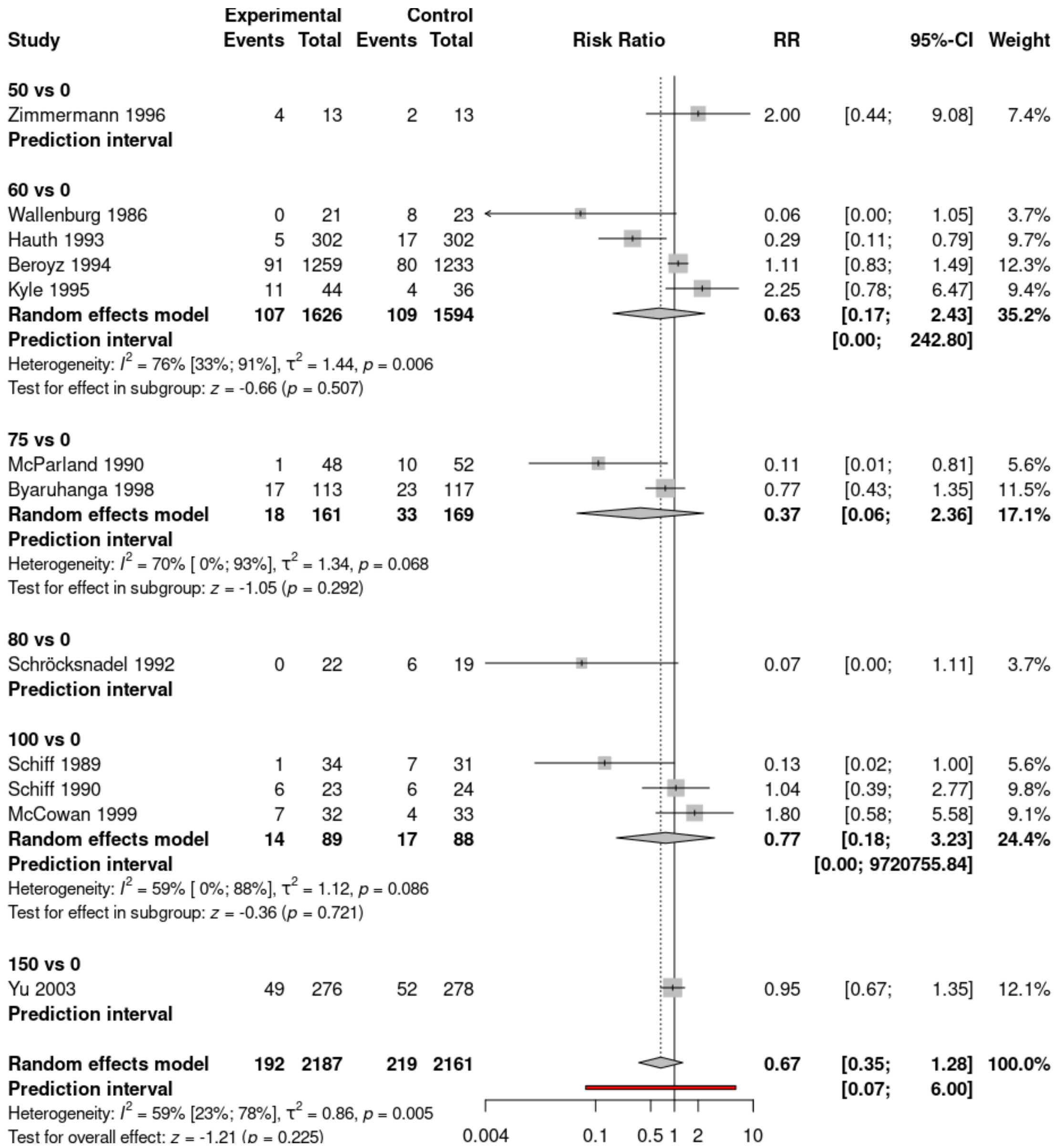

Supplementary Figure S21. Forest plot of the outcome **intrauterine growth restriction below the 10 percentile** with late initiated (>week 20) aspirin

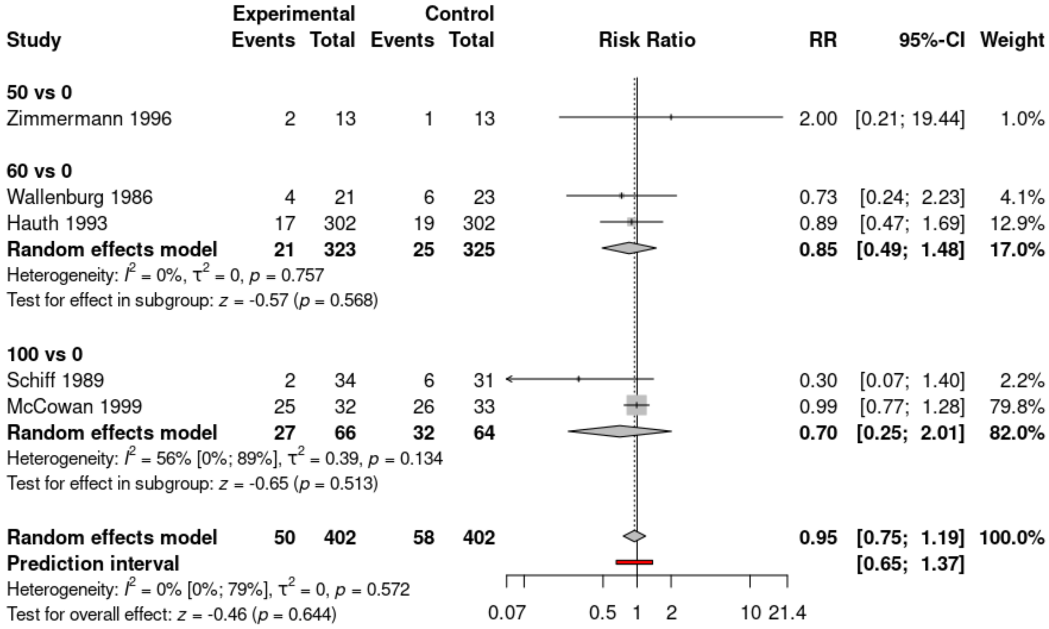

Supplementary Figure S22. Forest plot of the outcome **preterm birth before week 37** with late initiated (>week 20) aspirin

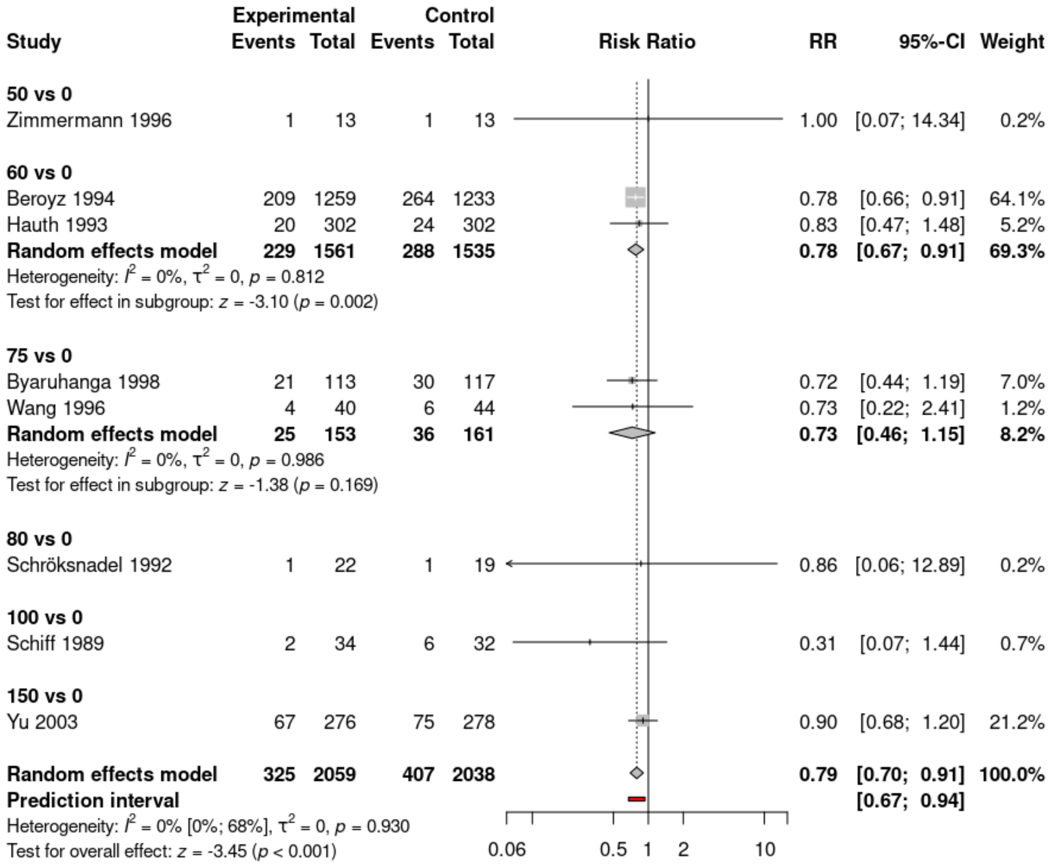

Supplementary Figure S23. Forest plot of the outcome **gestational age at delivery (in weeks)** with late initiated (>week 20) aspirin

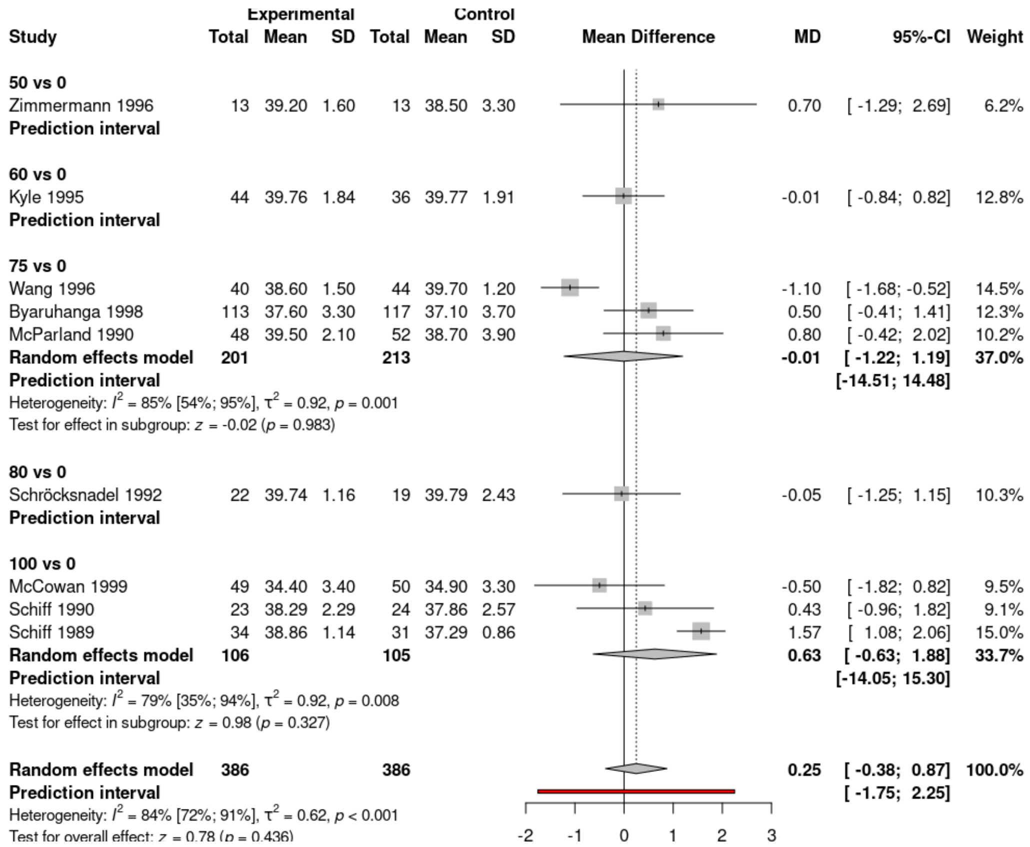

Supplementary Figure S24. Forest plot of the outcome **actual birth weight (in grams)** with late initiated (>week 20) aspirin

Study	Experimental Total	Experimental Mean	Experimental SD	Control Total	Control Mean	Control SD	MD	95%-CI	Weight
**50 vs 0**									
Zimmermann 1996	13	3199.00	487.00	13	3405.00	937.00	-206.00	[-780.04; 368.04]	1.0%
**Prediction interval**									
**60 vs 0**									
Kyle 1995	44	3252.00	472.00	36	3335.00	449.00	-83.00	[-285.39; 119.39]	8.3%
Hauth 1993	302	3249.00	577.00	302	3169.00	553.00	80.00	[-10.14; 170.14]	41.9%
**Random effects model**	**346**			**338**			**24.72**	**[-126.52; 175.97]**	**50.2%**
**Prediction interval**									
Heterogeneity: I^2 = 52% [0%; 88%], τ^2 = 6895.34, p = 0.149									
Test for effect in subgroup: z = 0.32 (p = 0.749)									
**75 vs 0**									
Wang 1996	40	3056.00	259.00	44	3040.00	416.00	16.00	[-130.80; 162.80]	15.8%
Byaruhanga 1998	114	2774.00	693.00	122	2694.00	866.00	80.00	[-119.49; 279.49]	8.6%
McParland 1990	48	3068.00	555.00	52	2954.00	852.00	114.00	[-165.78; 393.78]	4.3%
**Random effects model**	**202**			**218**			**49.93**	**[-58.99; 158.84]**	**28.7%**
**Prediction interval**								**[-656.14; 755.99]**	
Heterogeneity: I^2 = 0% [0%; 90%], τ^2 = 0, p = 0.781									
Test for effect in subgroup: z = 0.90 (p = 0.369)									
**80 vs 0**									
Schröcksnadel 1992	22	3336.00	395.00	19	3218.00	661.00	118.00	[-221.97; 457.97]	2.9%
**Prediction interval**									
**100 vs 0**									
McCowan 1999	49	1705.00	715.00	50	1787.00	678.00	-82.00	[-356.58; 192.58]	4.5%
Schiff 1990	23	2802.00	280.00	24	2772.00	294.00	30.00	[-134.10; 194.10]	12.6%
**Random effects model**	**72**			**74**			**0.52**	**[-140.34; 141.39]**	**17.1%**
**Prediction interval**									
Heterogeneity: I^2 = 0%, τ^2 = 0, p = 0.493									
Test for effect in subgroup: z = 0.01 (p = 0.994)									
**Random effects model**	**655**			**662**			**42.37**	**[-15.96; 100.70]**	**100.0%**
**Prediction interval**								**[-28.01; 112.75]**	
Heterogeneity: I^2 = 0% [0%; 65%], τ^2 = 0, p = 0.822									
Test for overall effect: z = 1.42 (p = 0.155)									

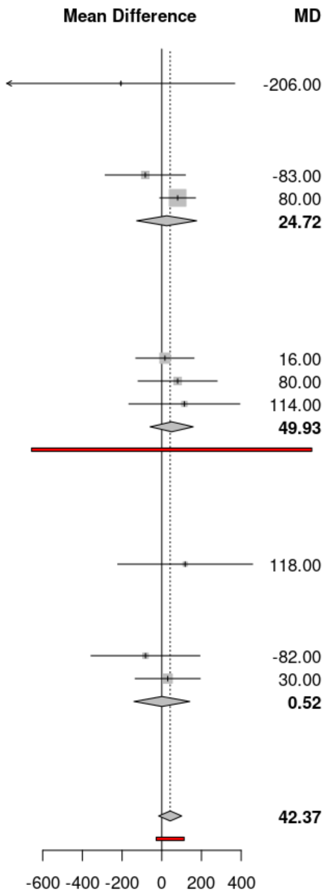

Supplement: Supplementary file 1 [file jcm-14-02134-s001.zip › jcm-3381029-supplementary.pdf]
